# Expression of a maize *SOC1* gene enhances soybean yield potential through modulating plant growth and flowering

**DOI:** 10.1038/s41598-021-92215-x

**Published:** 2021-06-17

**Authors:** Xue Han, Dechun Wang, Guo-qing Song

**Affiliations:** 1 Plant Biotechnology Resource and Outreach Center, Department of Horticulture, Michigan State University, East Lansing, MI 48824 USA; 2 Department of Plant Soil and Microbial Sciences, Michigan State University, East Lansing, MI 48824 USA

**Keywords:** Biotechnology, Plant sciences

## Abstract

Yield enhancement is a top priority for soybean (*Glycine max* Merr.) breeding. *SUPPRESSOR OF OVEREXPRESSION OF CONSTANS 1* (*SOC1*) is a major integrator in flowering pathway, and it is anticipated to be capable of regulating soybean reproductive stages through its interactions with other MADS-box genes. Thus, we produced transgenic soybean for a constitutive expression of a maize *SOC1* (*ZmSOC1*). T_1_ transgenic plants, in comparison with the nontransgenic plants, showed early flowering, reduced height of mature plants, and no significant impact on grain quality. The transgenic plants also had a 13.5–23.2% of higher grain weight per plant than the nontransgenic plants in two experiments. Transcriptome analysis in the leaves of 34-day old plants revealed 58 differentially expressed genes (DEGs) responding to the expression of the *ZmSOC1*, of which the upregulated *FRUITFULL* MADS-box gene, as well as the transcription factor *VASCULAR PLANT ONE-ZINC FINGER1*, contributed to the promoted flowering. The downregulated gibberellin receptor GID1B could play a major role in reducing the plant height. The remaining DEGs suggested broader effects on the other unmeasured traits (e.g., photosynthesis efficiency and abiotic tolerance), which could contribute to yield increase. Overall, modulating expression of *SOC1* in soybean provides a novel and promising approach to regulate plant growth and reproductive development and thus has a potential either to enhance grain yield or to change plant adaptability.

## Introduction

Soybean (*Glycine max* (L.) Merr.) is the world’s most important food legume to fight hunger and to feed a future with the growing population due to its high quality and high content of both protein and oil^1^. From 1994 to 2018, the world’s total hectare for soybean production increased at an average annual rate of 3.0%, the total production increased annually at 4.2%, and the yield per hectare increased at 1.2% (http://www.fao.org/faostat/en/#data/QC/visualize). Apparently, two main agronomic factors, including expansion of cropped land areas and yield increases, contributed the sustainable increase in soybean production in the past decades. Further expansion of cropped land areas becomes limited due to the decreasing availability of arable land (http://www.fao.org/3/y4252e/y4252e06.htm)^2–4^. Therefore, yield increases will be critical to meet the increasing demand of soybean over a wider range of environmental conditions for increasing populations worldwide^4^.

Crop yield is often determined by genotype and environment. Accordingly, mitigating of the negative environmental impact of climate change on crop yield can be achieved either by developing new agricultural practice to optimize plant growth conditions through or by breeding new genotypes for high yields^4^. For soybean, historical gains of new cultivars in grain yield over the past 84 years of breeding had been driven by linear increases in light interception and energy conversion and partitioning efficiencies^5^. The evidence of greater chlorophyll content and greater sink capacity late in the growing season in more recently released soybean varieties suggested that energy conversion/partitioning efficiencies were of importance for seed yield increase, although no consistent changes in photosynthetic capacity with 80 years of soybean breeding were observed^6^. Photosynthesis enhancement through breeding was considered a viable strategy to increase soybean yield due to their high positive genetic correlation^7^. Overall, enhancing photosynthetic efficiency of leaves, improving root traits for high nitrogen fixation and water usage efficiency, and increasing flower initiation and reducing flower abortion are considered to be the major genetic solutions to increase soybean yield^7–9^. In addition, increase in adaptability of soybean cultivars is desirable for expending soybean production area. Alternatively, similar to other legume crops, enhancing plant resilience to abiotic and biotic stresses is anticipated to reduce yield losses^10–12^.

Transgenic soybean is a major commercialized biotech crop. In the Approval Database of genetically modified (GM) crops, 41 GM soybean events are listed, including 11 events with a GM trait of modified oil/fatty acid, one event “Verdeca HB4 Soybean” containing a sunflower (*Helianthus annuus*) *HAHB-4* gene for drought tolerance^13,14^, and one event containing *Arabidopsis thaliana* B-BOX32 domain gene (*BBX32*) for enhanced photosynthesis/yield^15,16^ (http://www.isaaa.org). Herbicide tolerance (HT) and insect resistance (IR) are the major GM traits. In 2018, 123.5 million hectares soybeans were planted in the world, of which 95.9 million hectares (78%) were transgenics with either HT (69.3 million hectares) or HT/IR (26.6 million hectares), total accounting for 50% of the global area of biotech crops (http://www.isaaa.org). Currently, neither of the HB4 soybean for abiotic tolerance nor the BBX32 soybean for enhanced photosynthesis/yield has been made available for commercialization (http://www.isaaa.org). Recent field tests suggested that the HB4 soybeans were able to increase seed yield in warm and dry environment by changing plant architecture and improving drought tolerance^14^.

Modulation of plant reproductive develop through genetic manipulation is a powerful approach to impact soybean yield^17^. As the only GM soybean for enhanced photosynthesis/yield in the GM Approval Database, BBX32 soybean contained an ectopically expressed *BBX32*, which could, at least, affect photoperiodic regulation of flowering through the interaction of B-box family proteins [e.g., CONSTANS-LIKE 3 (COL3) in *Arabidopsis*]^15,16,18,19^. In *Arabidopsis*, COL3 is a protein-binding partner of CONSTANS (*CO*)^18,20^, which interacts with two major downstream flowering pathway integrators, including FLOWERING LOCUS T (*FT*) and SUPPRESSOR OF OVEREXPRESSION OF CONSTANS 1 (*SOC1*). The *CO-FT-SOC1* module plays an essential role in plant reproductive development and flowering time control^21–23^. In soybean, ten *FT* homologs have been identified, of which functional analysis of *GmFT2a* and *GmFT5a* has been conducted in *Arabidopsis* and soybean^24,25^. As a member of MADS-box gene family, homologs of *SOC1* in soybean play significant roles in floral development and flowering time control^26–28^. Ectopic expression of either a soybean *SOC1* homolog *GmGAL1*/*GmSOC1* in *Arabidopsis* or a *GmSOC1*-*LIKE* in *Lotus corniculatus* promoted flowering^29,30^; however, functional analysis of these two genes in soybean has not been reported. *Arabidopsis* Agamous-like MADS-box protein AGL1 played an essential role in ovule development, seed coat development, and endosperm formation^31^. To our knowledge, soybean *AGL1* (*GmAGL1*) is the only MADS-box gene that has been studied in soybean through overexpression, which resulted in early flowering^32,33^.

Modulating expression of MADS-box genes have a potential to increase crop yield^34–36^. For example, increasing the expression of a homolog of the *Arabidopsis FUL*/*AGL8* gene of maize (*ZMM28*) enhanced maize grain yield in the field^37^. The SOC1 protein is a MIKC protein. In *Arabidopsis*, the *SOC1* gene is a positive regulator of the downstream *FUL*/*AGL8* gene^21^. Accordingly, enhancing expression of the *SOC1* gene may have a potential to increase crop yield at least through the *FUL*/*AGL8* gene. In literatures, constitutive expression of a maize (*Zea mays*) *SOC1* gene (*ZmSOC1* or *ZmMADS1*) resulted in accelerated flowering time and reduced overall plant height in both *Arabidopsis* and maize^38^. In this study, we transformed the *ZmSOC1* into soybean to examine the possible impact of a major MADS-box gene on soybean plant growth and flowering. Meanwhile, we conducted transcriptome analysis of transgenic and nontransgenic plants to reveal the overall impact of ectopic expression of *ZmSOC1* on expression of other associated genes in soybean. The results demonstrate that ectopic expression of a monocot (maize) derived *SOC1* is efficient to regulate plant reproductive development in a dicot (soybean) and thus has a potential either to enhance grain yield or to change plant adaptability.

## Materials and methods

### Vector construction

**Figure 1:**
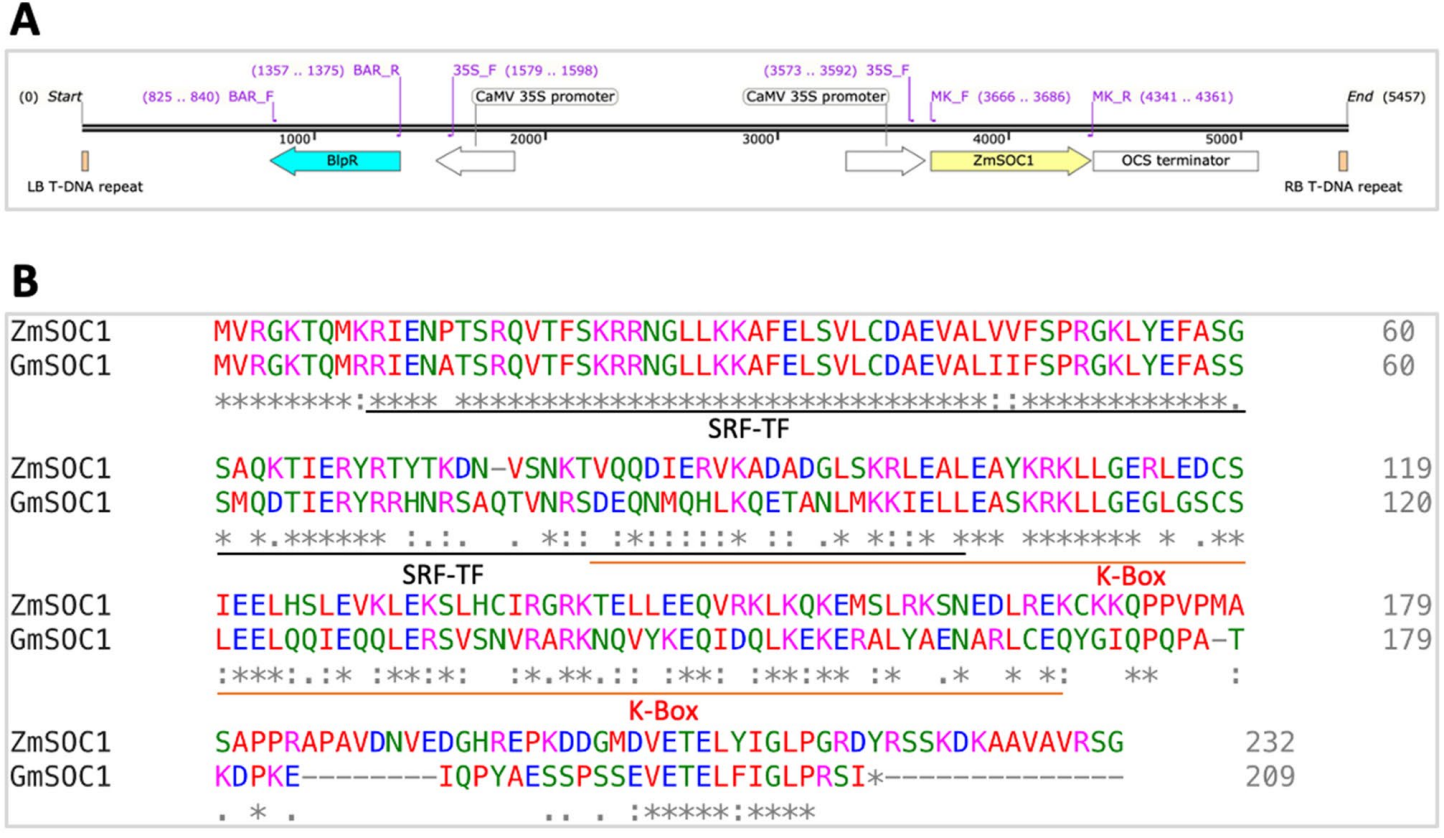
(**A**) Diagram of the T-DNA region of the pTF101.1-*ZmSOC1* vector. The primer positions are included in the diagram. *LB* left border, *RB* right border. (**B**) Protein sequence alignment of the ZmSOC1 and soybean’s GmSOC1 (iTAK: Glyma.18G224500.2_CDS). The protein sequence of the cloned *ZmSOC1* is identical to that derived from either the HQ858775.1 or a part of the NM_001111682.1. *GmSOC1* is the same as the sequence detected in the soybean transcriptome reference (cultivar Jack) (DN17539_c0_g1).

The sequence of the maize *SOC1* gene (*ZmSOC1* or *ZmMADS1*) has been published in GenBank (accession numbers: NM_001111682.1 and HQ858775.1)^38^. A 696-bp *ZmSOC1* derived from the cDNA of the maize inbred line B104 was cloned into the pKANNIBAL plasmid to form a *ZmSOC1* expression cassette CaMV 35S-*ZmSOC1-Ocs*, which was successfully inserted into the binary vector pTF101.1 to produce get pTF101.1-CaMV35S-*ZmSOC1-Ocs* (herein pTF101.1-*ZmSOC*: see the details in Fig. 1A)*.* Sanger sequencing data confirmed the *ZmSOC1* sequence. The pTF101.1-*ZmSOC1* was transformed into *Agrobacterium tumefaciens* strain EHA105. All recombinant DNA works were conducted in compliance with relevant institutional, national, and international guidelines and legislation.

### Plant transformation

Mature seeds of soybean cultivar Jack were used to produce cotyledonary node explants for transformation. Transformation and regeneration were conducted essentially by following the protocol by Pat and Wang^39^. About 400 explants were used in each transformation. The co-cultivation time was five days. After co-cultivation, the total number of shoots produced in 4 weeks and the total number of shoots elongated in 4–14 weeks were counted. After transferred to rooting medium, the number of elongated shoots produced roots was counted. All T_0_ plants were transferred to soil and grown in the greenhouse to produce T_1_ seeds for phenotyping T_1_ plants. Nontransgenic (NT) seedlings were used as a control. The plants were grown under a 16-h photoperiod at 21–30 °C. Phenotypic variations (e.g., date of the appearance of the first flower, the node number where the first flower appeared, the date of the appearance of the first pod, flower shape and structure, leaf color and leaf shape, plant architecture, and plant height) among the T_0_ plants were recorded. Young leaves, about 0.2 g for each greenhouse-growing plant, were harvested for DNA isolation and polymerase chain reaction (PCR) analysis of the transgenes. The transformation frequency was calculated as the percentage of the inoculated explants which produced the PCR positive T_0_ plants. The transformation experiment was repeated twice.

### Phenotypic evaluation of T_1_ and T_2_ plants

Early- and late-planted T_1_ plants were evaluated in two experiments. In experiment #1, the seeds were sowed on May 04, 2020, and the plants were growing in the greenhouse and moved to a secured field under natural environment conditions at East Lansing, Michigan on June 01, 2020. In experiment #2, the seeds were sowed on June 04, 2020 and the plants were growing in the field shared with the plants of experiment #1. Depending on the availability of the seeds, 1–40 seeds per line for all the T_0_ line/plant that produced seeds were sowed to evaluate T_1_ transgenic plants and to produce T_2_ seeds. 150 mg L^−1^ glufosinate ammonium (GS) was initially used to screen herbicide resistant transgenic plants by painting a half of a leaf along the midvein for each of the T_1_ seedlings. GS-resistant plants were transferred to one-gallon pots (20 cm diameter × 16 cm height). DNA from the GS-resistant plants was isolated for PCR analysis of the transgenes.

For phenotypic evaluation, the data collected for each plant included: (1) Date of seed germination; (2) Date of the appearance of first flower; (3) Node position where the first flower appeared; (4) Date of the first pod set; (5) Plant height measured and branch number and pod number counted at 141 for experiment #1 and 110 day for experiment #2 after sowing; (6) Pictures taken at different developmental stages; and (7) Seed protein, oil, and fiber contents were measured using a Grain Analyser (Infratec™ 1241, FOSS Analytical AB, Denmark). Fatty acids were extracted as described by Bubeck et al. from two seeds of each plant^40^. Fatty acid composition was determined by gas chromatography.

### Detection of transgenes

DNA was isolated from leaf tissues, 50–200 mg for each sample, using the cetyltrimethylammonium bromide (CTAB) method^41^. Three pairs of primers, bar-F and bar-R for the *bar* gene, and forward primers 35S-F (3′ portion of the *CaMV 35S* promoter) or MK-F and reverse primer MK_R for the *ZmSOC1* gene, were used to detect the presence of transgenes in each sample (Figure S1A), and GmAct11_F and GmAct11_R primers were used as a DNA quality control to detect soybean’s actin gene^42^ (Table S1). PCR reaction conditions for all primer pairs started with an initial denaturation for two min at 94 °C, 30 cycles of 45 s at 94 °C, 60 s at 60 °C and 90 s at 72 °C, and a final extension for 10 min at 72 °C. All amplified PCR products were separated on 1.0% agarose gel containing ethidium bromide and visualized and photographed under UV light.

### RNA sequencing and transcriptome analysis

The 5th young leaves near the shoot tips and exposed to sunshine at noon, 3–5 per plants, were harvested from 34-day old plants of three nontransgenic and six transgenic plants (i.e., 3 plants for each of the two transgenic lines) in experiment #2. The leaf samples were frozen immediately in liquid nitrogen, brought to lab, and stored at − 80 °C in a freezer for RNA isolation. Total RNA of each sample was isolated from about 500 mg leaf tissues using a CTAB method^43^ and was purified using RNeasy Mini Kit (Qiagen, Valencia, CA, USA). On-Column DNase digestion with the RNase-free DNase Set was used to remove DNA in the RNA samples (Qiagen, Valencia, CA, USA). RNA quality was determined using the High Sensitivity RNA ScreenTape system (Agilent technologies, Santa Clara, CA). High quality RNA with an RNA integrity number (RIN) equivalent greater than 5.0 was used for sequencing and reverse transcription (RT) PCR analysis. Reverse-transcription of 3–5 μg RNA to cDNA was performed using SuperScript II reverse transcriptase (Invitrogen, Carlsbad, CA, USA). Both regular RT-PCR and quantitative RT-PCR (qRT-PCR) using SYBR Green system (LifeTechnologies, Carlsbad, CA) were conducted to check the selected transcripts. The primers were designed according to the sequences of the RNA-seq (Table S1). QRT-PCR was performed on a Roche LightCycler^®^ 480 Instrument II (Roche). RT-PCR products were separated and visualized on 1.0% agarose gel containing ethidium bromide. The reaction conditions for RT-PCR were 94 °C for 2 min, 35 cycles of 45 s at 94 °C, 60 s at 62 °C and 60 s at 72 °C, with a final 10 min extension at 72 °C. The reaction conditions for qRT-PCR were 95 °C for 5 min, 45 cycles of 30 s at 95 °C, 45 s at 62 °C and 30 s at 72 °C. Transcript levels within samples were normalized to Actin. Foldchanges were calculated using 2^−∆∆Ct^, where ∆∆Ct = (Ct_TARGET_ – Ct_NOM_)_transgenic_ – (Ct_TARGET_ – Ct_NOM_)_nontransgenic_. Three biological samples and three technical replicates for each sample were used for the analysis of each transgenic and nontransgenic line.

The RNA samples were sequenced using the Illumina HiSeq4000 to generate 32–42 million 150 bp-paired end reads per sample. FastQC program (http://www.bioinformatics.babraham.ac.uk/projects/fastqc/) was used to assess the quality of sequencing reads for the per base quality scores. The reads with average scores greater than 38 were obtained and used for transcriptome analysis. A total of 72 million reads (MR) combined from a portion of the reads of the nine samples, 6.6–9.8 MR/sample, were assembled to develop a transcriptome reference using Trinity/2.8.5^44^. The paired reads were aligned to the transcriptome reference to estimate and the abundance for each of a single read. The differentially expressed transcripts (DETs) with the false discovery rate (FDR) value below 0.05 were identified using the Trinity command “run_DE_analysis.pl --method edgeR”^44^. Pfam proteins were used to annotate the transcriptome reference.

Cytoscape 3.8.2 was used to construct gene networks of overrepresented gene ontology (GO) terms for the selected DETs under BiNGO’s default parameters with selected ontology file ‘GO_Full’ and selected organism ‘*A. thaliana*’^45,46^.

### Statistical analysis

Statistical analysis of the phenotypic data was conducted using ANOVA and TukeyHSD in RStudio (Version 1.0.136).

## Results

### Cloning of the *ZmSOC1*

The pTF101.1-*ZmSOC1* contains a streptomycin/spectinomycin aminoglycoside adenylyltransferase gene (*aadA*) for bacterium selection and a *bialaphos resistance* (*bar*) gene under the CaMV 35S promoter conferring resistance to herbicide glufosinate (GS) for selection of transformed plant cells. In the 696-bp *ZmSOC1* sequence, 694 bp are identical to the published 696-bp reference deposited in the GenBank (accession numbers HQ858775.1 and NM_001111682.1). The protein sequence of the cloned *ZmSOC1* is identical to that derived from either the HQ858775.1 or a part of the NM_001111682.1. It has a 54.8% identity to the soybean’s SOC1 gene (*GmSOC1*) (iTAK: Glyma.18G224500.2_CDS), which is the sequence detected in the soybean transcriptome reference (cultivar Jack) assembled in this study (DN17539_c0_g1) (Fig. 1B).

### Phenotypic variations in T_0_ transgenic plants

**Figure 2:**
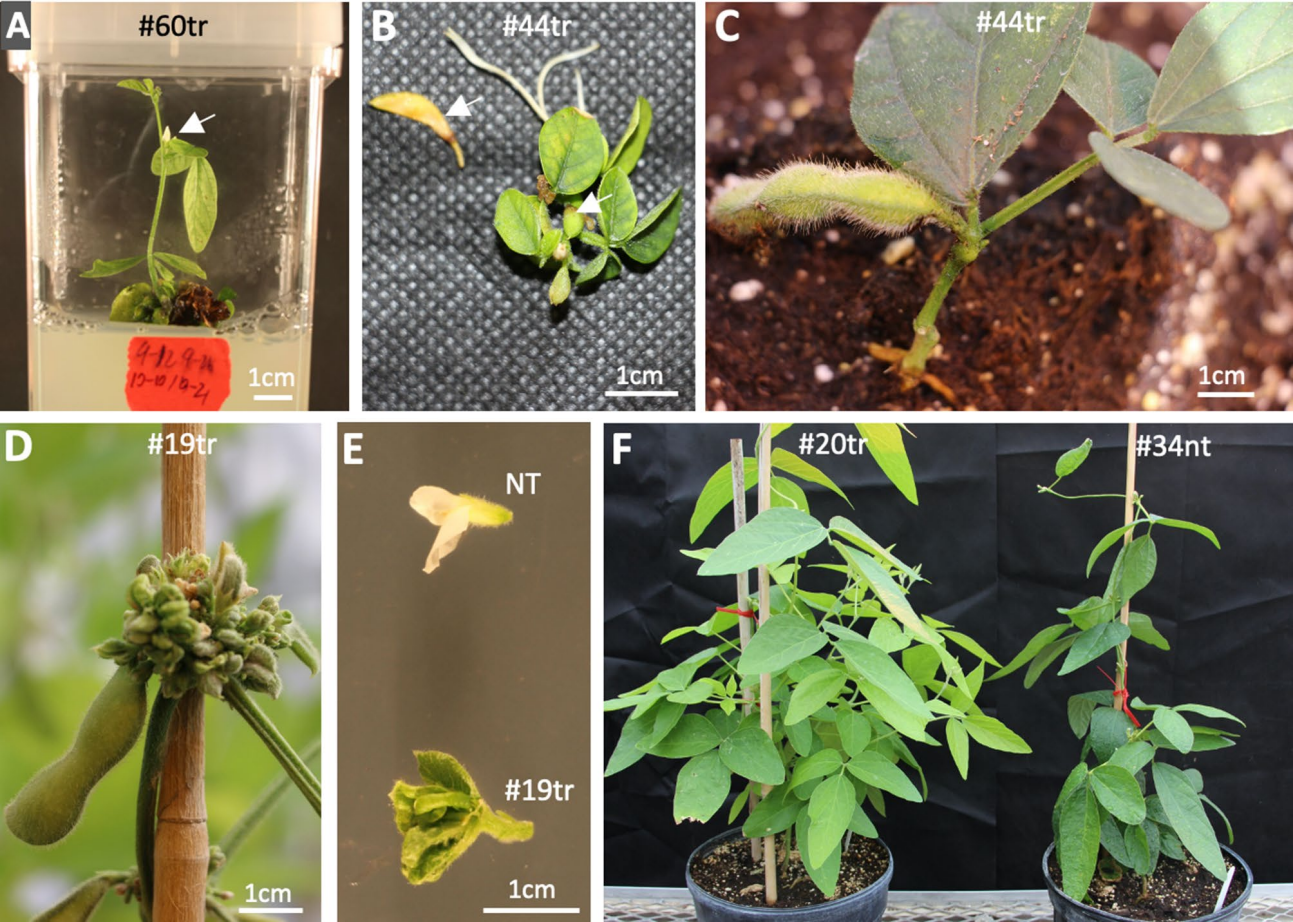
Phenotypic variations of T_0_ transgenic ZmSOC1-OX (TR) and nontransgenic (NT) soybean plants cv. Jack grown under in vitro and greenhouse conditions. (**A**) Flowering of a TR line #60tr growing on the elongation medium. (**B**) Seed pod production from a TR line #34tr growing on the rooting medium. (**C**) The #34tr plant showed dwarfing, no branches, and produced a seed pod in 27 days after transferred to soil. (**D**,**E**) A representative TR line (#19tr) showing abnormal flowers. (**F**) A TR line #20tr produced more branches than a NT line #34nt. Arrows show flowers or seed pods.

‘Jack’ was used to produce transgenic soybean plants because it was a transformable soybean cultivar using the *bialaphos resistance* (*bar*) gene as a selectable marker^39,47^. Herbicide-resistant plants were induced for half-seed explants after 2–4-week selection, 6–12-week elongation, and 2–4-week rooting (Figure S1). Of the total of 770 explants inoculated, 38 T_0_ transgenic lines/plants were produced in two experiments with transformation frequencies of 3.9% and 5.9%, respectively (Table S2). Remarkably, three of the 38 lines flowered and further formed seed pods during in vitro cultures (Fig. 2A,B).

Of the 38 T_0_ transgenic lines growing in the greenhouse under a 16-h photoperiod, 11 lines had no visible difference from the nontransgenic control and the rest 27 lines showed various phenotypic changes, including abnormal flowers or early flowering in seven lines, chimeric or increased numbers of branches in eight lines, and dead plants of two lines (Fig. 2C–I, Table S3; Figure S2). The lines with abnormal flowers or early flowering, which were rarely observed in our previous soybean transformations using other genes, were likely induced by the expression of the *ZmSOC1*, although the effect of tissue culture could also contribute to these phenotypic changes. Overall, 33 T_0_ transgenic lines produced seeds, and the lines with abnormal flowers produced no or a few seeds.

### Phenotypic evaluation of T_1_ transgenic plants

Seedlings from 21 lines were screened in two experiments to identify transgenic plants for phenotyping. Herbicide-painting was effective in detecting the *bar*-expressing transgenic plants (Figure S3). Polymerase chain reaction (PCR) analyses was reliable to detect the transferred *ZmSOC1* gene. Fourteen transgenic lines produced at least one transgenic seedling, and all the tested seedlings from the other seven lines were nontransgenic (Table S4). As anticipated, not all T_0_ transgenic lines were able to produce transgenic seeds due possibly to the chimeric nature of the transgenic plants produced from cotyledonary node explants. Unsurprisingly, only two lines showed a segregation rate of about 3:1 between transgenic and nontransgenic seedlings in the 14 transgenic lines (Table S4).

**Figure 3:**
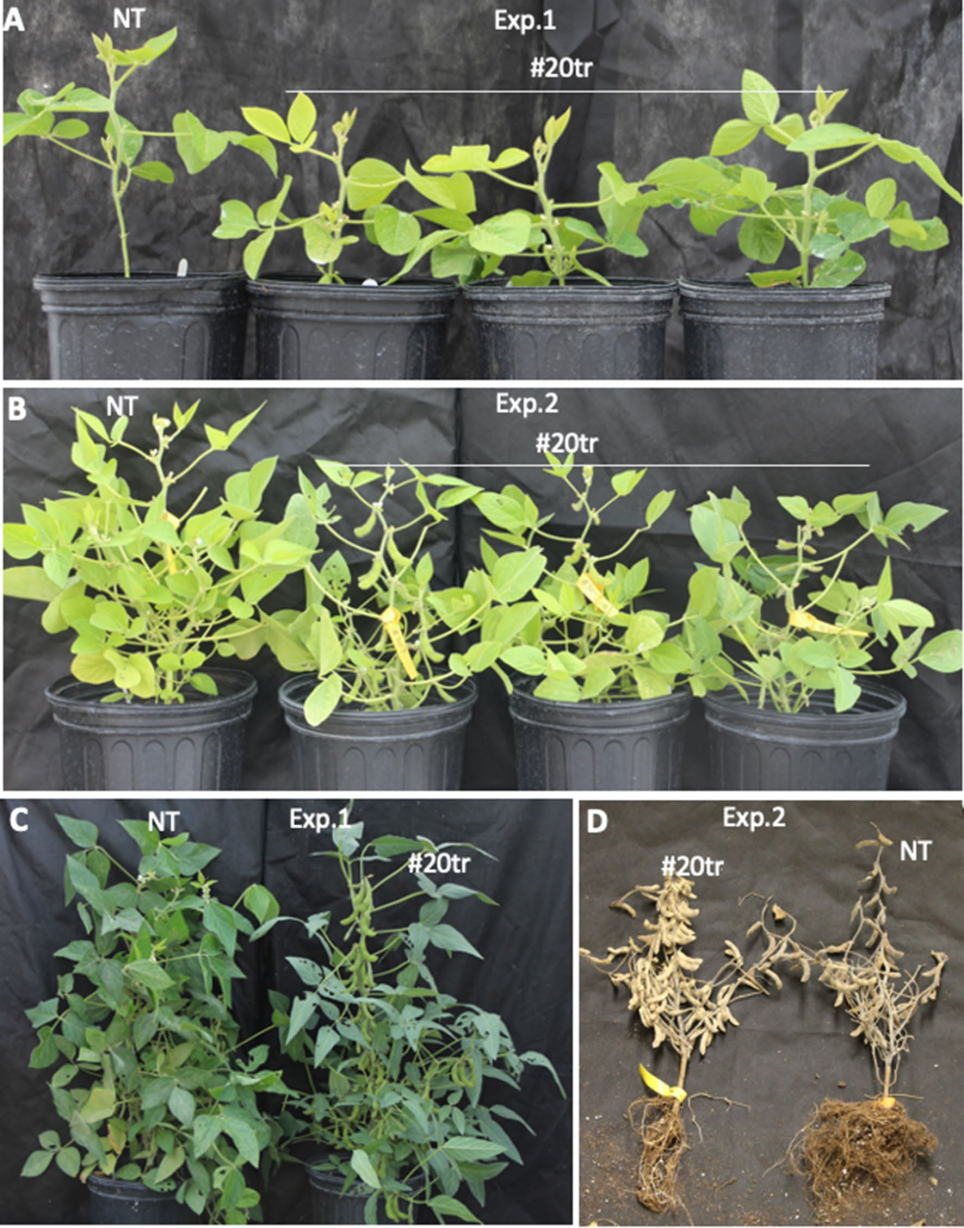
Growth of nontransgenic (NT) and transgenic soybean plants from a representative, T_1_ transgenic (TR) line expressing the *ZmSOC1*. All the plants were grown under natural environmental conditions. (**A**) Flowering of the 35-day old plants in the experiment #1. (**B**) Pod formation in the 67-day old plants in the experiment #2. (**C**) Seed pod production in the 134-day old plants in the experiment #1. (**D**) Seed pod production in the harvested plants in the experiment #2.

**Figure 4:**
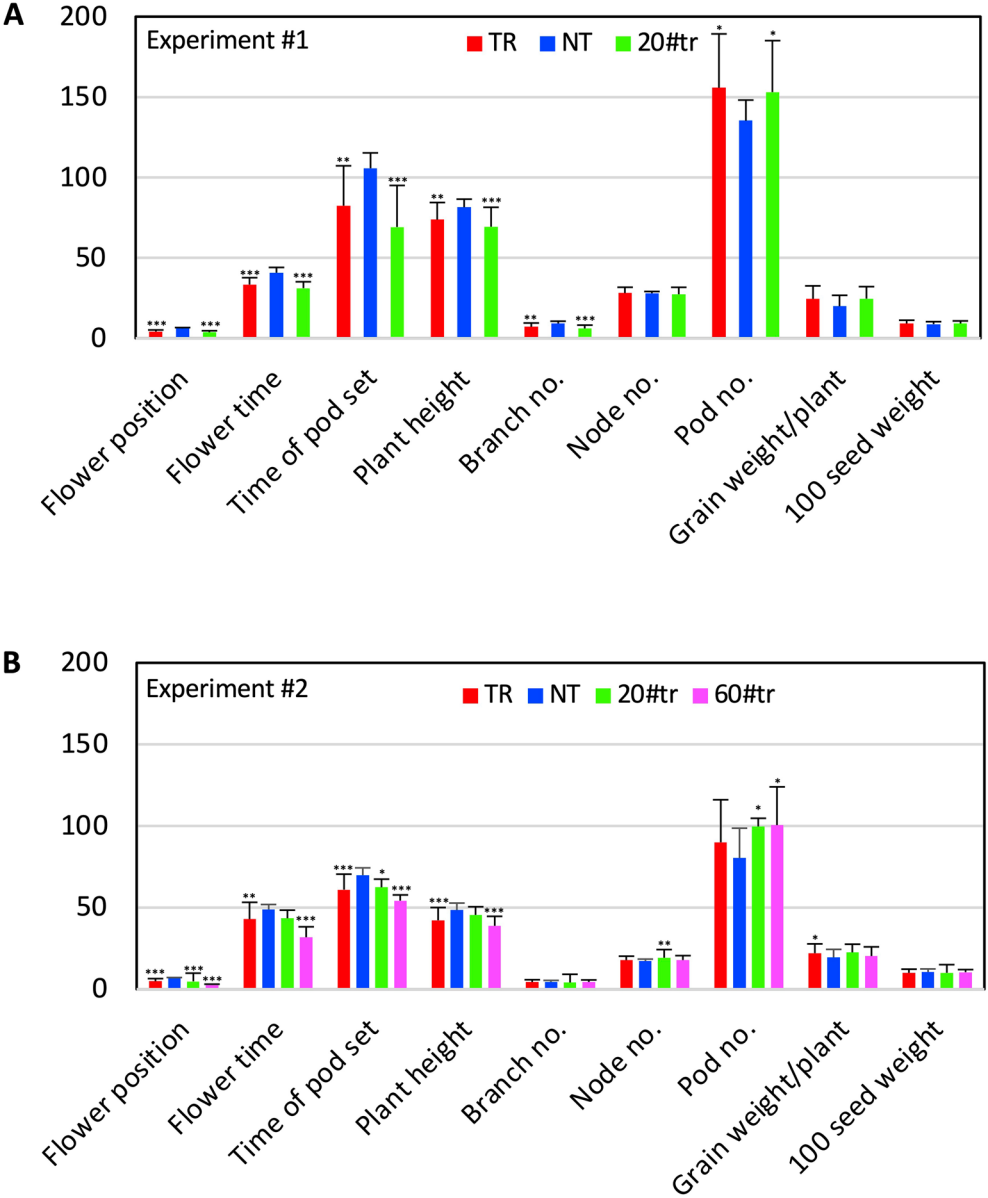
Phenotypic changes in T_1_ transgenic (TR) soybean plants expressing the *ZmSOC1* in the two experiments, where the seeds were sowed on May 4th in the experiment #1 and June 4th in the experiment #2, respectively. #20tr and #60tr are two independent TR lines. Y-axis showed mean value and STDEV of 16 nontransgenic (NT), 52 TR, and 16 #20tr plants in the experiment #1 (**A**) and 32 NT, 44 TR, 11 #20tr, and 7 #60tr lines in the experiment #2 (**B**). Flower position: The node number where the first flower appeared. Flowering time: Days when the first flowered appeared after the seeds were sowed. Time of pod set: Days when the first pod was set after the seeds were sowed. Plant height (cm): The height of the mature plant. Branch no., Node no., and Pod no.: The numbers for each mature plant were counted. Grain weight/plant (g): Weight of dry seed. 100 seed weight (g): 100 seed weight for each plant. For each of the nine traits, statistical analysis was conducted between NT and TR (or any of the individual TR line) separately. Significance codes: ***P < 0.001, **P < 0.01, *P < 0.05.

Plants from 10 transgenic lines were grown for phenotyping in each of the two experiments, including five transgenic evaluated in both experiments (Table S4). According to the local light length (https://www.timeanddate.com/sun/usa/lansing), the early and late-planting experiments were conducted by sowing the seeds on May 4th and June 4th, respectively, because soybean plants are photoperiod-sensitive for flowering. In fact, the variations in the environmental conditions (i.e., light and temperature) of the two experiments affected several traits of both transgenic and null segregants (hereafter: nontransgenic). For example, the early-planted nontransgenic plants in experiment #1, compared to the late-planted nontransgenic plants in experiment #2, were taller (81.5 cm vs. 48.5 cm), flowered earlier (41 days vs. 49 days), and set pods later (106 days vs. 70 days) (Fig. 4, Table S5). In both experiments, transgenic plants, compared to nontransgenic plants, showed an early reproductive phase with early flowering (i.e., 6 and 7 days earlier in the experiment #1 and #2, respectively), early pod set (i.e., 24 and 8 days earlier), and lower node positions (~ 2 nodes lower in both experiments) where the first flower appeared (Figs. 3B, 4). Additionally, the mature transgenic plants were 9.5% and 13.0% shorter than the nontransgenic ones in the experiment #1 and #2, respectively, but in each of the two experiments there was no difference in the total number of nodes between the transgenic and the nontransgenic plants, suggesting the reduced plant height was due to the reduced internode length (Fig. 4). This consistency suggested that expression of the *ZmSOC1* was able to enhance reproductive production and reduce plant height. On the other hand, the early-planted plants in the experiment #1 were taller and had more seed pods that the late-planted plants in the experiment #2 due to the difference in photoperiod and maybe temperatures too. In both experiments, the transgenic plants had more seed pods and seed production, including a nonsignificant (*P* = 0.053) 23.2% of increase of the grain weight per plant in the experiment #1 and a significant (*P* = 0.040) 13.5% of increase in the experiment #2 (Figs. 3C,D, 4).

Seed quality of nontransgenic and ten transgenic lines was evaluated by measuring eight quality parameters. When the ten transgenic lines were compared as one transgenic group to the nontransgenic seeds, no significant difference between the transgenic and the nontransgenic groups was found for all of the 10 seed quality parameters, suggesting that the expression of *ZmSOC1* had little impact on grain quality (Table 1).

**Table 1:** Effect of ZmSOC1 expression on seed quality. Protein, fiber and oil contents were measured using a Grain Analyzer. Fatty acids were determined by gas chromatography. The mean value (± STDEV) for each transgenic and nontransgenic (NT) line, were for the seeds from 2–4 transgenic plants except that all_tr is the average of all transgenic lines. Each transgenic line and all_tr were compared with the nt. *NA* not available. Signif. codes: ***P < 0.001, **P < 0.01, and *P < 0.05.

Genotype	Protein (%)	Fiber (%)	Oil (%)	Palmitic (%)	Stearic (%)	Oleic (%)	Linoleic (%)	Linolenic (%)
#14tr	42.03 ± 0.68	5.70 ± 0.17	16.13 ± 0.95	13.6 ± 0.95	3.86 ± 0.51	17.6 ± 0.69	55.83 ± 1.62	9.11 ± 1.33
#16tr	42.52 ± 2.52	5.62 ± 0.09	17.95 ± 0.32*	11.96 ± 0.72	3.71 ± 0.27	18.21 ± 2.25	57.34 ± 1.99	8.77 ± 0.93
#20tr	41.96 ± 1.45	5.67 ± 0.19	17.44 ± 1.11	12.5 ± 0.59	3.75 ± 0.32	17.51 ± 2.71	57.11 ± 2.22	9.15 ± 1.28
#21tr	41.74 ± 0.27	5.61 ± 0.03	15.94 ± 1.59	13.81 ± 0.86	3.73 ± 0.35	17.82 ± 2.45	55.8 ± 2.46	8.85 ± 0.82
#22tr	41.27 ± 0.63	5.81 ± 0.07	16.21 ± 0.80	13.30 ± 2.27	3.96 ± 0.22	16.42 ± 4.43	59.07 ± 0.57	7.25 ± 1.38
#25tr	44.03 ± 0.33*	5.59 ± 0.04	16.11 ± 0.30	15.41 ± 1.13	3.94 ± 0.27	17.63 ± 0.77	54.8 ± 0.35	8.21 ± 0.26
#28tr	42.28 ± 1.00	5.81 ± 0.03	18.13 ± 0.23**	NA	NA	NA	NA	NA
#35tr	41.33 ± 1.51	5.95 ± 0.09	17.26 ± 0.68	15.59 ± 0.7	3.54 ± 0.35	16.58 ± 2.62	55.27 ± 1.84	9.00 ± 0.66
#4tr	42.37 ± 1.50	5.54 ± 0.17	15.29 ± 0.37	12.28 ± 0.68	3.78 ± 0.03	17.27 ± 2.1	56.97 ± 0.4	9.71 ± 1.06
#60tr	33.07 ± 2.32**	6.28 ± 0.20*	18.44 ± 0.37**	15.91 ± 1.81	3.69 ± 0.22	16.27 ± 1.29	55.22 ± 0.98	8.91 ± 0.72
all_tr	41.41 ± 1.55	5.65 ± 0.27	17.30 ± 0.04	13.4 ± 1.56	3.72 ± 0.33	17.41 ± 2.33	56.53 ± 2.14	8.94 ± 1.04
nt	41.27 ± 3.07	5.75 ± 0.24	16.84 ± 1.24	13.47 ± 1.43	3.71 ± 0.32	17.10 ± 1.51	56.88 ± 1.34	8.85 ± 1.04

### Phenotypic evaluation T_2_ transgenic plants

T_2_ plants were grown to identify homozygous transgenic lines and to bulk homozygous seeds for further tests. Meanwhile, flowering and pod formation time of the greenhouse-growing plants were evaluated. Early flowering and early pod formation were observed in the T_2_ transgenic plants. This provided further evidence that the expression of *ZmSOC1* enhanced reproductive development.

### Differentially expressed (DE) transcripts (DETs) responding to *ZmSOC1* expression

**Figure 5:**
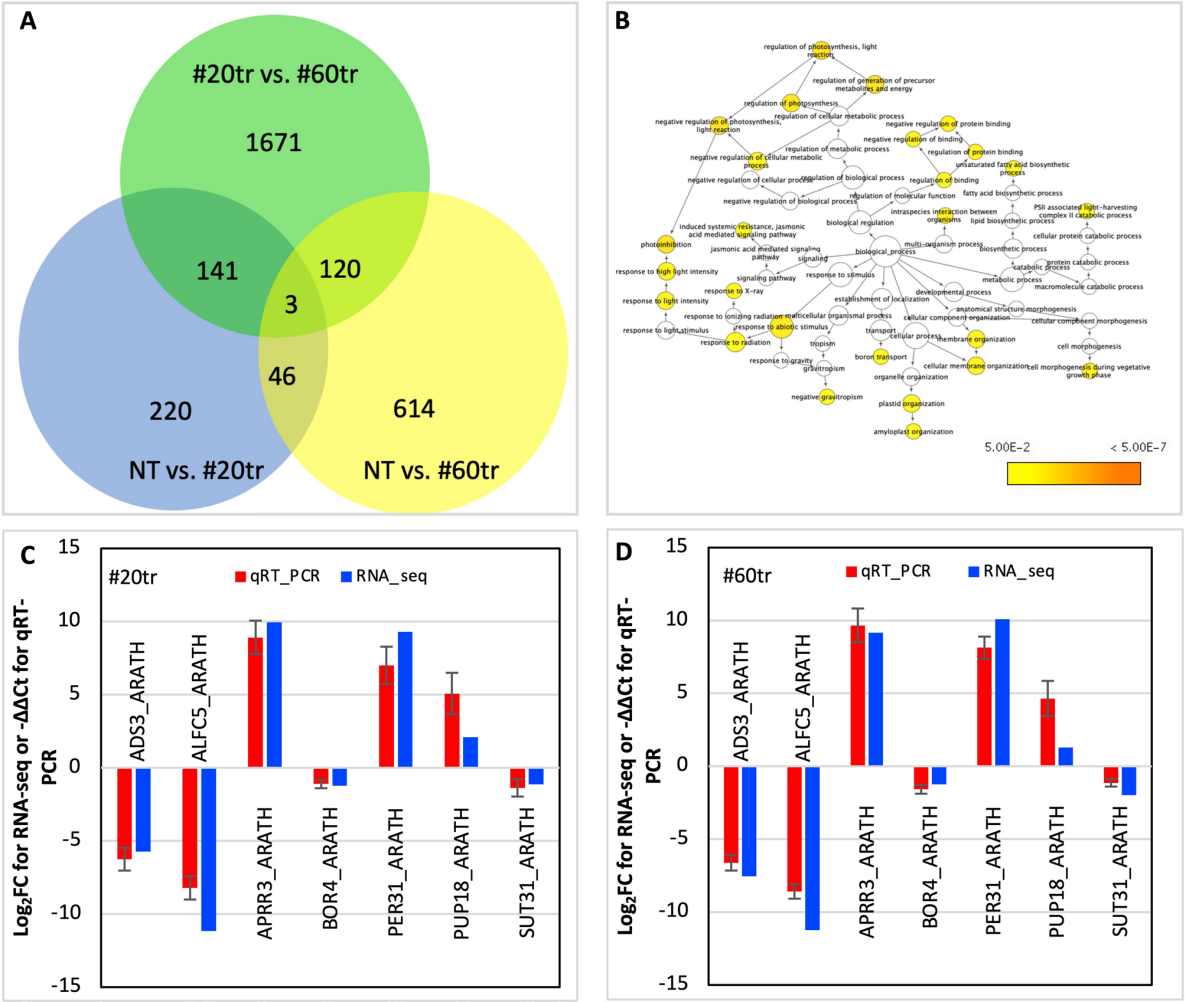
Transcriptome analysis in leaves among T_1_ nontransgenic (NT) and two soybean transgenic lines of #20tr and #60tr. (**A**) Venn diagram illustrating overlap of the three transcriptomic comparisons of the annotated, differentially expressed transcripts (DETs). (**B**) Gene networks of differentially expressed genes shared in leaf tissues of transgenic lines of #20tr and #60tr. The ontology file of GO_full in BiNGO was used to identify overrepresented GO terms (*p* < 0.05). Bubble size and color indicate the frequency of the GO term and the P-value, respectively. (**C**,**D**) Comparison of the RT-qPCR analysis result and the RNA-seq data of the selected DETs. − ∆∆Ct is an average of three biological and three technical replicates for each DET. GmActin 1 (SAC1_ARATH) was used to normalize the RT-qPCR results.

Transcriptome analysis of transgenic and nontransgenic plants was conducted for two major purposes, to verify the expression of the transgenes and to reveal the genes that responding to the expression of the *ZmSOC1*. In the leaves of six plants from the two transgenic lines, high expressions of the *bar* and the *ZmSOC1* were found; in contrast, no sequence reads of the transgenes were detected in any of the three nontransgenic plants (Table 2). The results verified the expression of the two transgenes. Meanwhile, it showed that the expression of the transgenes in transgenic line #60tr was higher than that of transgenic line #20. The higher *ZmSOC1* expression in the line #60tr may explain, at least partially, the phenotypic variations as well as the difference of the 1935 DETs identified between the two transgenic lines (Fig. 5A; Table 2; Figure S2; Table S6).

**Table 2:** Differentially expressed genes (DEGs) overlapped in two transgenic lines (#20tr and #60tr), each compared to the nontransgenic line. #N/As represent a non-DEGs.

Isoform ID	Log_2_(#60tr/nt)	Log_2_(#20tr/nt)	Log_2_(#60tr/#20tr)	Annotation_protein	e_value_annotation
DN18940_c0_g1_i5	− 11.85	− 11.78	#N/A	MDHC_MEDSA	0.0E+00
DN17886_c0_g1_i15^z^	− 11.23	− 11.16	#N/A	ALFC5_ARATH	3.7E−63
DN22071_c0_g1_i12	− 8.70	− 8.63	#N/A	TMK1_ARATH	0.0E+00
DN21378_c2_g2_i2	− 8.41	#N/A	− 7.65	RB3GP_DROME	7.75E−29
DN14711_c0_g1_i5	#N/A	7.36	#N/A	RB3GP_DANRE	8.6E−24
DN18476_c0_g3_i7	− 8.33	− 8.26	#N/A	WRK70_SOLLC	6.5E−37
DN17384_c1_g4_i8	− 8.14	− 8.07	#N/A	GID1B_ARATH	0.0E+00
DN20140_c1_g1	− 8.01	1.75	#N/A	MMSA_ARATH	0.0E+00
DN16057_c0_g1_i3	− 7.54	− 5.72	#N/A	ADS3_ARATH^z^	1.2E−172
DN19626_c0_g2	− 4.24	− 9.37	#N/A	RFP4B_DANRE	9.4E+00
DN13742_c0_g1	− 2.41	− 1.80	#N/A	C93C1_SOYBN	0.0E+00
DN22021_c0_g1_i7^z^	− 1.96	− 1.13	#N/A	SUT31_ARATH	0.0E+00
DN16671_c0_g1_i7	− 1.73	− 11.11	9.52	NRX3_ARATH	2.2E−171
DN14666_c0_g5_i2	− 1.46	− 0.99	#N/A	MIP1B_ARATH	4.2E−10
DN19591_c0_g3_i2	− 1.45	− 1.06	#N/A	PUB33_ARATH	1.6E−12
DN15732_c0_g1_i4^z^	− 1.24	− 1.22	#N/A	BOR4_ARATH	0.0E+00
DN16638_c0_g2_i7	− 1.21	− 1.19	#N/A	MBCD_SOLTU	0.0E+00
DN19977_c0_g1	− 1.20	11.22	#N/A	FTSH2_ORYSJ	1.6E−54
DN20831_c0_g2_i9	− 1.13	− 1.38	#N/A	WTR32_ARATH	6.6E−171
DN19207_c0_g4_i20	− 1.10	− 1.14	#N/A	ATPA_NEOSM	4.5E+00
DN18355_c0_g1	− 1.08	− 0.78	#N/A	KUA1_ARATH	3.4E−35
DN21645_c0_g1_i27	− 1.06	− 0.81	#N/A	TPPA_ARATH	0.0E+00
DN18779_c1_g3_i3	− 0.97	− 0.97	#N/A	RCA_VIGRR	1.1E−102
DN16210_c0_g4_i5	− 0.95	− 0.75	#N/A	DJC76_ARATH	1.3E−29
DN16424_c0_g1_i2	− 0.86	− 2.41	#N/A	CO9_TAKRU	1.4E+00
DN16521_c0_g2_i3^z^	1.29	2.10	#N/A	PUP18_ARATH	9.9E−01
DN19407_c0_g5_i1	1.65	5.28	1.55	14312_ARATH	5.4E−162
DN18907_c0_g1_i10	1.73	2.05	#N/A	SSG1_IPOBA	0.0E+00
DN17259_c0_g1	4.23	3.79	3.35	5MMP_ARATH	2.3E−38
DN15402_c0_g1_i2	4.71	#N/A	#N/A	CB23_SOYBN	0.0E+00
DN15174_c0_g1_i14	#N/A	− 1.17	#N/A	CB23_PEA	1.3E−175
DN20172_c0_g1_i11	4.87	4.76	#N/A	APA2_ARATH	0.0E+00
DN21013_c0_g1_i3	4.94	5.18	#N/A	CHLD_PEA	0.0E+00
DN18221_c0_g1	5.11	− 0.85	4.41	BBD1_ARATH	1.1E−141
DN15371_c1_g2_i9	5.12	4.47	#N/A	BH068_ARATH	1.5E−67
DN15819_c1_g1_i18	5.12	4.92	#N/A	RPP2B_ARATH	2.1E−04
DN21013_c0_g1_i1	5.90	5.59	#N/A	CHLD_PEA	0.0E+00
DN20112_c0_g1_i5	6.35	#N/A	#N/A	GIL1_ARATH	1.3E−40
DN19249_c0_g1_i14	#N/A	− 0.96	1.30	GIL1_ARATH	1.9E−67
DN14475_c1_g1_i3	6.72	6.16	#N/A	SEOA_ARATH	2.4E−102
DN16965_c0_g1	7.49	8.14	#N/A	PAP7_ARATH	2.0E−37
DN21148_c0_g3_i6	7.97	7.60	#N/A	VOZ1_ARATH	1.0E−56
DN19870_c0_g1_i5	8.09	7.90	#N/A	HSF24_SOLPE	6.2E−73
DN20857_c0_g1_i7	8.35	8.80	#N/A	RF298_ARATH	8.6E−02
DN19124_c0_g1_i3	8.45	8.00	#N/A	BABA2_DANRE	3.8E−18
DN22183_c0_g1_i13	8.60	#N/A	#N/A	FUCO2_ARATH	1.6E−96
DN22183_c1_g1_i16	#N/A	− 8.27	#N/A	FUCO2_ARATH	1.3E−163
DN19377_c0_g1_i6	8.66	8.87	#N/A	TSTC_DICDI	4.8E−06
DN17242_c0_g1_i10	8.88	8.38	#N/A	RS31_ARATH	1.9E−90
DN21974_c0_g1_i11	9.03	8.77	#N/A	DNLI4_ARATH	0.0E+00
DN15112_c1_g2_i5^z^	9.15	9.93	#N/A	APRR3_ARATH	4.7E−07
DN19594_c1_g4_i5	9.86	7.80	#N/A	SYP22_ARATH	3.5E−145
DN21349_c0_g1_i10	10.05	9.81	#N/A	TI100_ARATH	2.3E−134
DN18077_c0_g1_i8	10.07	9.24	#N/A	PAB_ARATH	1.8E−160
DN14378_c1_g1	10.08	7.41	#N/A	Y9096_DICDI	3.9E−06
DN14171_c0_g5_i1^z^	10.10	9.29	#N/A	PER31_ARATH	4.1E−153
DN21622_c0_g1_i26	10.17	10.89	#N/A	ACR3_ARATH	2.3E−166
DN14254_c0_g2	10.62	9.10	#N/A	FULL_VITVI	2.6E−89
DN7082_c0_g1_i1^y^	10.77	7.81	2.97	MAD50_ORYSJ, ZmSOC1	3.0E−119
DN20211_c1_g1_i9	10.91	11.01	#N/A	ESYT1_HUMAN	7.2E−13
DN16691_c0_g6_i5^y^	11.27	8.08	3.14	bar_gene	
DN21779_c0_g1_i16	11.28	10.91	#N/A	Y005_SYNY3	4.2E−39
DN19935_c0_g1_i1	11.51	#N/A	11.58	FERON_ARATH	0.0E+00
DN21094_c1_g10_i2	#N/A	6.78	#N/A	FERON_ARATH	2.9E−129

^z^The transcript verified by RT-qPCR. ^y^Transgenes.

410 and 782 DETs were identified in the two comparisons between each of the two transgenic lines (i.e., #20tr and #60tr) and the nontransgenic line, respectively (Fig. 5A). Of the annotated soybean genes, 58 DE genes (DEGs), including 31 upregulated, 25 downregulated DEGs and two other DEGs were shared in the two comparisons; the two DEGs (i.e., FUCO2_ARATH and FTSH2_ORYSJ) showed opposite changes in the two transgenic lines (Table 2). Using regular RT-PCR, expression of both the *bar* and the *ZmSOC1* was detected in the transgenic plants but was absent in the nontransgenic ones. In addition, the results of RT-qPCR analysis of seven selected DEGs were consistent with those from RNA-sequencing data (Table 2, Fig. 5).

Of the 31 upregulated genes detected in both the #20tr and #60tr lines, the *FUL-like* MADS-box gene (FULL_VITVI) is a downstream gene of the *SOC1*^22^; as expected, the expression of the transgene *ZmSOC1* promoted flowering in the transgenic soybean plants by enhanced the expression of a soybean’s *FUL-like* gene. Transcription factor VASCULAR PLANT ONE-ZINC FINGER1 (VOZ1_ARATH) is a positive regulator of plant flowering^48,49^, and it also binds to the cis-acting region of the PYROPHOSPHATE-ENERGIZED VACUOLAR MEMBRANE PROTON PUMP 1 gene (*AVP1*) which regulates auxin-mediated organ development and enhance NaCl and drought tolerance^50,51^. The upregulated VOZ1 resulted in an increased expression of the *AVP1* in the #60tr but not in the #20tr line (Table S4). PEROXIDASE 31 (PER31_ARATH) has multifunction related to lignin, auxin catabolism, abiotic stresses. Protein IN CHLOROPLAST ATPASE BIOGENESIS (PAB_ARATH) is required for plant flowering and seed production^52^. TWO-COMPONENT RESPONSE REGULATOR-like APRR3 (APRR3_ARATH) regulates photoperiodic flowering response^53^. Chloroplastic, magnesium-chelatase subunit ChlD (CHLD_PEA) is involved in chlorophyll biosynthesis. Protein TIC 100 (TI100_ARATH) plays an important role in chloroplast biogenesis and embryo development in ending seed dormancy^54^. ASPARTIC PROTEINASE A2 (APA2_ARATH) is involved in lipid metabolic process. GRANULE-BOUND STARCH SYNTHASE 1(SSG1_IPOBA) catalyzes the synthesis of amylose. HEAT SHOCK FACTOR PROTEIN HSF24 (HSF24_ARATH) is a DNA binding protein. The 21 downregulated DETs included six genes expressed only in the nontransgenic line (i.e., MDHC_MEDSA, ALFC5_ARATH, TMK1_ARATH, WRK70_SOLLC, RL10_EUPES, GID1B_ARATH), 15 expressed in both the transgenic and nontransgenic lines (Table 2). CYTOPLASMIC MALATE DEHYDROGENASE (MDHC_MEDSA) is involved in carbohydrate metabolic process. FRUCTOSE-BISPHOSPHATE ALDOLASE 5 (ALFC5_ARATH) hydrolyzes the fructose 1–6-bisphosphate to fructose 6-phosphate. Receptor protein kinase TMK1 (TMK1_ARATH) functions in auxin signal transduction^55^. WRKY DNA-binding transcription factor 70 (WRK70_SOLLC) modulates various phytohormones signals and affects senescence, biotic and abiotic stress responses^56^. Gibberellin receptor GID1B (GID1B_ARATH) is a soluble gibberellin (GA) receptor redundant with GID1A and GID1C that control root growth, seed germination, and flower development through GA signaling; loss-of-function of these receptors can lead to plant dwarfing^57–59^. CHLOROPLASTIC PALMITOYL-MONOGALACTOSYLDIACYLGLYCEROL DELTA-7 DESATURASE (ADS3_ARATH) catalyzes desaturation of fatty acid^60,61^ (Table 2). 60S RIBOSOMAL PROTEIN L10 (RL10_EUPES) is a component of the small ribosomal subunit. The 58 upregulated and downregulated DEGs could be responsible, at least in part, for the phenotypic changes of the *ZmSOC1*-expressing plants. The other two shared DEGs along with those non-shared DETs likely contributed to the differences between the two transgenic lines (Table 2, Table S6).

Gene ontology (GO) analysis of the 58 shared DEGs, which were consistently upregulated or downregulated in both transgenic lines, resulted in a total of 41 overrepresented GO terms (*P* < 0.05) in three networks, including 26 in “biological process”, 18 in “molecular function”, and six in “cellular component” (Fig. 5B, Figure S4). These overrepresented GO terms in the three networks revealed the multi-faceted effect of *ZmSOC1* expression in the two soybean transgenic lines.

## Discussion

In this study, a maize MADS-box gene *ZmSOC1*, which has a 54.8% identity to the soybean’s *GmSOC1* at the protein level, was constitutively expressed in soybean cultivar Jack. We found that the constitutively expressed *ZmSOC1* promoted flowering and reduced plant height, suggesting that the monocot-derived *ZmSOC1* functions similarly to the *SOC1* in *Arabidopsis* as a flowering pathway integrator^21^. Furthermore, we found that the transgenic plants had a 23.2% and a 13.5% of higher grain weight per plant than the nontransgenic plants in the two experiments, respectively. The DEGs responding to the expression of the *ZmSOC1* suggested broader effects of the *ZmSOC1* on plant height, flowering time, and the other unmeasured traits (e.g., photosynthesis efficiency and abiotic tolerance), which could contribute to yield increase. This is the first report that a constitutively expressed *SOC1* gene was evaluated in soybean. We used the *ZmSOC1* instead of the *GmSOC1* in this study because it was easier to detect integration and expression of the transgene *ZmSOC1* than that of a transferred endogenous *GmSOC1* in transgenic soybean plants.

### *ZmSOC1* expression hastens soybean flowering

This is the first demonstration that a major flowering pathway integrator *SOC1* from maize functioned in soybean. Unsurprisingly, the expression of the *ZmSOC1* promoted soybean plant flowering because the *ZmSOC1* is similar to the *GmSOC1*, which is a *SOC1* homology that functions as a positive regulator for plant initiation and flowering^21,29,30,33,38^. Interestingly, more phenotypic diversities were observed in the T_0_ plants than the T_1_ plants because of the differences in the genetic background of different transgenic lines as well as the culture condition for the plants. Remarkably, the type of the altered flowers appeared only in T_0_ transgenic plants although these abnormal flowers produced no seeds. In this particular case, the abnormal flowers could be caused by the transgene expression because none of the T_0_ nontransgenic plants had similar abnormal flowers. In general, T_0_ transformants of soybean obtained through the transformation of half-seed explants were often chimeric, which made the T_0_ transformants not convincing transgenic targets for phenotyping.

Overexpression of MADS-box gene *GmAGL1* was more effective in hastening soybean plant (cv. Jack) flowering under long-day than short-day conditions^32^. In this study, regardless of the photoperiod variations in the two experiments, the transgenic plants flowered earlier than the nontransgenic plants. The plants grown under short-day conditions in experiment #1 flowered earlier and set pods much later than those under the long-day conditions in experiment #2. The low temperatures during the early development stage in the experiment #1 could have delayed pod set, which could in turn contribute to taller and more branches for each plant observed in the experiment #1 than those in the experiment #2.

### *ZmSOC1* expression reduces soybean size

*ZmSOC1* overexpression reduced the height of maize transgenic plants^38^. Similarly, the ectopic expression of the *ZmSOC1* resulted in the reduction of the transgenic soybean plants. This provides further evidence to demonstrate that manipulation of the expression of a *SOC1* homolog is an effective approach to change plant architecture.

### Effects of the *ZmSOC1* expression on soybean yield and grain quality

Both yield and seed quality are determined by the interactions of genetic and environmental factors. Overexpression of the *GmAGL1* in two transgenic lines promoted plant maturity but had no trade-off of yield and grain quality^32^. In this study, T_1_ transgenic plants from ten transgenic lines were used to expand the genetic diversity of the transgenic plants, meanwhile, the plants were evaluated under two environmental. It was exciting and convincing that the transgenic plants had a higher yield than the nontransgenic plants grown in the pots although field trials are still needed to evaluate homozygous transgenic plants.

### Genes and gene networks responding to the expression of the *ZmSOC1*

**Figure 6:**
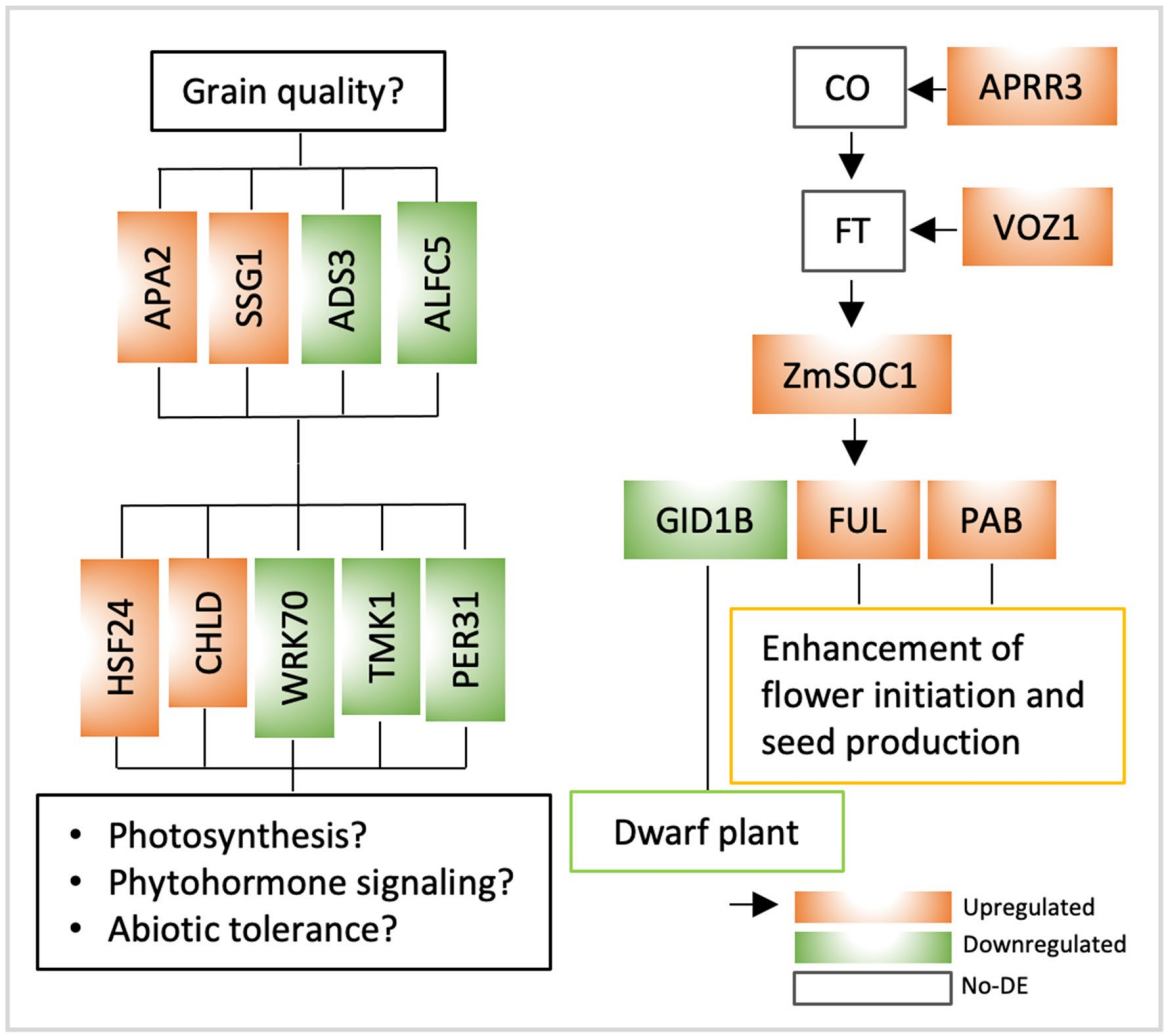
Potential effects of the expression of the *ZmSOC1* on soybean plant growth and development revealed by the shared differential expressed genes (DEGs) identified in leaves of two transgenic lines (i.e., #20tr and #60tr) (Table 2).

We used RNA sequencing data of two transgenic lines to identify the differentially expressed genes associated with the expression of the *ZmSOC1* expression. Although the data only represented the transcriptome at the moment of sampling, the information of the DEGs was very useful to reveal the molecular mechanism that underpinned the phenotypic changes in the transgenic plants (Table 2, Fig. 6). For example, in addition to the upregulated expression of the *APRR3* and *VOZ1*, and *PAB*, the upregulated *FUL* gene (FULL_VITVI) was a direct molecular evidence to support the enhanced floral initiation and early flowering in the transgenic plants. The increased expressions of *APRR3* and *VOZ1* were not associated with the increased *CO* or *FT* due possible to the strong expression of both genes in the leaf tissues harvested for sequencing at noon when the sunshine was strong. The downregulated *GID1B* was the best indicator for the reduced plant size of the transgenic plants because of the known function of the *GID1B* as a GA receptor in regulating plant height, and flower development^57–59^. The associations among the expressed *ZmSOC1*, the upregulated expression of *FUL* gene, the downregulated *GID1B*, the promoted flowering, and reduced plant height suggested that the GA signaling pathway (e.g.,* GID1B*) had involved in *ZmSOC1* expression-induced flowering and plant dwarfing. This provides a new insight to elucidate the pathway of an overexpressed *SOC1* in reducing plant height.

In addition, five DEGs (i.e., HSF24, CHLD, WRK70, TMK1, and PER31) involved in plant photosynthesis, phytohormone signaling, or abiotic tolerance (Table 2, Fig. 6) had impact on yield potential in the transgenic plants, and more studies are still needed to investigate photosynthetic efficiency, phytohormone types and contents, and abiotic tolerance.

## Conclusion

Soybean is a photoperiod-sensitive crop. Both plant height and days to flowering and maturity are of importance for soybean adaptability and yield. We demonstrated that expression of the *ZmSOC1* in soybean was very effective in promoting flowering and reducing plant height. Remarkably, the expression of the *ZmSOC1* could increase grain production. Transcriptome analysis of two transgenic lines revealed the genes underpinning the potential phenotypic changes driven by the expression of the *ZmSOC1* in the transgenic plants. Overall, the results provided new information to understand *SOC1*-mediated flowering in soybean. Most importantly, both the phenotypic and transcriptome data of the transgenic plants suggest that modulating expression of a MADS-box gene (e.g., *ZmSOC1*) is a powerful approach to hasten plant development and thus provides a great potential to enhance plant adaptability and yield.

## Supplementary Information

Supplementary Information.

## Data Availability

The datasets analyzed during the current study are available from the corresponding author on request.
