# Supplementary material for: Expression of a maize SOC1 gene enhances soybean yield potential through modulating plant growth and flowering
Source: Sci Rep. 2021 Jun 17;11:12758. doi: 10.1038/s41598-021-92215-x (PMC8211702; doi:10.1038/s41598-021-92215-x)
Supplement: Supplementary file 1 — Supplementary Information. [file 41598_2021_92215_MOESM1_ESM.pdf]

Expression of a maize *SOC1* gene enhances soybean yield potential through modulating plant growth and flowering

Xue Han[1], Dechun Wang[2], and Guo-qing Song[1]*

Fig. S1

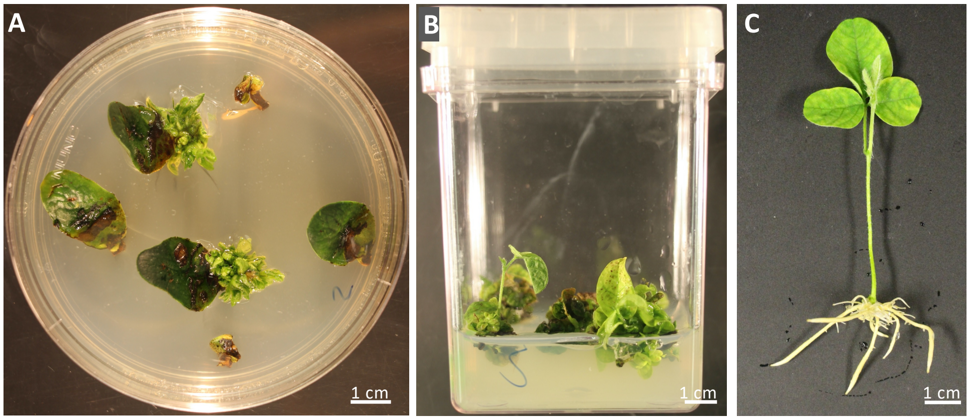

Fig. S1 *Agrobacterium*-tumefaciens mediated transformation and regeneration of herbicide resistant, *ZmSOC1*-containing soybean plants cv. Jack. (A) Herbicide resistant shoots induced from half-seed explants after 3-week selection. (B) Elongated shoots after 8 weeks. (B) A rooted plant produced after 4 weeks on the rooting medium.

Fig. S2

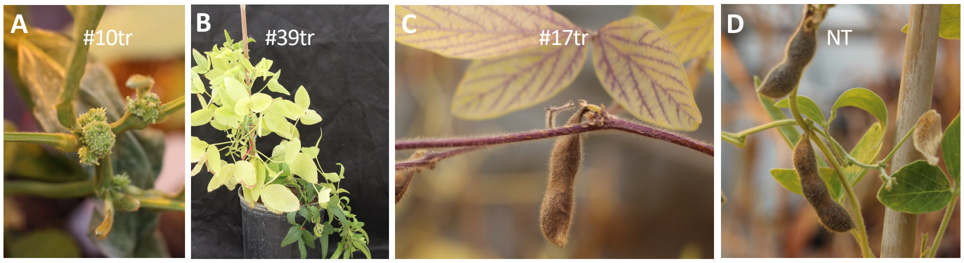

Fig. S2 Additional phenotypic variations observed in the T_0 transgenic plants grown in the greenhouse. (A) Altered flowers without formation of seed pods. (B) A chimeric plant in which the green part was nontransgenic (NT) and the yellow part was transgenic. (C) A transgenic plant showed purple stem and leaf color in contrast to a nontransgenic plant (D).

Fig. S3

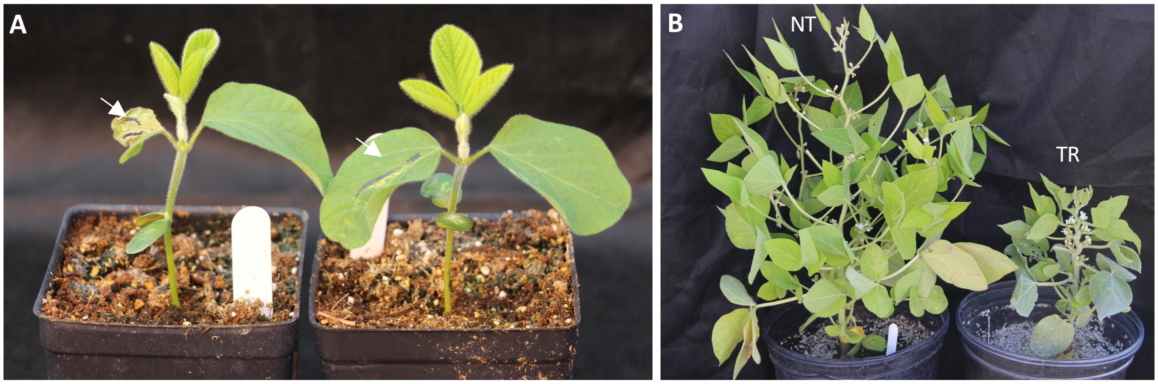

Fig. S3 Phenotypic variations in the T_1 transgenic plants. (A) Response of nontransgenic (NT) and transgenic (TR) seedlings to 150 mg/L leaf-painted herbicide after one week. Arrows show the painted leaves. (B) A normal growing NT plant and a dwarf transgenic plant.

Fig. S4

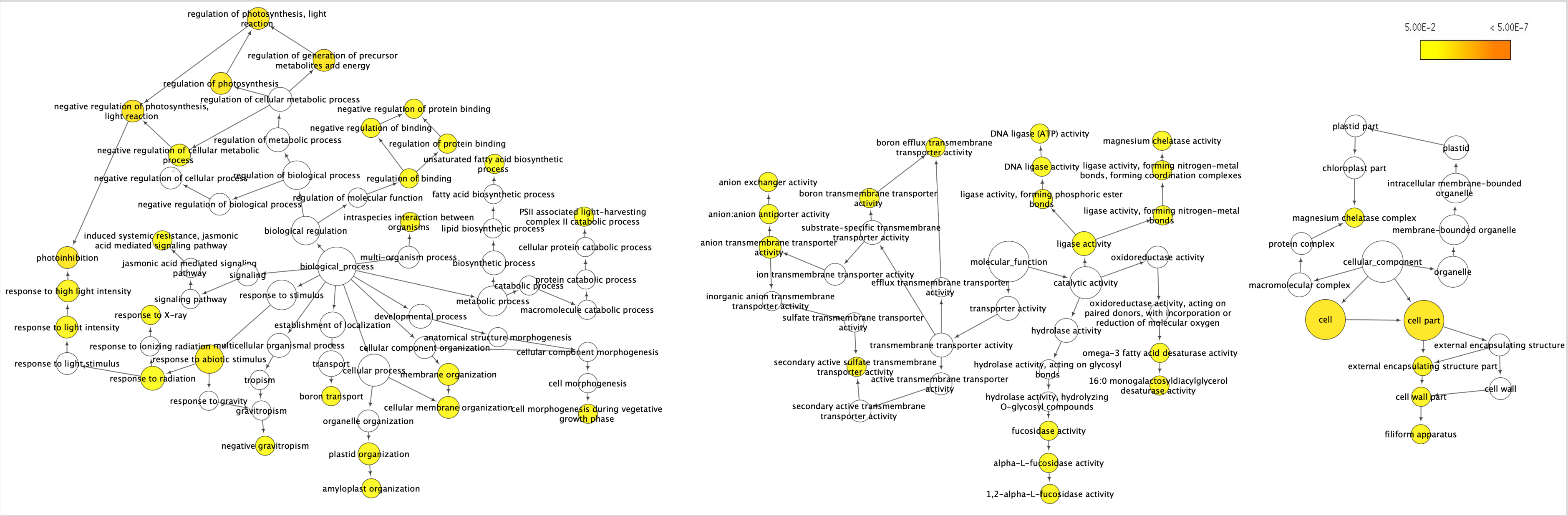

Fig. S4 Gene networks of differentially expressed transcripts in leaf tissues of transgenic ZmSOC1_OX plants. The ontology file of GOSlim_Plants in BiNGO was used to identify overrepresented GO terms (*p*< 0.05). Bubble size and color indicate the frequency of the GO term and the P-value, respectively.

Table S1 PCR primers

Primer name	Primer sequence (5' to 3')	Isoform ID
MK_R	GCCTGACCTGACCGCCACTGC	
MK_F	ATGGTGCGGGGCAAGACGCAG	
35S_F	TGA CGC ACA ATC CCA CTA TC	
BAR_R	ATG AGC CCA GAA CGA CGC C	
BAR_F	CAG ATC TCG GTG ACG G	
GmAct11_F	CGGTGGTTCTATCTTGGCATC	
GmAct11_R	GTCTTTCGCTTCAATAACCCTA	
gmActin1_F	GAG AGC CAA CAA GAG CAA TAG A	DN16267_c0_g2_i11
gmActin1_R	ACT GTT CCC AAT CCT CAC AAG	
ALFC5_F	TAC CCG CCG AAC ATT CTT TAG	DN17886_c0_g1_i15
ALFC5_R	GCT CTT CCG ATC TGT CCA TTT	
Ads3_F	GAG ATT CGT GAC CGT GAC TG	DN16057_c0_g1_i3
Ads3_R	GAG AGC CAT GAA GGG AAT TGA	
zmSOC1_F	GAC GGA GCT GTA CAT AGG ATT G	DN7082_c0_g1_i1
zmSOC1_R	GGC GTC TCG CAT ATC TCA TT	
pup18_F	TAG CCT CAA TTC CCA ACT ACA C	DN16521_c0_g2_i3
pup18_R	AAG CTG AAG GAG GAT GGT TAT C	
APRR3_F	CCT TGC AGC CTC TGA TAC AA	DN15112_c1_g2_i5
APRR3_R	AAA CAG AGG GTA GGC GAA TTT	
PER31_F	TGC TCT TCC GAT CTC TTC AAA C	DN14171_c0_g5_i1
PER31_R	CCT TGT AGA AGT CCA AGG TGA G	
SUT31_F	ACA CCA ACA CCT CCT TAA TAT CC	DN22021_c0_g1_i7
SUT31_R	CTA AAG GGT ACT CAT AAT CTA CAC TCC	
BOR4_F	TTT AGT CCT CTC CTC GTA TCC A	DN15732_c0_g1_i4
BOR4_R	GCT CTT CCT CGA AGA TCG TTT A	
qBar_F	TCA GCA GGT GGG TGT AGA	DN16691_c0_g6_i5
qBar_R	CAA CGC CTA CGA CTG GAC	

Table S2 Summary of transformation of soybean cultivar Jack

Experiment	No. of explants	No. of shoots produced in 4 weeks after co-cultivation	No. of elongated shoots produced in 6-14 weeks after co-cultivation	No. of rooted shoots in 2 weeks after the elongated shoots were transferred to rooting medium	No. of PCR positive T_0 lines	Transformation frequency (%)
Exp .1	381	260	36	26	15	3.9
Exp. 2	389	263	55	30	23	5.9

Table S3 Phenotypes of different T_0 lines growing in a greenhouse. Red numbers represent the lines that their T_1 plants were evaluated in the experiment 1. Bold numbers represent the lines that their T_1 plants were evaluated in the experiment 2.

Phenotype	No. of T_0 line	ID	PCR positive (P) or negative (N) lines	Days from planting to the appearance of the first flower	Days from planting to the appearance of the first pod
Dead	2	7, 8	P		
Dwarf and early flowering	1	39	P	0	27
Multiple branches	7	12, **21**, 22, **28**, 30, **31**, **35**	P	55	82
Increased number of branches, abnormal flowers, no pod	1	9	P	56	88
Increased number of branches, changed flower color (white to pink)	1	17	P	55	83
Increased number of branch and seed pods	4	**14**, **20**, **26**, 51	P	53	76
Increased number of branches and seed pods, later pod set	1	**4**	P	50	86
Abnormal flowers	2	19, 50	P	54	78
No branches, dwarf, abnormal flowers, sterile	1	15	P	51	75
No branches, dwarf, abnormal flowers, increased number of seed pods, sterile	1	2	P	51	86
No branches, abnormal flowers, sterile	1	10	P	14	52
No branches, increased number of seed pods	2	**16**, **25**	P	55	81
In vitro flowering and pod setting *in vitro*	2	44, **60**	P	0	27
No branches	1	11	N	56	78
No branches, similar to nontransgenic plants	11	18, 23, 24, 27, 29, 32, 33, **34**, 36, 37, 43	P	58	84

Table S4 PCR detection of transgenes in T_1 seedlings. Two pair of primers were used for amplifying the *bar* gene and the ZmSOC1, respectively. Red numbers represent the lines that their T_1 plants were evaluated in the experiment 1. Bold numbers represent the lines that their T plants were evaluated in the experiment 2.

ID	No. of seeds	No. of seeds germinated	No. of PCR positive seedlings	Ratio of transgenic/nontransgenic
#10	3	3	0	0.00
**#14**	30	23	6	0.35
**#16**	40	33	18	1.20
#17	20	20	0	0.00
#2	10	2	0	0.00
**#20**	40	39	29	2.90
#21	40	35	14	0.67
#22	30	21	6	0.40
#23	1	1	1	
**#25**	20	14	2	0.17
**#26**	28	27	1	0.04
**#28**	28	23	1	0.05
**#31**	20	19	0	0.00
**#34**	10	10	1	0.11
#35	30	26	11	0.73
#36	40	39	0	0.00
#38	40	34	2	0.06
#39	3	3	0	0.00
**#4**	40	16	2	0.14
**#60**	20	17	12	2.40
#9	20	19	0	0.00

Table S5 Phenotypic data of in T1 transgenic (TR) soybean plants expressing the ZmSOC1 in the two experiments, where the seeds were sowed on May 4th in the experiment1 and June 4th in the [illegible]

	id1	id2	line	genotype	Node_numb	flower_time (days)	pod_set (days)	Height (cm)	Branch_number	Node_number	Pod_number	Seed_dry_weight (g)	100_seeds_dry_weight
exp1	14#57	nt	14#	nt	6	36	98	82	12	28	140	37.5	8.85
exp1	14#68	nt	14#	nt	6	40	109	91	11	27	136	16.3	9.8
exp1	14#69	nt	14#	nt	5	36	114	72	8	26	129	15.8	
exp1	14#74	nt	14#	nt	6	38	111	83	9	28	139	13.9	
exp1	2#3	nt	2#	nt	7	45	104	81	8	30	146	15.6	11.3
exp1	2#4	nt	2#	nt	6	42	104	79	6	29	152	21.3	9.6
exp1	22#31	nt	22#	nt	6	38	94	81	10	27	106	27	10.6
exp1	35#66	nt	35#	nt	6	43	116	80	9	29	117	15.2	7.6
exp1	35#70	nt	35#	nt	5	45	113	87	10	27	127	27.1	7.05
exp1	4#82	nt	4#	nt	6	39	93	80	9	29	131	20.5	
exp1	4#84	nt	4#	nt	6	36	101	83	10	27	152	13	8
exp1	9#89	nt	9#	nt	6	40	118	85	10	27	132	22.3	
exp1	17#88	nt	17#	nt	7	45	89	72	8	29	152	15.3	6.65
exp1	9#91	nt	9#	nt	6	43	116	80	9	28	129	18.2	8.55
exp1	Jack11	nt	Jack1	nt	7	43		88	10	29	145		
exp1	Jack22	nt	Jack2	nt	6	43		80	8	28	132		
exp1	14#46	14#tr	14#	tr	5	31	93	75	9	31	136	19.4	8.65
exp1	14#47	14#tr	14#	tr	5	38	99	68	9	29	158	12.1	7.2
exp1	14#54	14#tr	14#	tr	3	31	99	89	10	33	205	30.2	8.25
exp1	14#56	14#tr	14#	tr	4	33	100	81	9	31	191	32.8	10.05
exp1	14#67	14#tr	14#	tr	4	30	53	77	10	31	172	34	10.2
exp1	14#73	14#tr	14#	tr	5	36	52	88	11	31	162	14.7	6.6
exp1	16#28	16#tr	16#	tr	5	38	64	95	9	34	212	42.6	12.8
exp1	16#38	16#tr	16#	tr	4	36	83	65	6	29	164	35.9	18.2
exp1	16#40	16#tr	16#	tr	4	33	59	52	4	22	132	30.4	11.25
exp1	16#43	16#tr	16#	tr	4	30	99	78	4	26	156	15.7	7
exp1	16#51	16#tr	16#	tr	4	29	89	75	9	26	189	41.2	11.3
exp1	16#52	16#tr	16#	tr	6	38	70	72	8	26	189	22.3	6.4
exp1	16#53	16#tr	16#	tr	4	33	113	75	9	29	156	22.4	9.65
exp1	16#58	16#tr	16#	tr	4	31	86	83	5	31	149	27.8	9.65
exp1	16#61	16#tr	16#	tr	4	30	59	58	4	22	163	10.8	6.55
exp1	16#62	16#tr	16#	tr	4	30	79	77	5	31	189	32.4	9.95
exp1	20#35	20#tr	20#	tr	3	30	45	81	6	29	182	22.4	8.55
exp1	20#36	20#tr	20#	tr	4	29	48	54	4	21	156	18.6	10.2
exp1	20#37	20#tr	20#	tr	4	33	83	68	5	30	196	33.6	11.55
exp1	20#39	20#tr	20#	tr	4	33	101	70	6	29	158	17.8	8.75
exp1	20#41	20#tr	20#	tr	4	29	45	57	4	25	116	21.5	8.95
exp1	20#42	20#tr	20#	tr	4	33	80	88	7	31	163	18	9.4

	id1	id2	line	genotype	Node_numb	flower_time (days)	pod_set (days)	Height (cm)	Branch_number	Node_number	Pod_number	Seed_dry_weight (g)	100_seeds_dry_weight
exp1	20#44	20#tr	20#	tr	3	33	101	86	9	33	172	27.4	7.05
exp1	20#45	20#tr	20#	tr	4	30	104	72	7	31	161	30.5	9.75
exp1	20#48	20#tr	20#	tr	3	29	44	70	8	28	142	20.3	8.9
exp1	20#49	20#tr	20#	tr	2	24	38	42	3	18	88	17.2	7.8
exp1	20#75	20#tr	20#	tr	5	33	73	69	8	26	109	18.6	6.6
exp1	20#76	20#tr	20#	tr	4	31	109	71	6	28	143	15.1	7.3
exp1	20#77	20#tr	20#	tr	3	29	45	81	3	28	150	28.6	
exp1	20#78	20#tr	20#	tr	6	43	88	70	7	33	167	38.4	10.8
exp1	20#79	20#tr	20#	tr	3	29	45	73	10	27	214	36.7	10.6
exp1	20#80	20#tr	20#	tr	4	31	58	58	4	21	132	29.3	11.75
exp1	21#81	21#tr	21#	tr	3	33	99	71	9	34	217	18.8	8.3
exp1	21#83	21#tr	21#	tr	4	36	65	75	4	26	129	18.9	9
exp1	21#85	21#tr	21#	tr	5	33	113	80	10	29	138	15.6	8.15
exp1	21#16	21#tr	21#	tr	5	38	94	82	10	29	141	24.6	10
exp1	22#32	22#tr	22#	tr	5	36	109	79	9	30	131	15.4	8.5
exp1	22#33	22#tr	22#	tr	7	40	118	74	8	28	136	24.4	8.4
exp1	22#34	22#tr	22#	tr	4	36	75	60	6	25	99	19.2	9.6
exp1	23#50	23#tr	23#	tr	3	29	80	88	7	33	173	20.1	8.05
exp1	25#92	25#tr	25#	tr	3	31	60	76	6	30	152	33.4	10.75
exp1	26#96	26#tr	26#	tr	5	40	113	78	10	29	128	19.4	10
exp1	35#55	35#tr	35#	tr	4	30	45	78	4	27	136	16.4	8.05
exp1	35#59	35#tr	35#	tr	3	30	90	75	10	26	238	37.1	9.05
exp1	35#63	35#tr	35#	tr	3	36	114	71	8	26	132	14.5	7.25
exp1	35#65	35#tr	35#	tr	4	33	113	89	10	29	122	27	8.6
exp1	35#71	35#tr	35#	tr	6	43	104	62	5	21	116	25.9	10.75
exp1	35#72	35#tr	35#	tr	6	45	104	74	7	26	149	33.6	10.25
exp1	35#14	35#tr	35#	tr	4	33	53	91	10	33	224	29.8	9.8
exp1	35#64	35#tr	35#	tr	4	36	113	69	4	26	93	18.6	6.6
exp1	4#86	4#tr	4#	tr	5	38	111	80	10	27	132	22.1	9.65
exp1	4#87	4#tr	4#	tr	5	36	111	66	6	28	142	23.5	7.15
exp2	34#1	nt	34#	nt	7	48	67	45	3	16	71	10.1	7.25
exp2	34#3	nt	34#	nt	7	48	68	47	4	17	68	12	8.5
exp2	34#4	nt	34#	nt	7	51	69	43	4	17	58	17.3	9.65
exp2	34#5	nt	34#	nt	7	51	67	47	4	18	102	12	8.5
exp2	14#1	nt	14#	nt	7	56	70	42	5	16	62	15.2	8.6
exp2	14#3	nt	14#	nt	6	47	65	53	4	17	71	21	12.55
exp2	28#1	nt	28#	nt	6	45	65	42	4	16	93	22	9.65
exp2	28#2	nt	28#	nt	7	48	67	44	4	17	72	18.4	9.9
exp2	28#3	nt	28#	nt	6	45	65	42	3	15	68	22	9.65
exp2	28#5	nt	28#	nt	6	45	81	49	4	17	99	28.6	8.4
exp2	16#4	nt	16#	nt	7	46	61	44	5	16	76	15.6	9.05

	id1	id2	line	genotype	Node_numb	flower_time (days)	pod_set (days)	Height (cm)	Branch_number	Node_number	Pod_number	Seed_dry_weight (g)	100_seeds_dry_weight
exp2	21#5	nt	21#	nt	6	45	62	50	6	19	99	20.1	7.65
exp2	26#1	nt	26#	nt	7	46	70	48	4	18	81	19.9	11.4
exp2	26#2	nt	26#	nt	7	50	70	51	4	18	88	18.8	9.25
exp2	26#3	nt	26#	nt	7	52	75	58	3	18	90	17.6	10.5
exp2	4#1	nt	4#	nt	7	51	75	52	4	17	52	16.3	12.36
exp2	4#2	nt	4#	nt	7	58	78	43	4	15	74	15.1	11.75
exp2	4#3	nt	4#	nt	7	52	73	53	5	18	119	13	11.68
exp2	4#4	nt	4#	nt	7	50	74	50	4	18	96	15.2	12.38
exp2	31#1	nt	31#	nt	7	50	69	48	4	16	91	25.6	9.5
exp2	31#2	nt	31#	nt	7	46	68	49	4	17	81	24.1	12.5
exp2	31#3	nt	31#	nt	7	51	77	51	4	19	114	25.3	11.3
exp2	31#4	nt	31#	nt	6	50	77	48	6	17	87	25.6	9.5
exp2	31#5	nt	31#	nt	6	46	67	47	6	18	74	25.9	14.5
exp2	31#6	nt	31#	nt	7	48	69	51	5	19	112	25.3	11.3
exp2	31#7	nt	31#	nt	7	47	69	45	4	15	69	24.1	11.9
exp2	31#8	nt	31#	nt	6	49	70	47	4	16	44	25.9	14.5
exp2	31#9	nt	31#	nt	6	48	68	52	4	19	80	14.4	10.8
exp2	31#10	nt	31#	nt	7	51	70	49	4	18	53	24.1	12.5
exp2	WT11	nt	WT1	nt	7	48	69	58	6	19	90	17.5	10
exp2	WT22	nt	WT2	nt	7	48	68	52	7	18	68	16.7	
exp2	WT33	nt	WT3	nt	7	48	72	53	6	18	72	18.3	
exp2	34#2	34#tr	34#	tr	7	51	68	40	3	15	62	12.3	5.5
exp2	35#1	35#tr	35#	tr	6	46	58	39	6	18	114	33.4	9.9
exp2	35#2	35#tr	35#	tr	6	50	60	41	4	17	91	29.6	10.55
exp2	38#1	38#tr	38#	tr	5	44	62	40	2	15	72	22.8	8.85
exp2	38#2	38#tr	38#	tr	4	42	59	40	4	16	81	22.8	8.85
exp2	60#1	60#tr	60#	tr	2	27	56	44	6	21	128	15.4	9.8
exp2	60#2	60#tr	60#	tr	3	24	58	47	6	21	132	20.66	8.5
exp2	60#3	60#tr	60#	tr	2	27	53	43	5	20	108	21.6	9.25
exp2	60#4	60#tr	60#	tr	3	34	48	33	3	15	83	20.3	9.65
exp2	60#5	60#tr	60#	tr	2	34	53	32	3	15	68	22.3	8.1
exp2	60#6	60#tr	60#	tr	3	34	54	36	4	17	93	18.4	12.55
exp2	60#7	60#tr	60#	tr	3	43	58	37	4	16	92	21.7	7.45
exp2	14#2	14#tr	14#	tr	6	56	62	39	5	16	58	18.1	11
exp2	28#4	28#tr	28#	tr	4	46	59	45	4	18	102	30.7	8.9
exp2	25#1	25#tr	25#	tr	6	44	69	46	5	17	107	21.8	11.4
exp2	16#1	16#tr	16#	tr	5	41	62	35	5	15	82	13.3	7.9
exp2	16#2	16#tr	16#	tr	7	50	58	41	4	16	89	27.1	11.05
exp2	16#3	16#tr	16#	tr	5	44	54	37	4	17	78	26.9	13.5
exp2	16#5	16#tr	16#	tr	6	43	58	36	4	16	73	26.7	7.3
exp2	16#6	16#tr	16#	tr	6	44	61	38	4	15	82	24.7	12.75

	id1	id2	line	genotype	Node_numb	flower_time (days)	pod_set (days)	Height (cm)	Branch_number	Node_number	Pod_number	Seed_dry_weight (g)	100_seeds_dry_weight
exp2	16#7	16#tr	16#	tr	6	40	52	55	4	22	113	23.3	11.3
exp2	21#1	21#tr	21#	tr	6	48	77	33	4	15	60	15.5	9.55
exp2	21#2	21#tr	21#	tr	5	49	68	44	6	17	82	23.3	10.9
exp2	21#3	21#tr	21#	tr	7	51	67	48	8	19	98	21.1	13.3
exp2	21#4	21#tr	21#	tr	7	51	65	42	4	17	72	21.3	9.45
exp2	21#6	21#tr	21#	tr	6	44	59	36	6	17	68	31	7.1
exp2	21#7	21#tr	21#	tr	6	46	68	44	7	19	92	23.3	10.9
exp2	21#8	21#tr	21#	tr	4	48	67	44	4	18	93	23.3	10.9
exp2	21#9	21#tr	21#	tr	5	34	60	41	4	19	68	21.8	14.25
exp2	21#10	21#tr	21#	tr	6	45	60	42	4	17	71	21.3	9.45
exp2	21#11	21#tr	21#	tr	5	45	60	48	4	17	63	21.1	13.3
exp2	20#1	20#tr	20#	tr	2	27	42	36	4	16	74	21.5	15.3
exp2	20#2	20#tr	20#	tr	7	52	39	46	7	20	128	19.2	7.05
exp2	20#3	20#tr	20#	tr	3	28	48	30	4	14	53	18.1	6.5
exp2	20#4	20#tr	20#	tr	4	33	57	61	5	23	158	25.6	9.4
exp2	20#5	20#tr	20#	tr	5	32	60	57	4	21	139	37.8	11.85
exp2	20#6	20#tr	20#	tr	6	57	77	39	4	17	63	8.7	7.1
exp2	20#7	20#tr	20#	tr	3	27	45	30	3	17	52	15.4	7.8
exp2	20#8	20#tr	20#	tr	7	57	81	43	6	17	71	18.6	10.9
exp2	20#9	20#tr	20#	tr	7	51	79	51	6	20	110	14.5	9.56
exp2	20#10	20#tr	20#	tr	5	32	68	60	4	24	142	27.9	11.5
exp2	20#11	20#tr	20#	tr	4	36	68	48	3	20	86	19.2	12.34
exp2	20#12	20#tr	20#	tr	6	66	69	60	4	21	121	26.5	10.55
exp2	20#13	20#tr	20#	tr	4	67	79	31	0	21			

Table S6 Differentially expressed genes (DEGs) in two transgenic lines (#20tr and #60tr), each compared to the nontransgenic line. #N/As represent a non-DEGs.

Isoform ID	Log_2(#60tr/nt)	Log_2(#20tr/nt)	Log_2(#60tr/#20t	Annotation_protein	e_value_annotation
DN10333_c0_g1_i1	#N/A	-0.92	1.22	CXE18_ARATH	9.00E-138
DN11200_c0_g1_i1	#N/A	-1.04	#N/A	.	#N/A
DN11200_c0_g1_i2	#N/A	-1.2	#N/A	AHP1_ARATH	1.50E-68
DN11267_c0_g1_i2	#N/A	11.21	#N/A	YNXB_BACSU	0.042
DN11408_c0_g1_i2	#N/A	1.61	#N/A	CHIT5_LOTJA	0
DN11475_c0_g2_i1	#N/A	#N/A	3.87	LEC5_VIGUC	3.00E-119
DN11478_c0_g1_i2	#N/A	#N/A	0.66	.	#N/A
DN11515_c1_g1_i1	#N/A	7.53	-7.65	LYK3_MEDTR	0
DN11606_c0_g1_i1	#N/A	#N/A	0.79	.	#N/A
DN11625_c0_g1_i1	#N/A	#N/A	0.58	FKB13_ARATH	2.40E-76
DN11657_c0_g1_i1	#N/A	#N/A	0.55	BOLA4_ARATH	1.10E-48
DN11787_c0_g1_i1	#N/A	-0.95	#N/A	.	#N/A
DN11889_c0_g1_i2	#N/A	#N/A	0.81	SDH6_ARATH	4.40E-69
DN11928_c0_g1_i3	#N/A	#N/A	2.33	.	#N/A
DN11989_c0_g1_i1	#N/A	#N/A	6.45	.	#N/A
DN12053_c0_g2_i1	#N/A	#N/A	2.02	Y4276_ARATH	5.10E-06
DN12086_c0_g1_i1	#N/A	#N/A	1.81	ENDO2_ARATH	2.00E-116
DN12092_c0_g1_i2	#N/A	#N/A	-4.55	MIL1_YEAST	3
DN12201_c0_g1_i4	#N/A	#N/A	1.22	FDC1_ARATH	1.50E-40
DN12201_c0_g1_i7	#N/A	#N/A	0.71	FDC1_ARATH	5.20E-63
DN12305_c0_g1_i1	#N/A	#N/A	0.58	.	#N/A
DN12346_c0_g1_i6	#N/A	#N/A	1.33	SMR3_ARATH	1.30E-05
DN12358_c0_g1_i1	#N/A	#N/A	0.79	CRR3_ARATH	1.90E-31
DN12372_c0_g1_i2	#N/A	#N/A	0.72	LBP65_ARATH	5.10E-41
DN12456_c0_g1_i1	#N/A	#N/A	0.78	COA6_BOVIN	0.003
DN12497_c0_g1_i1	#N/A	#N/A	0.9	.	#N/A
DN12497_c0_g2_i2	#N/A	#N/A	0.68	.	#N/A
DN12515_c0_g1_i3	#N/A	#N/A	0.93	.	#N/A
DN12571_c0_g1_i7	#N/A	8.19	#N/A	NSTU1_ARATH	0
DN12665_c0_g2_i3	#N/A	2.64	#N/A	PANB_PROM2	1.8
DN12689_c0_g1_i1	#N/A	#N/A	1.81	.	#N/A
DN12689_c0_g1_i3	#N/A	#N/A	2.03	.	#N/A
DN12750_c0_g1_i3	#N/A	#N/A	0.65	MAS12_AGRRH	0.00015
DN12771_c0_g1_i2	#N/A	#N/A	0.6	.	#N/A
DN12771_c0_g2_i1	#N/A	#N/A	0.7	.	#N/A
DN12775_c0_g1_i1	#N/A	#N/A	1.04	EM514_ARATH	1.70E-53

Isoform ID	Log$_2$(#60tr/nt)	Log$_2$(#20tr/nt)	Log$_2$(#60tr/#20t	Annotation_protein	e_value_annotation
DN12775_c0_g1_i2	#N/A	#N/A	0.81	EM514_ARATH	2.60E-53
DN12870_c0_g1_i3	#N/A	#N/A	0.97	HDA8_ARATH	5.70E-51
DN12917_c0_g2_i1	#N/A	#N/A	0.85	RS92_ARATH	1.00E-124
DN12917_c0_g2_i2	#N/A	#N/A	0.76	RS92_ARATH	1.00E-124
DN12918_c0_g2_i2	#N/A	-1.06	#N/A	.	#N/A
DN12918_c0_g3_i1	#N/A	-0.66	#N/A	.	#N/A
DN12942_c0_g1_i5	#N/A	#N/A	8.68	.	#N/A
DN12944_c0_g1_i1	#N/A	#N/A	1.76	PLAO1_PLAOI	3.40E-26
DN12944_c0_g1_i3	#N/A	#N/A	1.69	PLAO1_PLAOI	2.30E-26
DN12970_c0_g1_i2	#N/A	-1.9	#N/A	FLHA_BUCAP	1.4
DN12982_c0_g1_i5	#N/A	#N/A	0.81	CH101_ARATH	1.90E-60
DN12982_c0_g1_i6	#N/A	#N/A	0.92	CH101_ARATH	4.60E-59
DN13010_c0_g1_i1	#N/A	#N/A	0.62	RK22_MEDSA	2.00E-75
DN13010_c0_g1_i2	#N/A	#N/A	0.82	RK22_PEA	6.10E-71
DN13013_c1_g1_i1	#N/A	3.32	#N/A	ALPL_ARATH	5.20E-38
DN13020_c0_g1_i2	#N/A	#N/A	0.69	.	#N/A
DN13041_c0_g1_i7	#N/A	7.39	#N/A	.	#N/A
DN13050_c0_g1_i1	#N/A	#N/A	0.73	.	#N/A
DN13050_c0_g1_i3	#N/A	#N/A	0.72	.	#N/A
DN13052_c0_g1_i4	#N/A	#N/A	1.85	RLA2B_MAIZE	2.10E-30
DN13052_c0_g1_i5	#N/A	#N/A	1.1	RLA24_ARATH	2.30E-31
DN13071_c0_g1_i3	#N/A	#N/A	2.04	MYB14_ARATH	5.20E-55
DN13111_c0_g1_i6	#N/A	#N/A	0.74	.	#N/A
DN13119_c0_g1_i1	#N/A	#N/A	0.58	.	#N/A
DN13128_c0_g1_i5	#N/A	#N/A	0.63	.	#N/A
DN13153_c0_g1_i4	#N/A	#N/A	8.26	XTH1_ARATH	1.30E-37
DN13169_c0_g1_i3	#N/A	#N/A	1.32	NDHS_ARATH	1.90E-32
DN13203_c0_g1_i4	#N/A	#N/A	1.28	QCR72_ARATH	2.00E-69
DN13204_c0_g1_i3	#N/A	#N/A	1.15	RL23A_DAUCA	1.60E-83
DN13205_c0_g1_i1	#N/A	#N/A	0.91	.	#N/A
DN13205_c0_g2_i1	#N/A	#N/A	1.17	.	#N/A
DN13267_c3_g1_i1	#N/A	-0.72	#N/A	.	#N/A
DN13267_c3_g1_i3	#N/A	-0.72	#N/A	.	#N/A
DN13270_c0_g1_i5	#N/A	#N/A	0.62	RR10_SPIOL	3.00E-81
DN13326_c0_g1_i2	#N/A	#N/A	1.13	DYL1_DEBHA	6.40E-20
DN13333_c0_g1_i3	#N/A	#N/A	1.24	.	#N/A
DN13333_c0_g1_i4	#N/A	#N/A	0.94	PSBW_SPIOL	2.10E-41
DN13333_c0_g1_i5	#N/A	#N/A	0.7	PSBW_SPIOL	9.80E-54
DN13333_c0_g1_i6	#N/A	#N/A	0.77	PSBW_SPIOL	4.60E-49

Isoform ID	Log_2(#60tr/nt)	Log_2(#20tr/nt)	Log_2(#60tr/#20t	Annotation_protein	e_value_annotation
DN13334_c0_g1_i4	#N/A	#N/A	1.67	.	#N/A
DN13334_c0_g1_i5	#N/A	#N/A	1.03	RS12_HORVU	2.30E-71
DN13334_c0_g1_i7	#N/A	#N/A	1.04	RS12_HORVU	2.40E-72
DN13334_c0_g1_i9	#N/A	#N/A	0.78	RS12_HORVU	2.40E-72
DN13345_c0_g1_i4	#N/A	#N/A	0.86	ENO_HUNT2	4.1
DN13379_c0_g1_i8	#N/A	#N/A	0.66	TCLOT_ARATH	1.30E-53
DN13386_c0_g1_i1	#N/A	#N/A	1.09	.	#N/A
DN13386_c0_g1_i5	#N/A	#N/A	0.59	ADA29_HUMAN	0.54
DN13393_c1_g1_i6	#N/A	#N/A	0.64	.	#N/A
DN13394_c0_g1_i7	#N/A	#N/A	0.9	MUTS2_AQUAE	1.2
DN13413_c0_g2_i3	#N/A	#N/A	1	RL23_ARATH	4.20E-97
DN13413_c0_g2_i5	#N/A	#N/A	1	RL23_ARATH	3.40E-97
DN13417_c0_g1_i6	#N/A	#N/A	0.91	.	#N/A
DN13441_c0_g2_i5	#N/A	-0.84	#N/A	.	#N/A
DN13441_c0_g2_i6	#N/A	-0.81	#N/A	.	#N/A
DN13478_c0_g1_i2	#N/A	3.13	#N/A	K0930_RAT	0.42
DN13491_c0_g1_i5	#N/A	#N/A	0.69	GSTX3_SOYBN	1.00E-127
DN13512_c0_g1_i4	#N/A	#N/A	1.39	RDRP_OLV2I	5.7
DN13520_c0_g1_i3	#N/A	#N/A	1.72	.	#N/A
DN13521_c0_g1_i3	#N/A	#N/A	0.95	.	#N/A
DN13521_c0_g1_i5	#N/A	#N/A	0.99	.	#N/A
DN13531_c0_g2_i7	#N/A	4.26	#N/A	Y1648_ARATH	0.15
DN13543_c0_g1_i3	#N/A	#N/A	0.75	.	#N/A
DN13543_c0_g1_i5	#N/A	#N/A	0.8	.	#N/A
DN13552_c0_g1_i3	#N/A	#N/A	-1.22	PHOT1_ARATH	1.40E-98
DN13552_c0_g1_i6	#N/A	#N/A	-1.47	PHOT1_ARATH	4.00E-133
DN13555_c0_g2_i6	#N/A	#N/A	0.97	.	#N/A
DN13555_c0_g2_i7	#N/A	#N/A	0.81	.	#N/A
DN13555_c0_g4_i2	#N/A	#N/A	0.83	.	#N/A
DN13555_c0_g4_i3	#N/A	#N/A	1.07	G6PD_MEDSA	1.1
DN13568_c0_g1_i3	#N/A	#N/A	0.69	TPPC3_CHICK	2.80E-70
DN13568_c0_g1_i5	#N/A	#N/A	0.77	TPPC3_CHICK	2.80E-70
DN13579_c1_g3_i3	#N/A	#N/A	5.78	CPP1_ARATH	4.40E-79
DN13612_c0_g1_i10	#N/A	#N/A	0.9	RL40_NICSY	1.20E-91
DN13612_c0_g1_i9	#N/A	#N/A	1.1	RL40_NICSY	2.10E-91
DN13615_c0_g2_i1	#N/A	#N/A	0.64	Y3250_JANMA	0.002
DN13649_c0_g1_i1	#N/A	#N/A	0.84	LTD_ARATH	1.20E-74
DN13649_c0_g1_i2	#N/A	#N/A	0.65	LTD_ARATH	9.30E-75
DN13652_c0_g3_i3	#N/A	-0.7	#N/A	C7317_PRUMU	0

Isoform ID	Log_2(#60tr/nt)	Log_2(#20tr/nt)	Log_2(#60tr/#20t	Annotation_protein	e_value_annotation
DN13671_c0_g4_i7	#N/A	#N/A	0.62	TRNHF_ARATH	4.00E-132
DN13675_c0_g5_i3	#N/A	#N/A	0.87	H2AX_CICAR	2.40E-90
DN13675_c0_g6_i1	#N/A	#N/A	0.96	RL31_PERFR	2.30E-69
DN13675_c0_g6_i5	#N/A	#N/A	0.97	RL31_PERFR	1.70E-68
DN13690_c0_g1_i6	#N/A	#N/A	1.24	IMP3_MOUSE	4.40E-72
DN13693_c0_g1_i5	#N/A	-0.73	#N/A	CXE18_ARATH	6.00E-147
DN13703_c2_g2_i1	#N/A	#N/A	-0.84	CLCE_ARATH	2.40E-87
DN13719_c0_g1_i1	#N/A	#N/A	0.66	RL27_PEA	1.60E-88
DN13719_c0_g1_i2	#N/A	#N/A	0.96	RL27_PEA	8.10E-90
DN13719_c0_g1_i5	#N/A	#N/A	0.95	.	#N/A
DN13719_c0_g2_i1	#N/A	#N/A	1.33	.	#N/A
DN13727_c0_g1_i1	#N/A	#N/A	1.12	CB5_ARATH	2.90E-48
DN13736_c0_g1_i1	#N/A	#N/A	0.73	PLAS_PEA	1.70E-86
DN13736_c0_g1_i2	#N/A	#N/A	0.64	PLAS_PEA	1.70E-83
DN13742_c0_g1_i3	#N/A	-1.8	#N/A	C93C1_SOYBN	0
DN13762_c0_g1_i7	#N/A	#N/A	8.76	Y4845_ARATH	3.20E-92
DN13773_c0_g2_i1	#N/A	#N/A	0.61	ADT3_ARATH	1.80E-63
DN13773_c0_g5_i7	#N/A	#N/A	-7.96	ADT1_SOLTU	0
DN13779_c0_g1_i1	#N/A	#N/A	0.66	.	#N/A
DN13783_c0_g1_i1	#N/A	#N/A	0.69	CUBN_MOUSE	2
DN13819_c0_g3_i4	#N/A	-0.83	0.9	EI3G2_DROVI	1.9
DN13828_c0_g1_i2	#N/A	#N/A	0.68	RL34_PEA	4.00E-78
DN13835_c2_g2_i2	#N/A	#N/A	1.79	PORA_CUCSA	1.00E-127
DN13835_c2_g2_i7	#N/A	#N/A	3.94	.	#N/A
DN13835_c2_g2_i9	#N/A	#N/A	2.28	PORA_CUCSA	0
DN13861_c0_g1_i12	#N/A	#N/A	3.89	E70H1_ARATH	9.20E-84
DN13871_c0_g2_i34	#N/A	7.58	#N/A	.	#N/A
DN13873_c0_g1_i4	#N/A	-0.76	#N/A	SYC_PROMM	2
DN13885_c0_g2_i4	#N/A	#N/A	0.97	.	#N/A
DN13885_c0_g3_i2	#N/A	#N/A	1.13	RS24_ARATH	3.00E-151
DN13889_c0_g1_i6	#N/A	8.39	#N/A	MBF1A_ARATH	4.90E-86
DN13891_c0_g1_i2	#N/A	#N/A	0.94	.	#N/A
DN13900_c0_g1_i1	#N/A	#N/A	1.21	.	#N/A
DN13900_c0_g1_i5	#N/A	#N/A	1.19	.	#N/A
DN13900_c0_g4_i1	#N/A	#N/A	1.19	.	#N/A
DN13900_c0_g4_i3	#N/A	#N/A	1.27	.	#N/A
DN13902_c2_g1_i7	#N/A	#N/A	-4.85	EFCB6_HUMAN	1.8
DN13907_c0_g1_i2	#N/A	#N/A	1.71	.	#N/A
DN13911_c0_g4_i3	#N/A	#N/A	-1.28	DXR_MENPI	0

Isoform ID	Log_2(#60tr/nt)	Log_2(#20tr/nt)	Log_2(#60tr/#20t	Annotation_protein	e_value_annotation
DN13953_c0_g2_i5	#N/A	#N/A	0.91	HD3B_ORYSJ	0.91
DN13960_c1_g1_i2	#N/A	#N/A	0.61	NDADB_ARATH	8.00E-91
DN13960_c1_g1_i9	#N/A	#N/A	0.6	NDADB_ARATH	2.80E-90
DN13962_c0_g1_i10	#N/A	#N/A	8.27	ACBP3_ARATH	1.40E-30
DN13963_c0_g1_i14	#N/A	#N/A	0.82	YLMG2_ARATH	1.20E-70
DN13966_c0_g1_i1	#N/A	#N/A	0.68	NDK1_SOYBN	9.00E-106
DN13966_c0_g1_i2	#N/A	#N/A	0.69	NDK1_SOYBN	1.00E-104
DN13981_c0_g4_i1	#N/A	#N/A	0.99	.	#N/A
DN13982_c0_g2_i15	#N/A	#N/A	-1.03	ROQ1_NICBE	2.00E-159
DN14005_c0_g1_i6	#N/A	#N/A	-7.99	.	#N/A
DN14009_c0_g2_i2	#N/A	#N/A	0.77	RL40_NICSY	2.10E-91
DN14009_c0_g2_i7	#N/A	#N/A	1.02	RL40_NICSY	2.10E-91
DN14009_c0_g5_i1	#N/A	#N/A	1.04	.	#N/A
DN14009_c0_g5_i3	#N/A	#N/A	0.85	.	#N/A
DN14013_c0_g4_i1	#N/A	#N/A	0.72	MWL1_ARATH	0.75
DN14029_c2_g1_i9	#N/A	#N/A	1.52	TAUE3_ARATH	2.00E-104
DN14040_c0_g1_i11	#N/A	#N/A	1.32	ICE1_ARATH	3.00E-115
DN14046_c0_g1_i1	#N/A	5.15	#N/A	BLML2_DICDI	0.03
DN14049_c0_g1_i1	#N/A	#N/A	0.85	ZFP4_ARATH	3.30E-38
DN14052_c0_g1_i22	#N/A	#N/A	0.69	TI223_ARATH	1.20E-80
DN14058_c0_g1_i4	#N/A	8.03	#N/A	RL13_GEMAT	7.60E-40
DN14064_c0_g1_i1	#N/A	#N/A	0.72	GCSH_FLAAN	2.00E-102
DN14064_c0_g1_i3	#N/A	#N/A	-2.39	GCSH_FLAAN	2.00E-102
DN14064_c0_g1_i6	#N/A	#N/A	0.68	GCSH_FLAAN	6.00E-101
DN14074_c0_g2_i4	#N/A	#N/A	9.73	DRP1E_ARATH	2.50E-63
DN14078_c0_g1_i8	#N/A	#N/A	0.91	RL9_PEA	2.00E-123
DN14078_c0_g1_i9	#N/A	#N/A	1	RL9_PEA	2.00E-123
DN14078_c0_g2_i4	#N/A	#N/A	0.89	RL9_PEA	9.00E-127
DN14078_c0_g2_i5	#N/A	#N/A	0.93	RL9_PEA	1.00E-127
DN14086_c0_g4_i1	#N/A	9.93	-10.06	TRXX_ARATH	4.20E-60
DN14087_c0_g2_i8	#N/A	-0.64	#N/A	.	#N/A
DN14095_c0_g3_i5	#N/A	#N/A	0.91	HDT1_SOYBN	1.00E-171
DN14113_c0_g1_i7	#N/A	#N/A	0.74	NDUS8_SOLTU	2.00E-143
DN14116_c0_g1_i7	#N/A	#N/A	-1.05	DRL42_ARATH	6.80E-30
DN14119_c1_g1_i2	#N/A	-0.77	#N/A	CDF3_ARATH	1.00E-74
DN14135_c1_g4_i5	#N/A	#N/A	-0.83	CLPC_PEA	8.00E-106
DN14140_c0_g1_i11	#N/A	#N/A	-7.37	HAT5_ARATH	3.60E-40
DN14140_c0_g1_i2	#N/A	#N/A	-1.78	HAT5_ARATH	1.60E-44
DN14141_c1_g1_i5	#N/A	#N/A	-2.16	CAND2_ARATH	9.00E-113

Isoform ID	Log_2(#60tr/nt)	Log_2(#20tr/nt)	Log_2(#60tr/#20t	Annotation_protein	e_value_annotation
DN14145_c1_g1_i1	#N/A	#N/A	-3.42	GPT2_ARATH	0
DN14145_c1_g1_i18	#N/A	#N/A	-1.04	GPT2_ARATH	2.00E-131
DN14146_c0_g1_i10	#N/A	#N/A	0.57	MSRB2_ARATH	2.00E-81
DN14152_c0_g1_i3	#N/A	-0.86	#N/A	.	#N/A
DN14157_c0_g2_i1	#N/A	#N/A	0.88	.	#N/A
DN14182_c0_g2_i3	#N/A	#N/A	0.89	.	#N/A
DN14197_c0_g3_i4	#N/A	-1.53	#N/A	YEX1_SCHPO	1.2
DN14197_c0_g3_i7	#N/A	-1.19	#N/A	LIPA4_HUMAN	0.8
DN14201_c0_g2_i4	#N/A	#N/A	2.04	KTAP2_IXOSC	1.20E-17
DN14206_c0_g1_i3	#N/A	#N/A	-2.01	ACCO3_PETHY	1.10E-62
DN14216_c0_g1_i14	#N/A	2.19	#N/A	FAD3C_SOYBN	0
DN14222_c0_g2_i1	#N/A	#N/A	1.28	.	#N/A
DN14239_c1_g1_i5	#N/A	-0.94	#N/A	CYSD1_ARATH	4.00E-166
DN14254_c0_g2_i1	#N/A	9.1	#N/A	FULL_VITVI	4.90E-81
DN14259_c0_g3_i5	#N/A	#N/A	0.71	.	#N/A
DN14270_c0_g1_i10	#N/A	0.97	#N/A	CRK29_ARATH	2.30E-87
DN14287_c0_g1_i7	#N/A	#N/A	-10.02	PPA1_ARATH	0
DN14290_c0_g2_i5	#N/A	#N/A	1.98	TOP6B_ARATH	0
DN14296_c0_g2_i2	#N/A	#N/A	0.83	RR21_SPIOL	9.10E-46
DN14304_c1_g4_i1	#N/A	#N/A	0.91	.	#N/A
DN14305_c1_g4_i4	#N/A	#N/A	0.5	AOR_ARATH	0
DN14305_c1_g4_i7	#N/A	#N/A	0.68	AOR_ARATH	0
DN14313_c0_g1_i2	#N/A	#N/A	1.38	FER1_SOLLC	1.60E-66
DN14324_c0_g1_i1	#N/A	#N/A	0.76	.	#N/A
DN14332_c0_g1_i2	#N/A	#N/A	2.32	VDAC1_ARATH	2.30E-56
DN14355_c0_g1_i2	#N/A	#N/A	1.74	USPAL_ARATH	2.10E-13
DN14355_c0_g1_i4	#N/A	-0.75	1.5	USPAL_ARATH	2.10E-13
DN14355_c0_g4_i1	#N/A	#N/A	0.65	MDAR_ARATH	0
DN14356_c0_g3_i10	#N/A	#N/A	1.56	LEA65_ARATH	2.80E-23
DN14356_c0_g3_i7	#N/A	#N/A	0.81	LEA65_ARATH	8.50E-21
DN14367_c1_g3_i1	#N/A	#N/A	4.44	ALP1_ARATH	6.40E-23
DN14368_c1_g1_i1	#N/A	#N/A	1.26	.	#N/A
DN14368_c1_g1_i2	#N/A	#N/A	1.05	CODY_STRSV	0.8
DN14368_c1_g4_i3	#N/A	#N/A	0.86	.	#N/A
DN14377_c0_g1_i4	#N/A	1.44	#N/A	.	#N/A
DN14378_c1_g1_i7	#N/A	7.41	#N/A	Y9096_DICDI	3.90E-06
DN14382_c2_g1_i6	#N/A	#N/A	0.75	RPI2_ARATH	2.00E-131
DN14382_c2_g1_i7	#N/A	-1.68	1.81	.	#N/A
DN14382_c2_g1_i8	#N/A	1.14	#N/A	.	#N/A

Isoform ID	Log_2(#60tr/nt)	Log_2(#20tr/nt)	Log_2(#60tr/#20t	Annotation_protein	e_value_annotation
DN14382_c2_g2_i2	#N/A	#N/A	1.31	RPI3_ARATH	2.00E-131
DN14382_c2_g2_i3	#N/A	#N/A	1.08	RPI3_ARATH	1.00E-105
DN14384_c0_g1_i25	#N/A	#N/A	-0.95	AB11G_ARATH	5.00E-179
DN14386_c0_g1_i3	#N/A	#N/A	1.23	RL8_SOLLC	2.30E-85
DN14386_c0_g1_i4	#N/A	#N/A	0.96	RL83_ARATH	0
DN14386_c0_g1_i8	#N/A	#N/A	0.94	RL83_ARATH	1.00E-173
DN14386_c0_g3_i1	#N/A	#N/A	0.79	RL83_ARATH	0
DN14387_c0_g1_i4	#N/A	#N/A	0.94	PNP_BACP2	2.9
DN14392_c0_g1_i3	#N/A	#N/A	2.31	RD21B_ARATH	5.00E-116
DN14395_c0_g2_i16	#N/A	0.74	#N/A	GAE1_ARATH	0
DN14402_c0_g1_i7	#N/A	#N/A	0.77	.	#N/A
DN14405_c0_g1_i2	#N/A	#N/A	0.95	SODCP_PEA	2.10E-84
DN14405_c0_g2_i1	#N/A	#N/A	0.71	SODCP_PEA	1.00E-110
DN14405_c0_g2_i3	#N/A	#N/A	0.72	SODCP_PEA	2.00E-112
DN14410_c0_g1_i10	#N/A	#N/A	1	RL23A_FRIAG	6.70E-83
DN14410_c0_g1_i4	#N/A	#N/A	0.77	.	#N/A
DN14410_c0_g1_i8	#N/A	#N/A	1.05	RL23A_FRIAG	1.80E-82
DN14411_c2_g5_i1	#N/A	#N/A	0.75	.	#N/A
DN14411_c2_g5_i3	#N/A	#N/A	0.58	.	#N/A
DN14437_c0_g2_i3	#N/A	#N/A	5.68	TSJT1_TOBAC	3.90E-34
DN14450_c1_g1_i8	#N/A	#N/A	1.33	GATC_STRMU	1.5
DN14459_c0_g12_i1	#N/A	2.16	-1.34	.	#N/A
DN14475_c1_g2_i1	#N/A	#N/A	0.91	.	#N/A
DN14475_c2_g1_i2	#N/A	#N/A	1.1	GGPPS_HEVBR	4.00E-124
DN14486_c0_g3_i3	#N/A	#N/A	0.79	RS254_ARATH	7.50E-54
DN14486_c0_g3_i5	#N/A	#N/A	0.91	RS252_ARATH	5.50E-55
DN14486_c0_g5_i1	#N/A	#N/A	0.72	RS252_ARATH	5.50E-55
DN14486_c0_g5_i2	#N/A	#N/A	0.86	RS252_ARATH	5.50E-55
DN14487_c0_g5_i2	#N/A	#N/A	1.07	F102B_HUMAN	2.2
DN14489_c0_g1_i1	#N/A	#N/A	0.8	FLU_ARATH	3.00E-117
DN14493_c0_g2_i3	#N/A	-4.65	#N/A	YUTF_BACSU	1.40E-10
DN14494_c0_g4_i6	#N/A	-9.15	9.02	TET6_ARATH	3.00E-136
DN14497_c0_g2_i4	#N/A	#N/A	0.78	.	#N/A
DN14497_c0_g4_i1	#N/A	#N/A	0.95	R35A1_ARATH	1.80E-73
DN14500_c0_g1_i1	#N/A	#N/A	0.82	RK17_TOBAC	6.00E-103
DN14500_c0_g1_i4	#N/A	#N/A	0.68	RK17_TOBAC	1.00E-101
DN14500_c0_g5_i2	#N/A	#N/A	0.8	.	#N/A
DN14501_c0_g1_i3	#N/A	#N/A	1	GPT11_DANRE	6.70E-19
DN14503_c0_g3_i4	#N/A	#N/A	1.08	POT12_ARATH	0

Isoform ID	Log_2(#60tr/nt)	Log_2(#20tr/nt)	Log_2(#60tr/#20t	Annotation_protein	e_value_annotation
DN14503_c0_g3_i8	#N/A	#N/A	-0.58	POT12_ARATH	0
DN14512_c0_g12_i1	#N/A	-0.6	#N/A	SRC1_SOYBN	4.00E-20
DN14516_c3_g2_i2	#N/A	#N/A	-8.33	PP445_ARATH	0
DN14521_c0_g3_i12	#N/A	7.96	#N/A	TPRKB_RAT	9.70E-13
DN14526_c0_g2_i5	#N/A	#N/A	0.91	RL14_PEA	1.10E-77
DN14526_c0_g2_i6	#N/A	#N/A	1.17	RL14_PEA	3.60E-77
DN14530_c0_g1_i1	#N/A	#N/A	0.8	RLA0_SOYBN	0
DN14555_c1_g1_i3	#N/A	#N/A	0.6	CUT1D_ARATH	4.40E-41
DN14557_c0_g1_i3	#N/A	#N/A	-9.3	AAE1_ARATH	0
DN14558_c1_g1_i5	#N/A	#N/A	0.54	UCRIA_PEA	2.00E-133
DN14561_c0_g1_i1	#N/A	#N/A	1.11	WIF1_DROME	2.5
DN14567_c0_g1_i5	#N/A	3.12	#N/A	RPP2B_ARATH	8.50E-21
DN14580_c0_g3_i6	#N/A	#N/A	7.8	MURI_BACP2	0.63
DN14582_c0_g1_i1	#N/A	#N/A	0.84	RS11_SOYBN	3.00E-99
DN14582_c0_g1_i6	#N/A	#N/A	0.79	RS11_SOYBN	2.00E-99
DN14582_c0_g2_i1	#N/A	#N/A	0.87	RS11_SOYBN	5.00E-115
DN14582_c0_g2_i3	#N/A	#N/A	1	RS11_SOYBN	1.00E-115
DN14585_c0_g2_i6	#N/A	-1.09	#N/A	.	#N/A
DN14592_c0_g1_i2	#N/A	#N/A	1.15	RAC1_LOTJA	1.00E-131
DN14592_c0_g3_i2	#N/A	#N/A	-4.24	RKF1_ARATH	0
DN14598_c0_g1_i5	#N/A	#N/A	1.45	FBK9_ARATH	3.70E-84
DN14608_c0_g5_i1	#N/A	8.24	-8.37	.	#N/A
DN14634_c0_g1_i2	#N/A	#N/A	0.76	PTHM_ARATH	7.00E-114
DN14640_c0_g1_i13	#N/A	#N/A	0.63	TATA_PEA	4.60E-46
DN14642_c1_g5_i2	#N/A	#N/A	1.4	MTEF4_ARATH	0
DN14651_c0_g1_i5	#N/A	#N/A	1.13	RL37A_GOSHI	5.00E-60
DN14651_c0_g1_i6	#N/A	#N/A	1.08	RL37A_GOSHI	1.10E-59
DN14652_c0_g1_i6	#N/A	#N/A	0.79	TALB_PASMU	1.80E-58
DN14660_c0_g7_i1	#N/A	-9.01	#N/A	.	#N/A
DN14664_c1_g3_i2	#N/A	-0.98	#N/A	.	#N/A
DN14666_c0_g2_i1	#N/A	-1.02	#N/A	.	#N/A
DN14666_c0_g2_i4	#N/A	-1.04	#N/A	.	#N/A
DN14666_c0_g5_i10	#N/A	-1.33	#N/A	.	#N/A
DN14670_c0_g2_i2	#N/A	#N/A	1.53	DNJB9_MOUSE	1.20E-17
DN14671_c1_g3_i5	#N/A	#N/A	0.88	.	#N/A
DN14677_c0_g1_i5	#N/A	#N/A	-0.91	U73C5_ARATH	1.00E-169
DN14678_c0_g3_i2	#N/A	#N/A	1.04	.	#N/A
DN14678_c0_g7_i2	#N/A	#N/A	1.02	RL211_ARATH	4.00E-103
DN14680_c0_g2_i2	#N/A	#N/A	1.27	CYTI_VIGUN	8.00E-44

Isoform ID	Log2(#60tr/nt)	Log2(#20tr/nt)	Log2(#60tr/#20t	Annotation_protein	e_value_annotation
DN14680_c0_g2_i3	#N/A	#N/A	1.16	CYTI_VIGUN	8.00E-44
DN14681_c1_g4_i8	#N/A	#N/A	-7.62	.	#N/A
DN14685_c0_g4_i2	#N/A	#N/A	0.95	RL35_EUPES	3.70E-76
DN14686_c0_g2_i6	#N/A	#N/A	0.87	CB4C_ARATH	1.00E-139
DN14686_c0_g2_i7	#N/A	#N/A	0.79	CB4C_ARATH	5.00E-141
DN14686_c0_g4_i2	#N/A	#N/A	0.73	.	#N/A
DN14695_c0_g1_i16	#N/A	-2.49	#N/A	SRG1_ARATH	1.40E-78
DN14697_c0_g2_i4	#N/A	#N/A	0.8	.	#N/A
DN14698_c0_g1_i2	#N/A	1.56	#N/A	PT311_ARATH	0
DN14699_c0_g2_i10	#N/A	#N/A	0.86	.	#N/A
DN14704_c0_g1_i11	#N/A	#N/A	4.48	RR1_SPIOL	2.30E-59
DN14704_c0_g6_i2	#N/A	#N/A	0.69	RR1_SPIOL	0
DN14708_c1_g1_i10	#N/A	#N/A	0.8	GBLP_SOYBN	0
DN14711_c0_g1_i10	#N/A	8.27	#N/A	.	#N/A
DN14711_c0_g1_i3	#N/A	7.94	#N/A	.	#N/A
DN14711_c0_g1_i5	#N/A	7.36	#N/A	RB3GP_DANRE	8.60E-24
DN14716_c1_g2_i7	#N/A	#N/A	1	EPHA_MYCTU	1.30E-68
DN14731_c0_g1_i28	#N/A	-7.8	#N/A	.	#N/A
DN14739_c0_g1_i1	#N/A	#N/A	-8.07	SAC3B_ARATH	1.00E-149
DN14740_c0_g1_i14	#N/A	#N/A	0.67	CALM_MEDSA	2.00E-105
DN14740_c0_g1_i15	#N/A	#N/A	0.61	CALM_HORVU	8.00E-106
DN14740_c0_g1_i2	#N/A	#N/A	0.72	CALM_HORVU	8.00E-106
DN14749_c0_g2_i1	#N/A	#N/A	0.62	PTI13_ARATH	0
DN14751_c0_g2_i9	#N/A	1.32	#N/A	CSI1_ARATH	0
DN14756_c0_g1_i1	#N/A	7.32	-7.45	.	#N/A
DN14759_c0_g1_i1	#N/A	#N/A	0.84	ICLN_ARATH	1.80E-78
DN14779_c0_g4_i1	#N/A	#N/A	1.11	RLA2B_MAIZE	2.50E-28
DN14779_c0_g4_i4	#N/A	#N/A	1.06	RLA2B_MAIZE	4.10E-27
DN14779_c0_g6_i1	#N/A	#N/A	0.97	RLA2B_MAIZE	6.40E-31
DN14779_c0_g6_i2	#N/A	#N/A	0.92	RLA2B_MAIZE	4.80E-32
DN14783_c0_g4_i4	#N/A	#N/A	1.4	PALMD_PIG	0.31
DN14794_c0_g2_i5	#N/A	#N/A	2.52	SLD2_ARATH	3.00E-171
DN14795_c0_g1_i1	#N/A	#N/A	0.86	.	#N/A
DN14795_c0_g1_i5	#N/A	#N/A	0.66	.	#N/A
DN14795_c0_g1_i6	#N/A	#N/A	1.27	.	#N/A
DN14795_c0_g2_i4	#N/A	#N/A	1.03	.	#N/A
DN14795_c0_g4_i2	#N/A	#N/A	0.94	.	#N/A
DN14800_c0_g2_i5	#N/A	7.38	#N/A	XTH23_ARATH	9.00E-158
DN14806_c0_g1_i3	#N/A	#N/A	0.66	RL7A2_ORYSJ	3.00E-167

Isoform ID	Log_2(#60tr/nt)	Log_2(#20tr/nt)	Log_2(#60tr/#20t	Annotation_protein	e_value_annotation
DN14806_c0_g1_i8	#N/A	#N/A	0.85	RHMA_SALG2	0.16
DN14807_c0_g1_i8	#N/A	#N/A	8.66	AB3C_ARATH	2.00E-134
DN14808_c1_g6_i6	#N/A	#N/A	-9.03	HPCA1_ARATH	6.70E-92
DN14814_c0_g4_i4	#N/A	#N/A	-1.14	LHY_ARATH	5.10E-52
DN14823_c0_g2_i3	#N/A	#N/A	1	UGTK5_MANES	4.50E-71
DN14830_c2_g1_i4	#N/A	14.74	#N/A	G3PA_PEA	0
DN14833_c2_g1_i3	#N/A	7.67	#N/A	GSTX4_TOBAC	1.30E-95
DN14833_c2_g4_i10	#N/A	#N/A	0.75	GSTX4_TOBAC	1.00E-120
DN14835_c1_g4_i1	#N/A	#N/A	6.87	CDSP_ARATH	3.00E-131
DN14835_c1_g6_i1	#N/A	#N/A	0.79	RR5_ARATH	3.00E-142
DN14835_c1_g6_i4	#N/A	#N/A	0.63	RR5_ARATH	1.00E-141
DN14846_c1_g1_i18	#N/A	7.98	#N/A	SPTN2_HUMAN	9.5
DN14854_c0_g2_i1	#N/A	-0.77	#N/A	COL4_ARATH	5.00E-110
DN14854_c0_g2_i3	#N/A	-0.67	#N/A	COL4_ARATH	3.00E-111
DN14854_c0_g4_i3	#N/A	-0.85	0.93	TI172_ARATH	8.20E-90
DN14857_c0_g1_i1	#N/A	#N/A	0.62	.	#N/A
DN14857_c0_g1_i8	#N/A	#N/A	0.61	RR17_ARATH	1.40E-52
DN14858_c0_g1_i1	#N/A	#N/A	0.88	RS8_MAIZE	5.00E-142
DN14858_c0_g1_i3	#N/A	#N/A	0.75	RS8_MAIZE	2.00E-140
DN14861_c0_g5_i1	#N/A	#N/A	0.63	MAF1_SOLLC	8.50E-45
DN14861_c0_g5_i2	#N/A	#N/A	0.92	MAF1_SOLLC	2.00E-46
DN14866_c0_g3_i5	#N/A	#N/A	2.14	AF111_ALTAL	9.1
DN14871_c2_g1_i6	#N/A	2.66	#N/A	KN4B_ARATH	0.42
DN14871_c2_g2_i13	#N/A	#N/A	5.33	HIP8_ARATH	9.40E-45
DN14874_c1_g2_i7	#N/A	#N/A	0.75	YUGF_BACSU	5.50E-15
DN14876_c0_g2_i6	#N/A	-1.55	#N/A	.	#N/A
DN14879_c0_g1_i12	#N/A	#N/A	-7.66	.	#N/A
DN14886_c0_g3_i10	#N/A	2.08	#N/A	RPS4B_ARATH	0.002
DN14886_c0_g3_i7	#N/A	5.95	#N/A	RPS4B_ARATH	1.70E-07
DN14886_c0_g3_i8	#N/A	1.81	-1.67	RPS4B_ARATH	0.037
DN14892_c0_g1_i4	#N/A	#N/A	0.57	CB11_SOLLC	3.00E-148
DN14898_c0_g2_i5	#N/A	#N/A	-9.36	ACPM3_ARATH	4.30E-36
DN14898_c0_g2_i6	#N/A	#N/A	9.1	ACPM3_ARATH	4.30E-36
DN14903_c0_g1_i10	#N/A	#N/A	0.71	.	#N/A
DN14904_c1_g1_i12	#N/A	#N/A	-8.13	RAB7_PEA	9.00E-133
DN14904_c1_g3_i9	#N/A	-2.08	#N/A	C3H12_ARATH	4.70E-73
DN14905_c0_g1_i2	#N/A	#N/A	8.71	IPT9_ARATH	5.00E-82
DN14915_c0_g1_i1	#N/A	#N/A	2.44	TM45B_BOVIN	0.003
DN14936_c0_g1_i1	#N/A	#N/A	-2.18	.	#N/A

Isoform ID	Log_2(#60tr/nt)	Log_2(#20tr/nt)	Log_2(#60tr/#20t	Annotation_protein	e_value_annotation
DN14937_c0_g1_i5	#N/A	#N/A	-8.55	.	#N/A
DN14949_c0_g1_i13	#N/A	-1.6	#N/A	QORH_ARATH	4.00E-172
DN14955_c0_g3_i5	#N/A	#N/A	6.61	G2OX1_PEA	0
DN14957_c0_g1_i5	#N/A	1.63	-1.09	JOIN_SOLLC	1.20E-53
DN14982_c0_g2_i1	#N/A	#N/A	1	MBD10_ARATH	2.10E-49
DN14997_c2_g1_i16	#N/A	10.35	-10.48	PRR73_ORYSJ	1.00E-51
DN15000_c0_g1_i4	#N/A	#N/A	0.67	.	#N/A
DN15003_c0_g3_i1	#N/A	#N/A	0.77	.	#N/A
DN15011_c0_g3_i6	#N/A	#N/A	0.97	.	#N/A
DN15017_c3_g4_i2	#N/A	-1.36	#N/A	.	#N/A
DN15019_c0_g3_i10	#N/A	-3.07	#N/A	CYSK_CITLA	3.20E-94
DN15023_c0_g1_i3	#N/A	#N/A	0.9	.	#N/A
DN15023_c0_g1_i4	#N/A	#N/A	0.81	.	#N/A
DN15023_c0_g3_i1	#N/A	#N/A	0.83	.	#N/A
DN15023_c0_g3_i2	#N/A	#N/A	1.21	.	#N/A
DN15027_c0_g1_i4	#N/A	#N/A	1.33	ATS3B_ARATH	1.50E-58
DN15027_c0_g1_i8	#N/A	0.9	#N/A	TMK1_ARATH	0
DN15030_c0_g1_i1	#N/A	-0.86	#N/A	TRXM_SPIOL	1.10E-67
DN15033_c0_g2_i5	#N/A	7.72	#N/A	XTH2_SOYBN	2.60E-97
DN15036_c0_g2_i1	#N/A	#N/A	0.99	.	#N/A
DN15036_c0_g2_i10	#N/A	#N/A	0.69	.	#N/A
DN15036_c0_g2_i17	#N/A	#N/A	1.08	RL261_ARATH	1.10E-86
DN15036_c0_g2_i21	#N/A	#N/A	0.96	.	#N/A
DN15037_c0_g2_i5	#N/A	-0.83	#N/A	CBSX3_ARATH	2.00E-116
DN15042_c0_g2_i7	#N/A	-0.79	0.93	RGGB_ARATH	8.80E-47
DN15049_c0_g2_i4	#N/A	-0.73	0.97	SYE_CYTH3	0.055
DN15058_c0_g3_i6	#N/A	#N/A	0.61	.	#N/A
DN15063_c0_g1_i6	#N/A	#N/A	1.54	SIRB_ARATH	7.00E-79
DN15073_c0_g3_i1	#N/A	#N/A	0.68	MDHM_FRAAN	0
DN15077_c0_g1_i5	#N/A	#N/A	-1.31	PPA1_ARATH	0
DN15080_c0_g1_i4	#N/A	#N/A	0.57	.	#N/A
DN15080_c0_g1_i7	#N/A	#N/A	0.65	.	#N/A
DN15097_c0_g1_i14	#N/A	#N/A	0.73	RR9_SPIOL	3.00E-109
DN15103_c0_g1_i3	#N/A	#N/A	1.21	YKM6_SCHPO	9.30E-33
DN15104_c0_g1_i5	#N/A	#N/A	-7.41	.	#N/A
DN15104_c0_g1_i9	#N/A	-8.46	#N/A	CYL2_ARATH	2.70E-77
DN15104_c0_g7_i2	#N/A	#N/A	2.14	C7263_MEDTR	1.00E-113
DN15109_c0_g2_i6	#N/A	#N/A	1.24	SODC_SOYBN	7.30E-58
DN15110_c0_g2_i5	#N/A	#N/A	1.25	.	#N/A

Isoform ID	Log_2(#60tr/nt)	Log_2(#20tr/nt)	Log_2(#60tr/#20t	Annotation_protein	e_value_annotation
DN15112_c1_g3_i11	#N/A	#N/A	-0.57	PRR95_ORYSJ	1.70E-22
DN15113_c0_g1_i2	#N/A	2.03	#N/A	TMVRN_NICGU	3.60E-14
DN15127_c0_g1_i5	#N/A	-8.88	#N/A	TPPC1_DICDI	2.50E-33
DN15131_c0_g1_i17	#N/A	#N/A	0.95	ATL6_ARATH	5.10E-44
DN15132_c0_g3_i2	#N/A	-1.37	#N/A	.	#N/A
DN15138_c0_g1_i2	#N/A	-8.92	#N/A	.	#N/A
DN15144_c0_g1_i3	#N/A	#N/A	0.64	ALF_CICAR	0
DN15160_c1_g1_i6	#N/A	-1.52	#N/A	WSD1_ARATH	0.007
DN15165_c0_g3_i3	#N/A	#N/A	0.8	PHB3_ARATH	5.00E-170
DN15165_c0_g3_i4	#N/A	#N/A	0.81	PHB3_ARATH	1.00E-171
DN15174_c0_g1_i14	#N/A	-1.17	#N/A	CB23_PEA	1.00E-175
DN15174_c0_g1_i16	#N/A	#N/A	0.85	CB13_SOLLC	1.00E-110
DN15180_c0_g2_i2	#N/A	#N/A	1.19	RL18A_CASSA	1.00E-121
DN15190_c0_g7_i1	#N/A	-0.83	#N/A	.	#N/A
DN15191_c0_g2_i13	#N/A	#N/A	2.28	AHSA1_HUMAN	0.00022
DN15193_c0_g1_i3	#N/A	-0.85	#N/A	.	#N/A
DN15205_c0_g1_i3	#N/A	-0.99	#N/A	YS88_CAEEL	1.3
DN15205_c0_g1_i7	#N/A	-0.99	#N/A	GOE_DROME	1.1
DN15212_c0_g2_i2	#N/A	#N/A	-8.23	.	#N/A
DN15220_c0_g1_i11	#N/A	#N/A	0.54	BTF3_ARATH	9.20E-89
DN15220_c0_g1_i7	#N/A	#N/A	0.6	CPLX_HIRME	0.11
DN15220_c0_g1_i8	#N/A	#N/A	0.59	BTF3_ARATH	4.40E-88
DN15237_c0_g1_i14	#N/A	#N/A	0.68	FK163_ARATH	1.00E-105
DN15238_c0_g3_i1	#N/A	#N/A	0.68	SUI11_ARATH	2.40E-68
DN15238_c0_g3_i5	#N/A	#N/A	0.81	SUI11_ARATH	5.60E-73
DN15248_c1_g4_i10	#N/A	#N/A	1.12	RIBL_METP4	0.19
DN15262_c0_g1_i6	#N/A	#N/A	0.66	PSAD_CUCSA	2.00E-116
DN15270_c0_g3_i7	#N/A	#N/A	0.96	RK29_ARATH	7.60E-53
DN15277_c0_g1_i2	#N/A	#N/A	-2.06	GLOX_VITPS	0
DN15279_c0_g1_i12	#N/A	#N/A	-1.63	RD22_ARATH	1.20E-69
DN15284_c0_g1_i2	#N/A	#N/A	-2.33	TOR_ARATH	0
DN15289_c0_g1_i3	#N/A	#N/A	1.07	RLA11_ARATH	1.00E-22
DN15289_c0_g1_i4	#N/A	#N/A	1.36	RLA11_ARATH	2.70E-22
DN15289_c0_g1_i6	#N/A	#N/A	1.25	RLA11_ARATH	7.50E-23
DN15303_c0_g1_i3	#N/A	#N/A	0.68	ANM10_ARATH	0
DN15307_c0_g1_i6	#N/A	#N/A	0.65	EIF3K_ORYSJ	1.00E-131
DN15313_c0_g2_i5	#N/A	-1.46	#N/A	NAC22_ARATH	1.00E-125
DN15326_c0_g1_i19	#N/A	0.83	#N/A	FLA2_ARATH	6.00E-152
DN15329_c0_g2_i7	#N/A	#N/A	-8.28	TSAC_PSYIN	0.016

Isoform ID	Log_2(#60tr/nt)	Log_2(#20tr/nt)	Log_2(#60tr/#20t	Annotation_protein	e_value_annotation
DN15330_c2_g1_i5	#N/A	#N/A	5.57	Y5986_ARATH	5.10E-50
DN15342_c0_g1_i2	#N/A	#N/A	0.57	CLPP5_ARATH	6.00E-122
DN15343_c0_g4_i2	#N/A	-1.3	#N/A	.	#N/A
DN15347_c0_g1_i2	#N/A	#N/A	9.13	STN8_ARATH	2.00E-176
DN15353_c0_g1_i3	#N/A	2.79	#N/A	TIP21_ARATH	9.00E-133
DN15354_c0_g1_i2	#N/A	#N/A	0.71	VATD_ARATH	8.00E-145
DN15361_c0_g2_i1	#N/A	-10.6	11.38	RGGB_ARATH	2.30E-87
DN15361_c0_g2_i3	#N/A	#N/A	0.7	RGGB_ARATH	2.70E-88
DN15365_c0_g3_i1	#N/A	#N/A	-7.9	U83A1_ARATH	2.00E-77
DN15369_c0_g4_i5	#N/A	#N/A	1.47	LIRP1_ARATH	2.80E-29
DN15371_c1_g2_i2	#N/A	-6.12	#N/A	BH068_ARATH	1.50E-67
DN15375_c0_g4_i6	#N/A	#N/A	1	RL4A_ARATH	2.20E-73
DN15375_c0_g4_i8	#N/A	#N/A	0.78	RL4A_ARATH	0
DN15378_c0_g3_i9	#N/A	2.05	#N/A	.	#N/A
DN15381_c0_g1_i3	#N/A	#N/A	0.64	P5CR_SOYBN	0
DN15381_c0_g1_i4	#N/A	#N/A	-1.18	NOV_ARATH	0
DN15381_c0_g1_i8	#N/A	9.75	#N/A	PSBB_SOYBN	0
DN15402_c0_g1_i8	#N/A	#N/A	0.63	CB23_SOYBN	0
DN15402_c0_g1_i9	#N/A	#N/A	0.62	CB23_SOYBN	0
DN15406_c0_g1_i21	#N/A	-0.94	#N/A	.	#N/A
DN15408_c0_g1_i1	#N/A	#N/A	0.87	RS18_ARATH	2.00E-104
DN15408_c0_g1_i2	#N/A	#N/A	0.96	RS18_ARATH	5.00E-105
DN15408_c0_g1_i3	#N/A	#N/A	1	RS18_ARATH	8.00E-106
DN15408_c0_g1_i4	#N/A	#N/A	1.1	RS18_ARATH	1.00E-104
DN15408_c0_g1_i5	#N/A	#N/A	1.03	RS18_ARATH	1.00E-104
DN15408_c0_g5_i1	#N/A	#N/A	0.96	RS18_ARATH	5.00E-105
DN15417_c0_g1_i1	#N/A	#N/A	-0.89	ATG2_VANPO	1.4
DN15420_c0_g1_i4	#N/A	#N/A	0.98	RS191_ARATH	1.50E-92
DN15420_c0_g1_i6	#N/A	#N/A	0.86	RS191_ARATH	1.00E-92
DN15422_c1_g1_i3	#N/A	#N/A	9.49	SCAM_PEA	2.00E-167
DN15423_c0_g4_i5	#N/A	1.76	-1.24	EF105_ARATH	2.00E-51
DN15431_c0_g2_i6	#N/A	#N/A	-5.79	UAF30_SCHPO	7.20E-20
DN15432_c0_g1_i20	#N/A	1.42	#N/A	SNL6_ORYSJ	6.00E-101
DN15433_c0_g2_i4	#N/A	#N/A	9.22	RBPS2_XENLA	2.70E-16
DN15439_c0_g2_i2	#N/A	#N/A	0.83	RL14_PEA	8.10E-78
DN15439_c0_g5_i1	#N/A	-0.93	#N/A	.	#N/A
DN15439_c0_g5_i4	#N/A	-1	#N/A	.	#N/A
DN15439_c0_g5_i5	#N/A	-1.06	#N/A	.	#N/A
DN15440_c0_g6_i3	#N/A	-0.78	0.84	EF106_ARATH	2.60E-41

Isoform ID	Log_2(#60tr/nt)	Log_2(#20tr/nt)	Log_2(#60tr/#20t	Annotation_protein	e_value_annotation
DN15443_c0_g2_i1	#N/A	#N/A	1.31	CINA_BACC2	0.4
DN15448_c0_g5_i8	#N/A	#N/A	0.68	TL09_SPIOL	0.59
DN15449_c1_g5_i1	#N/A	-9.23	#N/A	.	#N/A
DN15456_c1_g1_i1	#N/A	#N/A	1.05	RL34_TOBAC	3.70E-78
DN15456_c1_g1_i3	#N/A	#N/A	0.96	RL34_TOBAC	1.40E-77
DN15457_c0_g3_i1	#N/A	#N/A	0.97	TL1Y_ARATH	4.80E-86
DN15458_c0_g1_i10	#N/A	#N/A	-0.75	PATL3_ARATH	8.10E-06
DN15459_c0_g1_i3	#N/A	#N/A	0.95	RL363_ARATH	4.80E-57
DN15459_c0_g2_i1	#N/A	#N/A	1.07	RL363_ARATH	1.10E-57
DN15459_c0_g2_i5	#N/A	#N/A	0.97	RL363_ARATH	1.00E-57
DN15459_c0_g2_i6	#N/A	#N/A	0.98	RL363_ARATH	4.10E-57
DN15477_c0_g3_i7	#N/A	#N/A	0.9	YCF33_CYAPA	0.003
DN15489_c0_g3_i8	#N/A	#N/A	8.65	.	#N/A
DN15490_c0_g1_i4	#N/A	#N/A	-1.51	AOX4_ARATH	1.00E-170
DN15497_c1_g2_i5	#N/A	#N/A	1.54	CHLG_ARATH	0
DN15499_c0_g1_i6	#N/A	#N/A	1.01	PNSB3_ARATH	5.10E-73
DN15506_c0_g2_i2	#N/A	#N/A	6.34	AHP1_ARATH	5.70E-39
DN15508_c0_g9_i4	#N/A	7.54	#N/A	.	#N/A
DN15516_c0_g1_i9	#N/A	#N/A	0.81	CP31A_ARATH	4.00E-114
DN15521_c0_g1_i2	#N/A	#N/A	0.75	.	#N/A
DN15521_c0_g1_i3	#N/A	#N/A	1.13	.	#N/A
DN15521_c0_g1_i4	#N/A	#N/A	0.84	.	#N/A
DN15521_c0_g2_i1	#N/A	#N/A	1.56	RS174_ARATH	1.30E-79
DN15525_c0_g2_i5	#N/A	-8.44	10.72	NPS13_ARATH	3.00E-160
DN15528_c0_g1_i11	#N/A	-0.84	#N/A	VPE1_PHAVU	4.60E-67
DN15537_c0_g2_i1	#N/A	#N/A	2.05	.	#N/A
DN15540_c0_g1_i1	#N/A	#N/A	0.71	HSP7C_PETHY	9.00E-142
DN15540_c0_g2_i11	#N/A	1.64	-1.5	HS704_ARATH	0
DN15546_c0_g1_i6	#N/A	#N/A	0.62	RK19_SPIOL	7.10E-76
DN15559_c0_g1_i14	#N/A	#N/A	1.2	RS23_EUPES	5.00E-100
DN15559_c0_g1_i15	#N/A	#N/A	1.08	RS23_EUPES	1.50E-96
DN15559_c0_g1_i8	#N/A	#N/A	0.7	RS23_EUPES	4.50E-75
DN15559_c0_g3_i2	#N/A	#N/A	2.14	RS23_EUPES	5.00E-100
DN15559_c0_g3_i4	#N/A	#N/A	1.23	RS23_EUPES	5.00E-101
DN15562_c0_g3_i1	#N/A	#N/A	0.95	.	#N/A
DN15566_c0_g1_i8	#N/A	8.34	#N/A	RLP43_ARATH	3.00E-34
DN15570_c0_g2_i1	#N/A	7.26	-7.39	NBP35_ARATH	0
DN15570_c0_g2_i2	#N/A	#N/A	-7.48	NBP35_ARATH	0
DN15572_c1_g3_i2	#N/A	#N/A	0.6	.	#N/A

Isoform ID	Log_2(#60tr/nt)	Log_2(#20tr/nt)	Log_2(#60tr/#20t	Annotation_protein	e_value_annotation
DN15575_c0_g1_i5	#N/A	#N/A	-8.11	MUC70_ARATH	6.00E-103
DN15579_c0_g1_i1	#N/A	5.56	#N/A	CY11_SOLTU	0
DN15579_c0_g1_i12	#N/A	8.95	#N/A	CY11_SOLTU	0
DN15579_c0_g1_i3	#N/A	9.56	#N/A	CY11_SOLTU	0
DN15582_c0_g1_i3	#N/A	#N/A	-10.33	GALK1_ARATH	0
DN15588_c0_g1_i1	#N/A	9.26	#N/A	.	#N/A
DN15589_c0_g2_i8	#N/A	#N/A	0.77	STEP1_ARATH	1.90E-44
DN15593_c0_g2_i10	#N/A	#N/A	0.79	UGT9_CATRO	1.2
DN15593_c0_g2_i6	#N/A	#N/A	0.75	RK5_ARATH	6.00E-135
DN15593_c0_g2_i9	#N/A	#N/A	0.99	UGT9_CATRO	2
DN15596_c1_g1_i1	#N/A	1.05	#N/A	CESA9_ARATH	0
DN15596_c1_g1_i7	#N/A	0.91	#N/A	CESA9_ARATH	2.00E-176
DN15599_c1_g1_i1	#N/A	1.5	#N/A	EXTN3_ARATH	0.013
DN15600_c0_g2_i2	#N/A	#N/A	1.57	PLA20_ARATH	1.00E-142
DN15604_c0_g1_i3	#N/A	8.64	#N/A	FLXL3_ARATH	1.20E-81
DN15609_c0_g1_i10	#N/A	#N/A	-0.96	IAA17_ARATH	4.10E-58
DN15611_c0_g1_i1	#N/A	#N/A	0.85	RL30_LUPLU	2.30E-77
DN15611_c0_g1_i2	#N/A	#N/A	0.8	RL30_LUPLU	2.00E-77
DN15611_c0_g1_i4	#N/A	#N/A	1.47	RL30_LUPLU	2.00E-77
DN15611_c0_g1_i5	#N/A	#N/A	0.8	RL30_LUPLU	1.50E-77
DN15613_c1_g1_i2	#N/A	#N/A	1.12	TI201_ARATH	2.00E-123
DN15613_c1_g1_i8	#N/A	#N/A	0.58	TIC20_PEA	1.00E-123
DN15618_c0_g2_i2	#N/A	#N/A	0.76	ACP1_CASGL	5.60E-44
DN15620_c0_g3_i3	#N/A	#N/A	0.94	RL23_ARATH	4.50E-97
DN15620_c0_g3_i7	#N/A	#N/A	1.12	RL23_ARATH	4.50E-97
DN15629_c0_g1_i26	#N/A	#N/A	1.31	BH068_ARATH	7.10E-89
DN15647_c1_g4_i8	#N/A	#N/A	-8.03	.	#N/A
DN15658_c0_g2_i13	#N/A	#N/A	-1.1	APRR7_ARATH	9.00E-118
DN15659_c0_g1_i20	#N/A	1.64	-3.51	.	#N/A
DN15659_c0_g1_i5	#N/A	#N/A	-0.88	PRR95_ORYSJ	1.20E-67
DN15661_c0_g2_i8	#N/A	#N/A	5.86	CXE13_ARATH	4.40E-61
DN15664_c0_g2_i3	#N/A	#N/A	0.86	MEE14_ARATH	3.20E-63
DN15671_c0_g2_i2	#N/A	#N/A	0.86	NDK4_ARATH	5.00E-136
DN15681_c0_g2_i1	#N/A	4.79	-2.28	XTH22_ARATH	3.00E-159
DN15683_c0_g4_i6	#N/A	#N/A	0.6	FBK30_ARATH	7.00E-122
DN15693_c1_g1_i7	#N/A	#N/A	2.96	LCMT1_ARATH	3.10E-47
DN15694_c0_g3_i1	#N/A	9.51	#N/A	TRL4_ARATH	7.50E-74
DN15694_c0_g3_i10	#N/A	-8.98	#N/A	TRL4_ARATH	7.50E-74
DN15696_c0_g2_i11	#N/A	#N/A	0.81	BAS1B_ARATH	2.00E-140

Isoform ID	Log_2(#60tr/nt)	Log_2(#20tr/nt)	Log_2(#60tr/#20t	Annotation_protein	e_value_annotation
DN15696_c0_g2_i5	#N/A	#N/A	0.85	BAS1B_ARATH	6.00E-141
DN15701_c0_g4_i4	#N/A	#N/A	0.69	LHCA6_ARATH	3.00E-140
DN15706_c0_g1_i18	#N/A	#N/A	-8.03	PYRE_CHLPM	2.4
DN15712_c0_g1_i1	#N/A	#N/A	7.61	.	#N/A
DN15720_c0_g1_i6	#N/A	#N/A	8.34	TLP3_ARATH	9.00E-177
DN15725_c0_g4_i5	#N/A	#N/A	-0.71	Y4729_ARATH	6.00E-164
DN15727_c0_g2_i18	#N/A	2.34	-1.1	FRI1_SOYBN	0
DN15727_c0_g2_i2	#N/A	#N/A	-1	FRI_MALBX	6.00E-180
DN15729_c0_g2_i16	#N/A	4.8	#N/A	M810_ARATH	2.60E-46
DN15729_c0_g2_i3	#N/A	9.06	#N/A	M810_ARATH	2.60E-46
DN15738_c0_g1_i1	#N/A	#N/A	0.7	KGLT_PETHY	1.00E-136
DN15747_c0_g1_i4	#N/A	#N/A	0.89	GLO2_FRATN	1.2
DN15753_c0_g2_i1	#N/A	#N/A	1.02	LOXX_SOYBN	0
DN15756_c0_g1_i1	#N/A	#N/A	0.73	MY1R1_SOLTU	5.30E-66
DN15756_c0_g3_i1	#N/A	-0.86	#N/A	.	#N/A
DN15766_c1_g3_i6	#N/A	#N/A	1.62	CAN2_ARATH	1.00E-132
DN15770_c1_g1_i1	#N/A	#N/A	8.88	GAT1_ARATH	6.00E-176
DN15777_c1_g1_i27	#N/A	#N/A	-7.63	.	#N/A
DN15783_c0_g2_i11	#N/A	#N/A	1.47	Y4844_ARATH	4.70E-61
DN15783_c0_g2_i12	#N/A	#N/A	0.71	Y4844_ARATH	1.50E-61
DN15783_c0_g2_i16	#N/A	#N/A	1.54	Y4844_ARATH	1.60E-61
DN15783_c0_g2_i9	#N/A	#N/A	1.16	Y4844_ARATH	1.50E-61
DN15788_c0_g1_i5	#N/A	#N/A	9.13	MYB60_VITVI	5.00E-127
DN15789_c0_g2_i7	#N/A	#N/A	1.92	.	#N/A
DN15790_c2_g3_i1	#N/A	1.86	#N/A	PBP1_ARATH	3.40E-47
DN15791_c0_g1_i2	#N/A	-2.95	#N/A	STR8_ARATH	2.00E-57
DN15792_c0_g1_i3	#N/A	-1.54	#N/A	CSN5B_ARATH	0
DN15793_c0_g1_i11	#N/A	#N/A	0.85	RL132_BRANA	1.00E-127
DN15794_c0_g2_i1	#N/A	#N/A	-0.76	MURG_BUCAT	5.3
DN15797_c0_g1_i1	#N/A	#N/A	-7.71	.	#N/A
DN15805_c0_g2_i1	#N/A	#N/A	1.06	RL12_PRUAR	3.00E-107
DN15805_c0_g2_i2	#N/A	#N/A	0.92	RL12_PRUAR	3.00E-107
DN15809_c0_g3_i6	#N/A	#N/A	0.59	RK24_TOBAC	2.30E-97
DN15816_c0_g1_i10	#N/A	#N/A	1.1	RL23A_FRIAG	1.10E-81
DN15816_c0_g1_i14	#N/A	#N/A	1	MT21A_HUMAN	1.2
DN15825_c0_g1_i4	#N/A	#N/A	1.58	AT2L1_DANRE	3.3
DN15830_c0_g1_i6	#N/A	#N/A	0.85	GATC_VITVI	1.20E-47
DN15835_c0_g1_i5	#N/A	#N/A	0.74	RIDA_ARATH	1.60E-79
DN15840_c2_g2_i1	#N/A	#N/A	0.65	NDK2_PEA	4.00E-117

Isoform ID	Log_2(#60tr/nt)	Log_2(#20tr/nt)	Log_2(#60tr/#20t	Annotation_protein	e_value_annotation
DN15840_c2_g2_i5	#N/A	#N/A	0.72	NDK2_PEA	2.00E-119
DN15844_c0_g1_i18	#N/A	#N/A	0.83	RP25L_MOUSE	2.60E-20
DN15844_c0_g1_i4	#N/A	#N/A	0.98	RP25L_MOUSE	4.10E-21
DN15844_c0_g1_i6	#N/A	#N/A	0.76	RP25L_MOUSE	2.60E-20
DN15848_c0_g2_i10	#N/A	#N/A	0.79	RL15_PETHY	4.00E-133
DN15848_c0_g2_i11	#N/A	#N/A	-7.53	RL15_PETHY	1.40E-93
DN15848_c0_g2_i13	#N/A	#N/A	0.97	RL15_PETHY	2.00E-121
DN15848_c0_g2_i5	#N/A	#N/A	1.44	RL15_PETHY	1.40E-93
DN15848_c0_g2_i7	#N/A	#N/A	0.91	RL15_PETHY	4.00E-133
DN15848_c0_g2_i9	#N/A	#N/A	1.09	RL15_PETHY	1.00E-132
DN15851_c0_g2_i8	#N/A	-0.58	#N/A	BAM3_ARATH	1.00E-174
DN15855_c0_g1_i2	#N/A	#N/A	8.71	NCBP2_ARATH	3.30E-42
DN15867_c0_g1_i4	#N/A	1.46	#N/A	E70B1_ARATH	0
DN15868_c0_g1_i6	#N/A	#N/A	-8.14	.	#N/A
DN15880_c0_g1_i1	#N/A	-0.79	#N/A	ECH2M_ARATH	3.00E-135
DN15884_c0_g2_i12	#N/A	#N/A	1.83	.	#N/A
DN15899_c0_g2_i2	#N/A	-8.43	8.91	SCP42_ARATH	8.00E-100
DN15905_c0_g1_i2	#N/A	#N/A	0.76	MSI2_ARATH	0
DN15909_c0_g3_i1	#N/A	#N/A	1.45	.	#N/A
DN15912_c0_g2_i9	#N/A	-0.63	#N/A	ALFC_SPIOL	5.00E-155
DN15923_c0_g1_i18	#N/A	#N/A	0.54	DNAJ_THENN	3.50E-08
DN15932_c0_g4_i1	#N/A	#N/A	0.63	EIF3F_ARATH	3.00E-158
DN15935_c0_g4_i3	#N/A	#N/A	0.88	RS261_ARATH	4.40E-68
DN15939_c0_g1_i7	#N/A	#N/A	0.99	FK201_ARATH	2.00E-90
DN15939_c0_g3_i2	#N/A	8.5	#N/A	SYP61_ARATH	0.00065
DN15945_c0_g3_i4	#N/A	#N/A	0.6	.	#N/A
DN15946_c0_g1_i5	#N/A	#N/A	-7.76	Y1498_MYCTU	0.008
DN15948_c1_g2_i2	#N/A	#N/A	0.9	.	#N/A
DN15948_c1_g2_i3	#N/A	-1.2	1.12	.	#N/A
DN15951_c0_g3_i5	#N/A	#N/A	9.94	TET19_ARATH	2.00E-97
DN15954_c0_g5_i3	#N/A	-7.76	8.27	SMR6_ARATH	2.80E-26
DN15966_c0_g1_i3	#N/A	-1.56	#N/A	.	#N/A
DN15966_c0_g1_i5	#N/A	-1.19	#N/A	.	#N/A
DN15967_c1_g1_i6	#N/A	#N/A	0.94	.	#N/A
DN15967_c1_g1_i7	#N/A	#N/A	0.84	RR13_ARATH	1.10E-80
DN15973_c0_g3_i4	#N/A	#N/A	0.71	EPHA_MYCTU	1.30E-59
DN15979_c1_g4_i7	#N/A	#N/A	-7.47	PI5K8_ARATH	1.90E-28
DN15986_c1_g1_i12	#N/A	#N/A	0.58	EF1D_PIMBR	5.00E-121
DN15992_c2_g1_i1	#N/A	#N/A	0.76	PP314_ARATH	1.00E-107

Isoform ID	Log_2(#60tr/nt)	Log_2(#20tr/nt)	Log_2(#60tr/#20t	Annotation_protein	e_value_annotation
DN15992_c2_g4_i2	#N/A	#N/A	1.29	PLA16_ARATH	0
DN15997_c0_g1_i12	#N/A	#N/A	1.28	RS242_ARATH	3.00E-81
DN15997_c0_g1_i13	#N/A	#N/A	0.83	RS242_ARATH	4.20E-84
DN15999_c1_g2_i7	#N/A	#N/A	-0.73	AB1K7_ARATH	0
DN16002_c0_g1_i8	#N/A	#N/A	0.99	OP163_ARATH	2.80E-76
DN16003_c0_g1_i4	#N/A	-6.07	#N/A	SINA3_ARATH	0
DN16011_c0_g3_i2	#N/A	#N/A	-7.34	FRS6_ARATH	3.00E-164
DN16021_c0_g4_i10	#N/A	1.65	#N/A	REP1_USTMA	6.40E-05
DN16021_c0_g4_i6	#N/A	1.7	#N/A	Z512B_HUMAN	7.30E-07
DN16022_c0_g4_i2	#N/A	#N/A	0.89	BAMS_GLYGL	0
DN16022_c0_g5_i7	#N/A	-0.76	#N/A	H2A_PETCR	6.60E-78
DN16030_c0_g1_i6	#N/A	-9.33	#N/A	ZFPL1_CAEEL	5.70E-29
DN16035_c0_g1_i5	#N/A	#N/A	0.7	GSA_SOYBN	0
DN16039_c0_g1_i10	#N/A	#N/A	-8.48	AGT21_ARATH	0
DN16039_c0_g1_i5	#N/A	-8.09	#N/A	AGT21_ARATH	0
DN16041_c0_g1_i1	#N/A	#N/A	1.13	.	#N/A
DN16042_c0_g1_i23	#N/A	#N/A	-1.23	Y0G0_VIBCH	1.7
DN16050_c1_g3_i7	#N/A	#N/A	9.21	EF118_ARATH	4.10E-39
DN16054_c1_g3_i2	#N/A	#N/A	1.38	PYRC_CHESB	3.7
DN16063_c0_g3_i1	#N/A	2.36	-3.24	CDR1_HUMAN	0.028
DN16064_c1_g1_i5	#N/A	#N/A	3.83	DAPAT_ORYSJ	3.00E-101
DN16064_c1_g1_i6	#N/A	#N/A	0.66	DAPAT_ARATH	0
DN16065_c0_g1_i10	#N/A	2.16	-1.55	LRK10_WHEAT	1.30E-74
DN16066_c0_g3_i4	#N/A	#N/A	0.66	.	#N/A
DN16068_c0_g1_i3	#N/A	#N/A	0.64	RL72_ARATH	4.00E-152
DN16084_c0_g2_i1	#N/A	#N/A	0.92	EF1B1_ARATH	6.00E-108
DN16084_c0_g2_i4	#N/A	#N/A	0.69	EF1B1_ARATH	3.00E-107
DN16084_c0_g3_i2	#N/A	#N/A	0.87	EF1B1_ARATH	9.00E-104
DN16088_c0_g4_i5	#N/A	#N/A	1.3	VSPA_SOYBN	8.00E-107
DN16095_c0_g1_i7	#N/A	#N/A	-0.86	OPLA_ARATH	0
DN16101_c0_g1_i12	#N/A	-0.96	#N/A	LOR12_ARATH	4.60E-88
DN16104_c0_g2_i9	#N/A	#N/A	-7.86	BBX22_ARATH	5.00E-78
DN16106_c0_g1_i1	#N/A	#N/A	-1.07	PMI2_ARATH	1.40E-58
DN16106_c0_g1_i2	#N/A	#N/A	-1.05	PMI2_ARATH	9.60E-55
DN16113_c3_g5_i3	#N/A	#N/A	10.05	GDL83_ARATH	2.00E-113
DN16113_c3_g5_i9	#N/A	1.31	#N/A	GDL83_ARATH	2.00E-136
DN16116_c1_g1_i6	#N/A	8.35	#N/A	BH070_ARATH	8.60E-80
DN16117_c0_g2_i1	#N/A	-0.83	#N/A	H2AV3_ORYSJ	9.80E-57
DN16126_c0_g2_i2	#N/A	#N/A	0.82	RS6_ASPOF	6.00E-112

Isoform ID	Log_2(#60tr/nt)	Log_2(#20tr/nt)	Log_2(#60tr/#20t	Annotation_protein	e_value_annotation
DN16126_c0_g2_i4	#N/A	#N/A	1.64	RS6_ASPOF	6.90E-80
DN16126_c0_g2_i6	#N/A	#N/A	0.93	RS6_ASPOF	1.00E-112
DN16126_c0_g2_i8	#N/A	#N/A	0.86	RS6_ASPOF	2.00E-169
DN16126_c0_g2_i9	#N/A	#N/A	0.79	RS6_ASPOF	5.00E-169
DN16128_c0_g2_i9	#N/A	#N/A	0.87	FB76_ARATH	4.00E-168
DN16136_c0_g2_i13	#N/A	-7.78	#N/A	.	#N/A
DN16137_c0_g2_i9	#N/A	#N/A	5.51	ZDHC1_ARATH	6.40E-30
DN16148_c1_g1_i10	#N/A	#N/A	2.18	IFRD1_PIG	4.10E-16
DN16148_c1_g1_i2	#N/A	#N/A	-0.68	IFRD1_PIG	6.50E-07
DN16150_c1_g5_i5	#N/A	#N/A	1.87	RBCX2_ARATH	6.80E-78
DN16156_c0_g1_i10	#N/A	4.43	#N/A	ACCH1_ARATH	9.30E-83
DN16164_c0_g1_i2	#N/A	#N/A	0.67	RAF2_ARATH	2.00E-162
DN16165_c0_g5_i5	#N/A	#N/A	0.95	ACP1_CASGL	6.80E-44
DN16171_c0_g4_i4	#N/A	-3.07	#N/A	ALIS1_ARATH	7.10E-28
DN16176_c0_g2_i1	#N/A	#N/A	-0.85	PWD_ARATH	0
DN16178_c0_g2_i1	#N/A	-1.25	#N/A	.	#N/A
DN16189_c0_g1_i2	#N/A	#N/A	0.93	RS4_SOLTU	0
DN16189_c0_g2_i11	#N/A	#N/A	0.88	RS41_ARATH	0
DN16189_c0_g2_i12	#N/A	#N/A	0.76	RS42_ARATH	0
DN16189_c0_g2_i2	#N/A	#N/A	0.67	RS41_ARATH	0
DN16189_c0_g2_i5	#N/A	#N/A	0.89	RS42_ARATH	0
DN16192_c0_g2_i7	#N/A	#N/A	1.11	ACP1_CASGL	8.40E-56
DN16203_c1_g7_i3	#N/A	1.85	-2.26	HSP72_SOLLC	0
DN16203_c1_g7_i4	#N/A	0.74	#N/A	HSP7C_PETHY	6.00E-173
DN16209_c0_g1_i1	#N/A	4.84	#N/A	Y3720_ARATH	1.90E-20
DN16209_c0_g1_i4	#N/A	2.76	#N/A	Y3720_ARATH	0.002
DN16209_c0_g1_i5	#N/A	#N/A	1.02	.	#N/A
DN16212_c0_g1_i1	#N/A	#N/A	0.94	RS101_ARATH	8.70E-79
DN16212_c0_g1_i3	#N/A	#N/A	0.94	RS101_ARATH	3.70E-79
DN16212_c0_g2_i2	#N/A	#N/A	0.72	RS10B_ORYSJ	2.70E-80
DN16212_c0_g2_i5	#N/A	#N/A	0.75	RS10B_ORYSJ	1.10E-80
DN16215_c1_g1_i4	#N/A	#N/A	-7.63	SYT5_ARATH	0
DN16215_c1_g3_i1	#N/A	1.65	#N/A	C3H29_ARATH	2.00E-172
DN16215_c1_g3_i2	#N/A	1.42	-1.36	C3H29_ARATH	4.00E-175
DN16215_c1_g3_i5	#N/A	#N/A	-1.37	C3H29_ARATH	5.00E-153
DN16224_c0_g1_i1	#N/A	#N/A	0.66	RGGB_ARATH	9.90E-60
DN16224_c0_g1_i2	#N/A	#N/A	0.66	RGGB_ARATH	5.80E-60
DN16225_c0_g1_i4	#N/A	#N/A	0.9	RL182_ARATH	2.00E-117
DN16229_c0_g3_i8	#N/A	1.82	#N/A	SAM22_SOYBN	6.00E-111

Isoform ID	Log_2(#60tr/nt)	Log_2(#20tr/nt)	Log_2(#60tr/#20t	Annotation_protein	e_value_annotation
DN16231_c0_g3_i3	#N/A	#N/A	-6.98	YPTM2_MAIZE	1.40E-37
DN16253_c0_g4_i5	#N/A	#N/A	8.71	ZIP11_ARATH	3.00E-52
DN16255_c0_g1_i17	#N/A	-0.62	#N/A	PIRL6_ARATH	3.00E-146
DN16257_c0_g1_i1	#N/A	2.3	#N/A	WDL7_ARATH	1.30E-16
DN16261_c0_g2_i2	#N/A	-0.78	#N/A	.	#N/A
DN16264_c1_g1_i12	#N/A	7.85	#N/A	HAT5_ARATH	4.50E-43
DN16270_c0_g1_i9	#N/A	8.36	#N/A	TCX3_ARATH	2.50E-57
DN16284_c2_g1_i8	#N/A	#N/A	0.71	RS5_CICAR	2.00E-137
DN16286_c0_g4_i2	#N/A	#N/A	0.97	.	#N/A
DN16288_c0_g2_i2	#N/A	-0.88	#N/A	F16P2_SOLTU	5.00E-154
DN16294_c0_g1_i16	#N/A	#N/A	0.95	.	#N/A
DN16296_c1_g1_i3	#N/A	#N/A	0.53	CP29B_ARATH	3.50E-82
DN16296_c1_g1_i5	#N/A	#N/A	0.62	ROC1_NICSY	2.10E-90
DN16296_c1_g1_i8	#N/A	#N/A	0.62	ROC2_NICPL	1.00E-93
DN16299_c0_g1_i11	#N/A	#N/A	0.57	RPE_SPIOL	4.00E-180
DN16303_c0_g1_i7	#N/A	-0.88	#N/A	YIDC_PROA2	3.2
DN16303_c0_g4_i5	#N/A	#N/A	0.77	.	#N/A
DN16306_c0_g1_i2	#N/A	#N/A	1.04	.	#N/A
DN16306_c0_g2_i3	#N/A	#N/A	-7.94	SPP1_TOBAC	0
DN16310_c0_g1_i12	#N/A	-8.44	#N/A	ARP2_ARATH	3.00E-171
DN16311_c0_g2_i4	#N/A	#N/A	0.97	RK12_SPIOL	1.40E-62
DN16311_c0_g4_i1	#N/A	#N/A	1.03	RK121_ARATH	1.20E-60
DN16318_c1_g1_i16	#N/A	#N/A	-1.05	SIGE_ARATH	0
DN16318_c1_g1_i4	#N/A	#N/A	-0.66	SIGE_ARATH	2.00E-159
DN16324_c0_g2_i4	#N/A	2.63	#N/A	ASPA_ARATH	2.00E-160
DN16326_c1_g1_i1	#N/A	#N/A	1.42	.	#N/A
DN16326_c1_g1_i3	#N/A	-1.17	1.62	.	#N/A
DN16330_c0_g1_i10	#N/A	-1.03	#N/A	AMY2_ARATH	0
DN16338_c0_g5_i4	#N/A	8.04	#N/A	PGLR4_ARATH	1.00E-125
DN16340_c2_g5_i3	#N/A	#N/A	0.67	.	#N/A
DN16341_c0_g1_i11	#N/A	2.64	#N/A	EXA1_ARATH	3.00E-163
DN16341_c0_g1_i12	#N/A	#N/A	-5.15	EXA1_ARATH	5.00E-126
DN16342_c2_g4_i4	#N/A	#N/A	-1.55	RPV1_VITRO	6.20E-40
DN16364_c0_g1_i2	#N/A	#N/A	0.61	IM30_PEA	0
DN16365_c0_g1_i10	#N/A	#N/A	0.62	CYB5E_ARATH	5.10E-83
DN16371_c2_g1_i3	#N/A	#N/A	-6.62	Y3913_ARATH	6.00E-30
DN16371_c2_g4_i2	#N/A	#N/A	0.64	SCP2_ARATH	6.60E-70
DN16381_c0_g1_i5	#N/A	-8.99	#N/A	PSA4A_ARATH	5.30E-61
DN16383_c0_g2_i3	#N/A	#N/A	1.06	NH2L1_XENLA	2.80E-73

Isoform ID	Log_2(#60tr/nt)	Log_2(#20tr/nt)	Log_2(#60tr/#20t	Annotation_protein	e_value_annotation
DN16383_c0_g4_i1	#N/A	#N/A	0.77	NH2L1_XENLA	8.20E-73
DN16383_c0_g4_i2	#N/A	#N/A	0.75	NH2L1_XENLA	4.50E-73
DN16387_c0_g1_i2	#N/A	#N/A	-5.62	SNAA_VITVI	2.00E-123
DN16391_c0_g2_i10	#N/A	#N/A	0.79	.	#N/A
DN16393_c0_g1_i22	#N/A	#N/A	-0.89	HCAR_ARATH	2.00E-112
DN16394_c0_g3_i22	#N/A	7.85	#N/A	YIDE_SCHPO	2.40E-36
DN16394_c0_g3_i23	#N/A	#N/A	-7.63	YIDE_SCHPO	1.50E-09
DN16402_c1_g1_i11	#N/A	#N/A	-1.87	XG113_ARATH	4.00E-170
DN16415_c0_g3_i5	#N/A	7.72	#N/A	E131_ARATH	2.70E-26
DN16424_c0_g1_i2	#N/A	-2.41	#N/A	CO9_TAKRU	0.84
DN16424_c0_g3_i14	#N/A	#N/A	7.77	BH113_ARATH	8.60E-45
DN16426_c0_g2_i14	#N/A	#N/A	0.78	RL182_ARATH	4.00E-118
DN16426_c0_g2_i17	#N/A	#N/A	0.88	RL182_ARATH	6.00E-118
DN16426_c0_g2_i7	#N/A	#N/A	0.8	RL183_ARATH	2.80E-81
DN16426_c0_g2_i8	#N/A	#N/A	0.83	RL182_ARATH	9.00E-99
DN16426_c0_g2_i9	#N/A	#N/A	0.84	RL183_ARATH	2.00E-116
DN16428_c0_g2_i11	#N/A	#N/A	0.98	ISS1_ARATH	0
DN16436_c0_g1_i3	#N/A	#N/A	0.59	CP31B_ARATH	8.00E-105
DN16436_c0_g1_i5	#N/A	#N/A	0.86	CP31A_ARATH	2.00E-101
DN16436_c0_g1_i6	#N/A	#N/A	1.12	CP31B_ARATH	3.00E-104
DN16442_c1_g3_i5	#N/A	1.19	#N/A	EPAS1_RAT	0.2
DN16445_c0_g1_i7	#N/A	7.99	#N/A	LUT1_ARATH	2.00E-137
DN16452_c0_g1_i5	#N/A	#N/A	0.96	ASPGB_ARATH	0
DN16459_c0_g1_i1	#N/A	-0.71	#N/A	CF136_BOVIN	4.20E-06
DN16459_c0_g2_i7	#N/A	#N/A	0.62	ATPG_PEA	0
DN16467_c0_g2_i14	#N/A	#N/A	-8.23	QKIL3_ARATH	6.00E-151
DN16487_c0_g1_i4	#N/A	#N/A	0.76	THRC_SOLTU	0
DN16507_c0_g6_i1	#N/A	1.07	#N/A	.	#N/A
DN16507_c0_g6_i4	#N/A	0.62	#N/A	.	#N/A
DN16511_c0_g3_i3	#N/A	#N/A	1.9	PPCE_MOUSE	9.00E-135
DN16525_c1_g2_i9	#N/A	#N/A	-1.77	RGA4_SOLBU	1.10E-80
DN16528_c0_g1_i6	#N/A	8.14	#N/A	EMF2B_ORYSJ	9.90E-84
DN16533_c0_g2_i2	#N/A	-9.82	10.04	Y1049_ARATH	1.00E-163
DN16533_c0_g2_i6	#N/A	#N/A	-9.36	Y1049_ARATH	1.00E-163
DN16549_c0_g4_i3	#N/A	-10.12	#N/A	PTH2_BOVIN	1.20E-34
DN16558_c0_g1_i11	#N/A	#N/A	-1.28	.	#N/A
DN16574_c0_g4_i1	#N/A	4.44	-3.03	ACCR4_ARATH	0
DN16585_c0_g1_i12	#N/A	#N/A	-8.09	PITC_DICDI	1.10E-33
DN16591_c0_g9_i1	#N/A	#N/A	0.57	.	#N/A

Isoform ID	Log_2(#60tr/nt)	Log_2(#20tr/nt)	Log_2(#60tr/#20t	Annotation_protein	e_value_annotation
DN16593_c0_g2_i2	#N/A	#N/A	-4.9	MBD7_ARATH	1.60E-12
DN16607_c0_g2_i1	#N/A	#N/A	0.88	R27A3_ARATH	7.50E-90
DN16607_c0_g2_i2	#N/A	#N/A	1.44	R27A3_ARATH	7.50E-90
DN16607_c0_g3_i1	#N/A	#N/A	1.1	.	#N/A
DN16607_c0_g3_i3	#N/A	#N/A	1.05	.	#N/A
DN16607_c0_g3_i5	#N/A	#N/A	0.92	.	#N/A
DN16607_c0_g4_i1	#N/A	#N/A	0.95	.	#N/A
DN16607_c0_g4_i3	#N/A	#N/A	2.18	.	#N/A
DN16607_c0_g5_i1	#N/A	#N/A	0.98	.	#N/A
DN16610_c0_g4_i1	#N/A	#N/A	1.46	.	#N/A
DN16618_c0_g1_i17	#N/A	#N/A	7.55	PSDE_ARATH	0
DN16619_c0_g1_i2	#N/A	1.64	#N/A	HFB2B_ARATH	3.30E-97
DN16634_c0_g3_i7	#N/A	7.51	#N/A	.	#N/A
DN16641_c0_g1_i5	#N/A	#N/A	0.65	ADDA_LACBA	0.069
DN16658_c1_g1_i6	#N/A	#N/A	-7.94	.	#N/A
DN16659_c0_g1_i15	#N/A	#N/A	8.07	PFBS_ARATH	1.80E-43
DN16659_c0_g1_i17	#N/A	#N/A	-8.46	.	#N/A
DN16665_c0_g1_i10	#N/A	#N/A	0.81	RL24_PRUAV	3.40E-89
DN16665_c0_g1_i12	#N/A	#N/A	0.85	RL24_PRUAV	1.20E-87
DN16676_c0_g4_i5	#N/A	#N/A	0.59	RK1_ARATH	8.00E-158
DN16680_c0_g5_i4	#N/A	#N/A	0.66	FAP1_ARATH	2.00E-103
DN16683_c0_g1_i3	#N/A	8.52	-8.65	SRC30_ARATH	1.40E-45
DN16685_c0_g1_i21	#N/A	#N/A	0.58	SR45A_ARATH	2.40E-43
DN16692_c0_g3_i5	#N/A	-0.63	#N/A	SODC2_MESCR	1.90E-84
DN16697_c1_g2_i16	#N/A	3.57	#N/A	RIPK_ARATH	0
DN16699_c0_g1_i14	#N/A	#N/A	7.93	OFT15_ARATH	2.40E-75
DN16704_c0_g1_i1	#N/A	-8.52	#N/A	KASC1_ARATH	0
DN16705_c0_g1_i34	#N/A	1.17	#N/A	.	#N/A
DN16705_c0_g3_i4	#N/A	#N/A	0.66	RR20_SPIOL	2.00E-61
DN16705_c0_g8_i1	#N/A	#N/A	0.59	RR20_SPIOL	3.60E-61
DN16708_c0_g3_i5	#N/A	-1.69	1.72	RF3_DESAG	7.5
DN16714_c1_g6_i4	#N/A	0.6	#N/A	NCL_ARATH	0
DN16720_c0_g1_i5	#N/A	#N/A	1.09	ULP1_YEAST	5.3
DN16720_c0_g1_i6	#N/A	#N/A	1.04	R13A4_ARATH	4.00E-138
DN16720_c0_g6_i2	#N/A	#N/A	0.83	R13A4_ARATH	1.80E-98
DN16724_c0_g1_i18	#N/A	#N/A	-2.03	SARD1_ARATH	7.00E-134
DN16724_c0_g1_i3	#N/A	2.08	#N/A	SARD1_ARATH	9.00E-131
DN16734_c0_g1_i12	#N/A	-2.18	#N/A	YAI5_SCHPO	2.80E-15
DN16736_c0_g4_i1	#N/A	#N/A	0.84	DGAT3_ARATH	9.60E-38

Isoform ID	Log_2(#60tr/nt)	Log_2(#20tr/nt)	Log_2(#60tr/#20t	Annotation_protein	e_value_annotation
DN16744_c0_g2_i1	#N/A	#N/A	1.2	SCP51_ARATH	0
DN16767_c0_g1_i18	#N/A	-1.26	#N/A	TSJT1_TOBAC	2.30E-36
DN16767_c0_g2_i10	#N/A	-1.04	#N/A	TSJT1_TOBAC	2.50E-37
DN16768_c0_g2_i18	#N/A	-7.34	#N/A	HOX27_ORYSI	1.40E-22
DN16770_c0_g1_i15	#N/A	#N/A	-1.05	YC22_ARATH	0
DN16782_c0_g1_i13	#N/A	#N/A	-1.32	.	#N/A
DN16787_c2_g1_i14	#N/A	#N/A	-2.48	PEPD_HUMAN	2.00E-175
DN16789_c0_g3_i8	#N/A	#N/A	-7.54	MAIL2_ARATH	3.00E-16
DN16799_c0_g1_i7	#N/A	#N/A	-1.63	AQR_MOUSE	2.00E-123
DN16807_c0_g2_i5	#N/A	#N/A	0.65	MIFH_TRISP	2.20E-19
DN16810_c0_g2_i3	#N/A	-0.77	#N/A	.	#N/A
DN16811_c0_g1_i30	#N/A	#N/A	-8.27	ORP4B_ARATH	1.00E-139
DN16814_c1_g1_i6	#N/A	#N/A	0.71	PRSP1_SPIOL	1.00E-117
DN16816_c0_g2_i18	#N/A	6.27	#N/A	TAP46_TOBAC	5.00E-102
DN16819_c0_g1_i24	#N/A	#N/A	9.17	.	#N/A
DN16827_c0_g4_i1	#N/A	-0.84	#N/A	U74G1_STERE	4.00E-157
DN16830_c0_g1_i11	#N/A	-0.77	#N/A	ACCH4_ARATH	7.00E-107
DN16831_c0_g2_i4	#N/A	-0.84	#N/A	CCA_THEGJ	2.3
DN16834_c0_g1_i12	#N/A	-1.04	#N/A	UMPS_ARATH	0
DN16837_c0_g2_i5	#N/A	-0.88	#N/A	PSUG_SACEN	0.72
DN16841_c0_g3_i1	#N/A	#N/A	-1.17	CSLE1_ARATH	3.00E-123
DN16848_c0_g1_i17	#N/A	#N/A	1.08	.	#N/A
DN16848_c0_g1_i4	#N/A	#N/A	0.89	.	#N/A
DN16854_c0_g1_i11	#N/A	-7.39	#N/A	FAX3_ARATH	4.90E-70
DN16855_c0_g2_i2	#N/A	-0.73	#N/A	.	#N/A
DN16869_c0_g2_i10	#N/A	#N/A	0.64	NACA2_ARATH	9.50E-80
DN16869_c0_g2_i11	#N/A	#N/A	0.64	NACA2_ARATH	3.60E-75
DN16869_c0_g2_i15	#N/A	#N/A	0.73	NACA2_ARATH	3.40E-80
DN16875_c1_g1_i6	#N/A	#N/A	0.54	APX3_ARATH	6.00E-160
DN16876_c0_g1_i6	#N/A	#N/A	0.6	GRP1_SINAL	7.90E-43
DN16876_c0_g2_i3	#N/A	#N/A	0.86	.	#N/A
DN16884_c0_g3_i13	#N/A	-7.67	#N/A	.	#N/A
DN16884_c0_g3_i18	#N/A	7.68	#N/A	.	#N/A
DN16888_c0_g4_i6	#N/A	#N/A	0.6	KAD4_ORYSJ	2.00E-167
DN16894_c0_g1_i2	#N/A	-1.1	#N/A	AHL14_ARATH	1.10E-85
DN16895_c0_g1_i7	#N/A	#N/A	2.42	RUAP_SOYBN	0
DN16901_c0_g2_i1	#N/A	-5.09	#N/A	MAPC2_ARATH	0
DN16901_c0_g2_i13	#N/A	8.66	#N/A	KOG1_YEAST	3.1
DN16909_c0_g1_i14	#N/A	#N/A	-2.51	CC115_BOVIN	1.40E-07

Isoform ID	Log_2(#60tr/nt)	Log_2(#20tr/nt)	Log_2(#60tr/#20t	Annotation_protein	e_value_annotation
DN16911_c0_g4_i3	#N/A	#N/A	0.8	DML_EUBBA	5.90E-69
DN16933_c0_g1_i9	#N/A	#N/A	1.01	BAM1_ARATH	0
DN16944_c0_g1_i3	#N/A	8	#N/A	CB069_MOUSE	5.50E-05
DN16946_c0_g1_i6	#N/A	#N/A	8.77	BIM1_ARATH	3.00E-124
DN16949_c2_g2_i13	#N/A	2.03	#N/A	RM04_VANPO	0.54
DN16949_c2_g2_i2	#N/A	2.16	#N/A	.	#N/A
DN16949_c2_g2_i8	#N/A	2.57	#N/A	.	#N/A
DN16949_c2_g2_i9	#N/A	1.99	#N/A	EXTN_DAUCA	6.5
DN16965_c0_g1_i18	#N/A	8.14	#N/A	PAP7_ARATH	4.30E-60
DN16970_c0_g1_i1	#N/A	7.27	#N/A	ITN1_ARATH	0
DN16970_c0_g1_i5	#N/A	#N/A	-0.86	ITN1_ARATH	0
DN16972_c0_g1_i5	#N/A	1.4	#N/A	MFSD5_HUMAN	4.90E-73
DN16982_c0_g1_i7	#N/A	#N/A	-7.8	BPA1_ARATH	6.20E-24
DN16990_c0_g2_i8	#N/A	1.38	#N/A	RBL19_ARATH	2.00E-150
DN16990_c0_g3_i1	#N/A	#N/A	7.85	RBL19_ARATH	3.70E-86
DN17001_c0_g5_i3	#N/A	#N/A	1.06	RT27_YEAST	4.60E-08
DN17002_c1_g2_i10	#N/A	#N/A	9.28	ARP8_ARATH	1.00E-176
DN17014_c0_g3_i7	#N/A	#N/A	-8.24	LTPG2_ARATH	3.20E-28
DN17029_c0_g1_i4	#N/A	-1.08	#N/A	STR15_ARATH	1.60E-57
DN17029_c0_g1_i5	#N/A	-1.03	#N/A	STR15_ARATH	6.10E-58
DN17030_c0_g3_i4	#N/A	#N/A	-0.74	AB39G_ARATH	0
DN17033_c0_g2_i13	#N/A	#N/A	0.59	PST2_GOSHI	4.70E-31
DN17033_c0_g2_i7	#N/A	#N/A	0.6	PST2_GOSHI	1.60E-27
DN17035_c1_g1_i6	#N/A	#N/A	-1.26	SG101_ARATH	1.50E-89
DN17037_c0_g1_i10	#N/A	#N/A	9.56	.	#N/A
DN17039_c0_g2_i1	#N/A	-0.91	#N/A	BRG1_ARATH	3.80E-70
DN17041_c0_g1_i1	#N/A	#N/A	1.74	CH20_ARATH	4.00E-127
DN17041_c0_g1_i7	#N/A	#N/A	1.59	CH20_ARATH	5.00E-128
DN17041_c0_g2_i1	#N/A	#N/A	0.8	CH20_ARATH	9.00E-133
DN17041_c0_g2_i2	#N/A	#N/A	1.14	.	#N/A
DN17041_c0_g2_i5	#N/A	#N/A	0.74	CH20_ARATH	4.00E-133
DN17044_c0_g1_i6	#N/A	#N/A	0.61	CP19D_ARATH	3.00E-126
DN17053_c0_g1_i4	#N/A	-9.78	#N/A	PSMD6_ARATH	0
DN17053_c0_g1_i7	#N/A	#N/A	1.62	PSMD6_ARATH	0
DN17053_c0_g1_i9	#N/A	1	#N/A	PSMD6_ARATH	0
DN17054_c1_g1_i3	#N/A	#N/A	-0.7	FRI3_SOYBN	0
DN17062_c0_g4_i1	#N/A	1.74	#N/A	BAG5_ARATH	1.10E-09
DN17065_c0_g4_i1	#N/A	#N/A	0.8	.	#N/A
DN17065_c0_g4_i2	#N/A	#N/A	0.79	.	#N/A

Isoform ID	Log_2(#60tr/nt)	Log_2(#20tr/nt)	Log_2(#60tr/#20t	Annotation_protein	e_value_annotation
DN17068_c0_g1_i3	#N/A	-1.82	#N/A	PDXJ_SULSY	0.69
DN17069_c0_g1_i1	#N/A	#N/A	0.75	.	#N/A
DN17069_c0_g1_i5	#N/A	#N/A	0.83	.	#N/A
DN17069_c0_g3_i3	#N/A	#N/A	0.75	.	#N/A
DN17072_c0_g1_i1	#N/A	#N/A	1.1	.	#N/A
DN17075_c0_g2_i1	#N/A	#N/A	0.81	RL10_VITRI	2.00E-155
DN17075_c0_g3_i1	#N/A	#N/A	1.02	RL10_VITRI	6.60E-80
DN17075_c0_g3_i2	#N/A	#N/A	0.71	RL10_VITRI	5.70E-79
DN17076_c0_g3_i9	#N/A	#N/A	0.75	YEEZ_ECOLI	0.00057
DN17077_c1_g1_i3	#N/A	7.48	#N/A	FB345_ARATH	9.10E-97
DN17087_c0_g1_i6	#N/A	-0.75	#N/A	HMGL_IPONI	2.70E-50
DN17089_c0_g3_i9	#N/A	-5.11	5.9	TRXM1_ARATH	9.70E-27
DN17092_c0_g2_i6	#N/A	#N/A	8.65	HIBC8_ARATH	2.00E-144
DN17094_c0_g1_i5	#N/A	#N/A	-0.98	GT14A_ARATH	1.00E-151
DN17096_c0_g1_i5	#N/A	7.49	-7.62	PPP5_SOLLC	8.00E-139
DN17101_c0_g2_i4	#N/A	#N/A	0.76	MTEF1_ARATH	2.00E-66
DN17104_c0_g3_i4	#N/A	#N/A	-3.07	WRK70_ARATH	1.80E-23
DN17107_c0_g3_i3	#N/A	-4.15	#N/A	TM39B_DANRE	9.2
DN17109_c1_g1_i27	#N/A	#N/A	-0.7	NHX2_ARATH	0
DN17109_c1_g1_i6	#N/A	#N/A	-8.64	NHX2_ARATH	0
DN17112_c0_g2_i9	#N/A	-0.84	#N/A	PEX13_ARATH	1.80E-55
DN17119_c0_g2_i1	#N/A	-5.14	5.08	CP2_ARATH	4.00E-115
DN17119_c0_g2_i4	#N/A	#N/A	-4.34	CP2_ARATH	5.00E-115
DN17128_c0_g1_i11	#N/A	#N/A	0.66	THI42_VITVI	0
DN17128_c0_g1_i2	#N/A	#N/A	0.84	THI42_VITVI	0
DN17128_c0_g1_i3	#N/A	#N/A	0.72	THI42_VITVI	0
DN17133_c0_g1_i1	#N/A	0.99	#N/A	.	#N/A
DN17136_c0_g3_i6	#N/A	#N/A	0.71	HP302_ARATH	5.00E-109
DN17139_c1_g2_i8	#N/A	#N/A	9.31	MAP1_MYCPN	2.9
DN17140_c0_g1_i11	#N/A	#N/A	1.09	RS6_ASPOF	3.00E-169
DN17140_c0_g1_i8	#N/A	#N/A	0.93	RS6_ASPOF	3.00E-168
DN17140_c0_g3_i1	#N/A	#N/A	0.9	RS6_ASPOF	4.90E-86
DN17141_c0_g1_i1	#N/A	#N/A	0.71	.	#N/A
DN17141_c0_g1_i3	#N/A	#N/A	0.73	.	#N/A
DN17142_c0_g1_i1	#N/A	#N/A	1.84	CCX1_ARATH	0
DN17144_c0_g1_i20	#N/A	-0.86	#N/A	.	#N/A
DN17145_c0_g3_i9	#N/A	#N/A	10.43	MRS2B_ARATH	6.00E-76
DN17153_c1_g2_i2	#N/A	#N/A	-1	CFM2_ARATH	0
DN17161_c0_g1_i3	#N/A	#N/A	-4.61	REV3L_MOUSE	2.7

Isoform ID	Log_2(#60tr/nt)	Log_2(#20tr/nt)	Log_2(#60tr/#20t	Annotation_protein	e_value_annotation
DN17166_c0_g1_i1	#N/A	2.63	#N/A	GDL48_ARATH	4.00E-143
DN17168_c0_g2_i14	#N/A	#N/A	0.54	.	#N/A
DN17168_c0_g2_i17	#N/A	#N/A	0.63	ARF1_ARATH	1.00E-120
DN17168_c0_g2_i5	#N/A	#N/A	0.67	ARF_VIGUN	3.30E-74
DN17170_c0_g1_i4	#N/A	-0.77	#N/A	PTC52_ARATH	0
DN17189_c0_g1_i4	#N/A	#N/A	1.57	BSPA_POPDE	4.10E-34
DN17189_c0_g1_i6	#N/A	#N/A	1.27	BSPA_POPDE	9.30E-34
DN17190_c0_g3_i1	#N/A	7.54	#N/A	TEN1_CAEEL	0.31
DN17195_c0_g1_i4	#N/A	0.81	#N/A	FD6E2_SOYBN	0
DN17195_c0_g1_i6	#N/A	#N/A	0.97	FD6E2_SOYBN	0
DN17196_c1_g2_i1	#N/A	-0.85	#N/A	PCP_PYRFU	9.10E-21
DN17199_c0_g1_i14	#N/A	1.19	-0.94	MGAT3_RAT	9.40E-17
DN17208_c0_g1_i8	#N/A	#N/A	0.67	WLIM1_ARATH	1.00E-117
DN17209_c0_g1_i4	#N/A	#N/A	1.56	PMT2_ARATH	0
DN17211_c0_g2_i3	#N/A	#N/A	0.64	GGR_ARATH	1.00E-136
DN17215_c0_g1_i1	#N/A	#N/A	-7.6	XCT_ARATH	9.00E-99
DN17216_c0_g1_i4	#N/A	1.38	#N/A	.	#N/A
DN17220_c0_g1_i11	#N/A	#N/A	-7.37	PPA27_ARATH	4.30E-35
DN17229_c0_g1_i8	#N/A	0.96	#N/A	Y5344_ARATH	2.00E-172
DN17233_c0_g1_i3	#N/A	1.29	#N/A	P2C25_ARATH	1.00E-164
DN17242_c0_g1_i11	#N/A	#N/A	0.84	RS33_ARATH	5.00E-150
DN17242_c0_g1_i17	#N/A	#N/A	1.14	RS33_ARATH	9.00E-150
DN17242_c0_g1_i2	#N/A	#N/A	0.82	RS33_ARATH	3.00E-145
DN17245_c0_g1_i11	#N/A	#N/A	0.55	HSPC2_RICFE	1.40E-09
DN17245_c0_g1_i16	#N/A	-10.85	10.84	HSPC2_RICFE	4.60E-11
DN17245_c0_g1_i7	#N/A	#N/A	0.81	HSPC2_RICFE	4.20E-15
DN17251_c0_g1_i2	#N/A	#N/A	0.9	RS7_AVIMR	5.00E-118
DN17251_c0_g1_i5	#N/A	#N/A	1.11	RS7_AVIMR	6.00E-118
DN17251_c0_g1_i7	#N/A	#N/A	0.95	RS7_AVIMR	2.00E-118
DN17251_c0_g1_i8	#N/A	#N/A	0.66	RS7_AVIMR	3.00E-118
DN17251_c0_g2_i6	#N/A	#N/A	0.74	RS7_AVIMR	3.00E-116
DN17257_c0_g2_i10	#N/A	#N/A	0.96	R15A1_ARATH	9.70E-92
DN17257_c0_g2_i13	#N/A	#N/A	1.16	R15A1_ARATH	9.70E-92
DN17257_c0_g2_i14	#N/A	#N/A	1.06	R15A1_ARATH	9.70E-92
DN17257_c0_g2_i6	#N/A	#N/A	0.94	.	#N/A
DN17257_c0_g2_i8	#N/A	#N/A	1.1	R15A1_ARATH	9.70E-92
DN17257_c0_g2_i9	#N/A	#N/A	0.72	R15A1_ARATH	9.70E-92
DN17259_c0_g1_i2	#N/A	1.66	-1.54	5MMP_ARATH	1.40E-38
DN17259_c0_g1_i7	#N/A	3.79	#N/A	5MMP_ARATH	3.90E-41

Isoform ID	Log_2(#60tr/nt)	Log_2(#20tr/nt)	Log_2(#60tr/#20t	Annotation_protein	e_value_annotation
DN17277_c0_g2_i9	#N/A	#N/A	0.74	IF4E2_WHEAT	2.10E-98
DN17285_c1_g1_i4	#N/A	-8.04	#N/A	RNP1_ARATH	9.80E-34
DN17291_c0_g2_i1	#N/A	#N/A	0.91	FABD_HUMAN	9.70E-55
DN17298_c0_g1_i13	#N/A	#N/A	0.75	CHLI_SOYBN	0
DN17298_c0_g1_i3	#N/A	#N/A	0.72	CHLI_SOYBN	0
DN17298_c0_g1_i8	#N/A	#N/A	0.85	CHLI_SOYBN	0
DN17306_c1_g4_i3	#N/A	#N/A	-0.95	HS22C_SOYBN	1.00E-126
DN17313_c0_g1_i4	#N/A	-0.9	0.69	MDHG_CUCSA	3.90E-53
DN17316_c0_g1_i4	#N/A	#N/A	-9.44	SELO_METFK	3.00E-140
DN17316_c0_g1_i7	#N/A	#N/A	-12.03	SELO_AROAE	1.00E-167
DN17318_c0_g3_i3	#N/A	#N/A	5.02	CAP16_ARATH	5.40E-55
DN17318_c0_g4_i2	#N/A	#N/A	0.9	.	#N/A
DN17320_c0_g3_i1	#N/A	#N/A	0.66	.	#N/A
DN17334_c1_g1_i24	#N/A	5.2	#N/A	C719E_ARGME	1.6
DN17340_c1_g4_i2	#N/A	-1.21	#N/A	.	#N/A
DN17341_c0_g1_i5	#N/A	#N/A	-7.4	NSRR_BACLD	8.2
DN17342_c1_g1_i1	#N/A	-1.12	#N/A	SNAT2_ORYSJ	1.80E-64
DN17345_c1_g1_i12	#N/A	#N/A	0.72	NUCL2_ORYSJ	1.10E-82
DN17346_c0_g2_i2	#N/A	#N/A	0.86	RL3_ORYSJ	0
DN17346_c0_g2_i6	#N/A	#N/A	1.05	RL3_ORYSJ	0
DN17355_c0_g2_i4	#N/A	#N/A	0.88	PMTF_ARATH	0
DN17361_c0_g1_i10	#N/A	#N/A	-1.99	PIF7_ARATH	1.00E-39
DN17362_c0_g1_i12	#N/A	#N/A	1.67	PMAT1_ARATH	2.50E-21
DN17367_c0_g2_i1	#N/A	#N/A	0.87	HDT1_SOYBN	1.60E-96
DN17367_c0_g2_i2	#N/A	#N/A	0.75	HDT1_SOYBN	8.00E-107
DN17368_c0_g3_i9	#N/A	-0.89	#N/A	.	#N/A
DN17370_c0_g2_i4	#N/A	#N/A	-8.65	.	#N/A
DN17370_c0_g3_i13	#N/A	#N/A	2.72	CER1_ARATH	0
DN17370_c0_g3_i7	#N/A	10.79	#N/A	CER1_ARATH	0
DN17383_c0_g1_i22	#N/A	#N/A	-1.02	RSMH_HERAR	0.38
DN17383_c0_g1_i7	#N/A	#N/A	1.55	RSMH_HERAR	0.38
DN17384_c1_g4_i1	#N/A	8.86	#N/A	GID1B_ARATH	0
DN17389_c0_g1_i8	#N/A	1	#N/A	EXOL5_ARATH	5.00E-173
DN17391_c1_g2_i5	#N/A	#N/A	-7.77	CB60B_ARATH	5.50E-54
DN17392_c0_g2_i3	#N/A	8.14	#N/A	PGA2_SCHPO	1.4
DN17397_c0_g2_i7	#N/A	2.97	-4.32	BH063_ARATH	2.80E-55
DN17401_c0_g2_i2	#N/A	#N/A	2.14	TCP5_ARATH	6.70E-47
DN17414_c0_g2_i1	#N/A	#N/A	5.84	.	#N/A
DN17429_c0_g2_i1	#N/A	-1.28	1.56	PSBO_PEA	0

Isoform ID	Log_2(#60tr/nt)	Log_2(#20tr/nt)	Log_2(#60tr/#20t	Annotation_protein	e_value_annotation
DN17429_c0_g2_i2	#N/A	#N/A	0.61	PSBO_PEA	0
DN17429_c0_g2_i3	#N/A	#N/A	0.6	PSBO_PEA	0
DN17429_c0_g2_i4	#N/A	#N/A	1.08	PSBO_PEA	0
DN17429_c0_g2_i6	#N/A	#N/A	0.66	PSBO_PEA	0
DN17435_c0_g1_i3	#N/A	#N/A	1.69	MARF1_XENTR	1.50E-06
DN17439_c0_g1_i5	#N/A	#N/A	0.72	ADT3_ARATH	3.00E-154
DN17439_c0_g3_i1	#N/A	#N/A	0.64	ADT3_ARATH	2.00E-104
DN17444_c1_g6_i1	#N/A	8.09	#N/A	.	#N/A
DN17452_c0_g1_i5	#N/A	#N/A	-1.15	PCAP1_ARATH	5.20E-53
DN17452_c0_g1_i6	#N/A	#N/A	-1.47	PCAP1_ARATH	1.50E-52
DN17454_c1_g1_i7	#N/A	7.96	#N/A	P2C10_ARATH	1.00E-113
DN17469_c0_g1_i3	#N/A	1.45	#N/A	.	#N/A
DN17475_c0_g1_i20	#N/A	#N/A	-7.65	.	#N/A
DN17477_c1_g1_i22	#N/A	#N/A	10.26	XRI1_ARATH	5.60E-80
DN17478_c0_g7_i2	#N/A	#N/A	0.85	PTR18_ARATH	0
DN17481_c0_g2_i7	#N/A	4.89	#N/A	AUXI1_ARATH	3.00E-99
DN17514_c1_g1_i11	#N/A	7.57	#N/A	FBD14_ARATH	7.80E-18
DN17516_c0_g1_i6	#N/A	#N/A	1.32	IPYR_GLUOX	0.89
DN17517_c0_g2_i7	#N/A	#N/A	-5.14	PSI1_ARATH	7.20E-69
DN17522_c0_g4_i3	#N/A	-3.05	#N/A	AGL82_ARATH	4.40E-11
DN17523_c1_g1_i10	#N/A	-0.71	#N/A	H2B1_MEDTR	3.90E-88
DN17523_c1_g1_i12	#N/A	-0.64	#N/A	H2B1_MEDTR	3.90E-88
DN17526_c0_g2_i13	#N/A	1.36	#N/A	ANR52_CHICK	4.90E-08
DN17529_c0_g2_i5	#N/A	#N/A	0.59	.	#N/A
DN17538_c0_g2_i10	#N/A	#N/A	0.6	YCF23_PORPU	1.10E-58
DN17543_c0_g1_i9	#N/A	#N/A	0.8	RM24_MOUSE	7.40E-25
DN17561_c0_g1_i10	#N/A	-0.8	#N/A	P2C73_ARATH	5.00E-163
DN17561_c0_g1_i13	#N/A	-0.79	#N/A	P2C73_ARATH	9.00E-164
DN17561_c0_g1_i7	#N/A	-0.95	#N/A	P2C34_ARATH	3.40E-47
DN17563_c1_g1_i17	#N/A	#N/A	0.87	ODO2B_ARATH	0
DN17564_c2_g1_i14	#N/A	#N/A	1.07	.	#N/A
DN17564_c2_g1_i18	#N/A	#N/A	0.74	.	#N/A
DN17565_c0_g1_i3	#N/A	#N/A	7.98	S2544_HUMAN	8.00E-41
DN17570_c0_g1_i1	#N/A	#N/A	0.8	1433B_SOYBN	2.00E-178
DN17570_c0_g1_i4	#N/A	#N/A	1.23	1433B_SOYBN	2.00E-138
DN17571_c1_g2_i1	#N/A	#N/A	-7.17	.	#N/A
DN17573_c0_g1_i1	#N/A	#N/A	0.54	AAPC_CENCI	5.50E-37
DN17579_c0_g1_i4	#N/A	1.11	#N/A	ACT3_PEA	0
DN17581_c0_g1_i1	#N/A	#N/A	0.77	PSBX_SYNJB	4.80E-05

Isoform ID	Log_2(#60tr/nt)	Log_2(#20tr/nt)	Log_2(#60tr/#20t	Annotation_protein	e_value_annotation
DN17581_c0_g1_i3	#N/A	#N/A	0.76	PSBX_SYNJB	1.40E-05
DN17581_c0_g1_i5	#N/A	#N/A	0.8	PSBX_SYNJB	2.50E-05
DN17587_c0_g1_i16	#N/A	-8.67	#N/A	MVD2_ARATH	4.20E-38
DN17588_c0_g1_i26	#N/A	#N/A	-0.81	AKR1_SOYBN	0
DN17588_c0_g1_i9	#N/A	#N/A	-1.3	AKR1_SOYBN	1.70E-98
DN17588_c0_g2_i3	#N/A	#N/A	0.52	.	#N/A
DN1759_c0_g1_i1	#N/A	#N/A	0.68	.	#N/A
DN17590_c0_g1_i2	#N/A	-1.25	1.62	IPN2_LOTJA	3.40E-87
DN17602_c0_g1_i10	#N/A	#N/A	0.9	.	#N/A
DN17602_c0_g1_i16	#N/A	#N/A	1.02	CINAL_SYNP6	4.4
DN17602_c0_g1_i3	#N/A	#N/A	0.74	.	#N/A
DN17602_c0_g1_i4	#N/A	#N/A	0.97	.	#N/A
DN17602_c0_g1_i9	#N/A	#N/A	0.96	.	#N/A
DN17622_c0_g1_i11	#N/A	-2.39	2.5	Y2452_ARATH	0
DN17627_c0_g2_i1	#N/A	#N/A	0.9	TPIC_ARATH	8.30E-82
DN17635_c1_g5_i1	#N/A	#N/A	1.66	YAER_ECOLI	0.0005
DN17635_c1_g5_i2	#N/A	#N/A	1.54	LGUL_MOUSE	0.0002
DN17638_c0_g2_i2	#N/A	#N/A	10.68	HT1_ARATH	2.00E-79
DN17639_c1_g2_i11	#N/A	4.87	#N/A	.	#N/A
DN17640_c1_g2_i5	#N/A	2.04	#N/A	PRIL_METBU	0.07
DN17642_c0_g1_i5	#N/A	#N/A	-8.82	E135_ARATH	1.00E-160
DN17650_c0_g1_i16	#N/A	#N/A	1.04	HIS1B_ARATH	0
DN17651_c0_g2_i3	#N/A	#N/A	0.71	RER4_ARATH	8.20E-44
DN17669_c2_g6_i2	#N/A	#N/A	0.76	OTC_PEA	0
DN17678_c1_g1_i1	#N/A	#N/A	0.82	RBS1_SOYBN	1.00E-133
DN17678_c1_g1_i10	#N/A	#N/A	1.14	RBS1_SOYBN	1.00E-133
DN17678_c1_g1_i4	#N/A	#N/A	1.04	RBS1_SOYBN	4.60E-53
DN17678_c1_g1_i7	#N/A	#N/A	0.75	.	#N/A
DN17678_c1_g1_i9	#N/A	#N/A	0.71	RBS1_SOYBN	3.50E-70
DN17687_c0_g1_i15	#N/A	#N/A	1.03	CP122_ARATH	1.20E-42
DN17687_c0_g1_i3	#N/A	#N/A	0.83	CBG_MESAU	1.3
DN17687_c0_g1_i4	#N/A	-1.06	0.8	CP122_ARATH	1.20E-42
DN17687_c0_g1_i7	#N/A	#N/A	0.61	.	#N/A
DN17690_c1_g1_i16	#N/A	#N/A	0.77	PTAC5_ARATH	1.00E-126
DN17708_c0_g1_i1	#N/A	#N/A	-7.96	WRK41_ARATH	9.30E-08
DN17708_c0_g1_i2	#N/A	3.06	-2.12	WRK41_ARATH	6.30E-72
DN17708_c0_g1_i3	#N/A	3.48	-2.43	WRK41_ARATH	1.80E-73
DN17712_c1_g3_i2	#N/A	#N/A	2.74	PUB13_ARATH	1.40E-85
DN17716_c0_g1_i11	#N/A	5.22	#N/A	KDSB_ARATH	5.00E-175

Isoform ID	Log_2(#60tr/nt)	Log_2(#20tr/nt)	Log_2(#60tr/#20t	Annotation_protein	e_value_annotation
DN17717_c0_g1_i5	#N/A	#N/A	-7.4	TRP4_ARATH	0.00071
DN17722_c0_g1_i15	#N/A	#N/A	3.73	NIPA6_ARATH	1.00E-117
DN17722_c0_g1_i19	#N/A	#N/A	1.82	ATP6_LOXAF	3.9
DN17724_c2_g1_i2	#N/A	#N/A	1.12	.	#N/A
DN17724_c2_g1_i4	#N/A	#N/A	1.06	RL44_GOSHI	1.30E-69
DN17735_c0_g1_i17	#N/A	#N/A	6.14	BZP23_ARATH	3.00E-110
DN17740_c0_g2_i2	#N/A	#N/A	0.65	RL61_ARATH	1.40E-93
DN17740_c0_g3_i2	#N/A	#N/A	0.68	RL6_MESCR	3.00E-128
DN17754_c0_g4_i3	#N/A	#N/A	-2.7	RNHX1_ARATH	0.004
DN17756_c0_g1_i7	#N/A	8.18	#N/A	HPR3_ARATH	1.50E-14
DN17758_c0_g2_i9	#N/A	#N/A	9.06	HEMH_CUCSA	0
DN17769_c0_g1_i1	#N/A	#N/A	0.65	ACCC1_POPTR	0
DN17771_c0_g3_i1	#N/A	#N/A	0.67	PPD6_ARATH	2.00E-127
DN17771_c0_g3_i5	#N/A	-8.94	#N/A	PPD6_ARATH	2.00E-113
DN17775_c0_g2_i7	#N/A	-1.84	#N/A	NAP2_SOLLC	3.00E-115
DN17779_c0_g6_i2	#N/A	7.38	#N/A	FBK50_ARATH	5.50E-33
DN17795_c0_g2_i3	#N/A	1.85	#N/A	TCPD_ARATH	0
DN17801_c0_g1_i12	#N/A	#N/A	8.32	UBCP_ARATH	0
DN17803_c0_g3_i13	#N/A	7.69	#N/A	CKX7_ARATH	7.00E-108
DN17810_c1_g2_i1	#N/A	#N/A	0.61	FDC2_ARATH	3.10E-86
DN17810_c1_g2_i2	#N/A	#N/A	0.58	.	#N/A
DN17811_c0_g4_i1	#N/A	#N/A	1.03	RS191_ARATH	8.50E-92
DN17811_c0_g4_i2	#N/A	#N/A	0.93	RS191_ARATH	5.70E-92
DN17817_c0_g1_i25	#N/A	-0.75	#N/A	DNAJ_FINM2	2.00E-11
DN17817_c0_g1_i8	#N/A	-0.88	#N/A	DNAJ_FINM2	8.30E-11
DN17818_c0_g1_i10	#N/A	8.11	#N/A	PXN_ARATH	7.00E-139
DN17819_c0_g2_i3	#N/A	#N/A	-8.24	FENR2_PEA	0
DN17825_c0_g2_i3	#N/A	#N/A	0.86	THIC1_ARATH	0
DN17839_c0_g1_i10	#N/A	#N/A	0.92	.	#N/A
DN17841_c0_g3_i6	#N/A	#N/A	-1.26	CMTA4_ARATH	4.00E-108
DN17842_c0_g2_i5	#N/A	#N/A	-7.7	BAGP1_ARATH	0
DN17849_c1_g1_i5	#N/A	-2.22	#N/A	UBC22_ARATH	6.00E-134
DN17849_c1_g3_i3	#N/A	#N/A	-2.23	.	#N/A
DN17851_c0_g1_i7	#N/A	7.54	#N/A	Y5161_ARATH	1.50E-55
DN17855_c0_g1_i4	#N/A	#N/A	1	RS12_HORVU	1.10E-70
DN17855_c0_g1_i9	#N/A	#N/A	1.16	RS12_HORVU	5.40E-70
DN17857_c0_g1_i15	#N/A	-7.55	#N/A	CCR4E_ARATH	5.60E-49
DN17886_c0_g1_i19	#N/A	1.14	-1.16	ALFC5_ARATH	9.80E-72
DN17886_c0_g1_i22	#N/A	#N/A	-1.51	.	#N/A

Isoform ID	Log$_2$(#60tr/nt)	Log$_2$(#20tr/nt)	Log$_2$(#60tr/#20t	Annotation_protein	e_value_annotation
DN17886_c0_g1_i26	#N/A	#N/A	0.92	ALFC5_ARATH	1.20E-61
DN17888_c0_g1_i5	#N/A	5.95	#N/A	CIPK8_ARATH	0
DN17889_c0_g9_i1	#N/A	#N/A	1.36	5MAT_ARATH	1.00E-79
DN17898_c0_g1_i14	#N/A	#N/A	-1.07	ITN1_ARATH	1.50E-11
DN17901_c0_g1_i5	#N/A	0.63	#N/A	CSCLD_ARATH	0
DN17913_c0_g4_i17	#N/A	#N/A	-8.48	RPE1_ORYSJ	4.00E-111
DN17917_c0_g2_i10	#N/A	#N/A	1.04	.	#N/A
DN17917_c0_g2_i12	#N/A	#N/A	1.26	.	#N/A
DN17917_c0_g2_i7	#N/A	#N/A	1.06	.	#N/A
DN17917_c0_g2_i8	#N/A	#N/A	1.02	RS27A_LUPAL	9.40E-84
DN17926_c0_g1_i7	#N/A	#N/A	-7.01	MED27_ARATH	1.00E-170
DN17926_c0_g2_i3	#N/A	#N/A	8.65	GONS3_ARATH	0
DN17928_c1_g10_i1	#N/A	#N/A	0.81	PPA1_SOLLC	2.10E-86
DN17931_c0_g2_i8	#N/A	-0.78	0.92	BH144_ARATH	4.90E-39
DN17932_c0_g1_i15	#N/A	#N/A	8.03	PMK_ARATH	0
DN17939_c0_g1_i7	#N/A	#N/A	0.75	MGN2_ORYSJ	5.00E-99
DN17941_c0_g3_i6	#N/A	#N/A	-1.64	.	#N/A
DN17942_c0_g1_i1	#N/A	0.9	#N/A	GUN24_ORYSJ	0
DN17944_c0_g1_i7	#N/A	#N/A	-10.01	RBP45_NICPL	4.00E-161
DN17947_c0_g2_i15	#N/A	#N/A	-0.96	LAT4_BOVIN	1.3
DN17964_c0_g1_i1	#N/A	#N/A	0.7	RK4_TOBAC	2.10E-26
DN17964_c1_g1_i2	#N/A	#N/A	1.07	PAM68_ARATH	2.60E-57
DN17964_c1_g1_i6	#N/A	#N/A	1.1	PAM68_ARATH	9.50E-56
DN17968_c0_g1_i13	#N/A	#N/A	1.9	C90A1_ARATH	9.00E-163
DN17980_c0_g1_i12	#N/A	-9.69	#N/A	TIC56_ARATH	0
DN17981_c1_g1_i1	#N/A	1.58	#N/A	UGPI6_ARATH	0
DN17985_c0_g1_i1	#N/A	#N/A	-0.62	BIC1_ARATH	2.40E-33
DN17988_c0_g2_i1	#N/A	#N/A	-0.88	MPK3_ARATH	0
DN17999_c0_g1_i16	#N/A	#N/A	0.97	HEM3_PEA	0
DN18017_c0_g1_i6	#N/A	#N/A	-1.45	EDSBC_ARATH	1.00E-154
DN18018_c1_g1_i14	#N/A	#N/A	-0.74	AB22G_ARATH	0
DN18018_c1_g1_i6	#N/A	#N/A	-0.85	AB22G_ARATH	0
DN18020_c1_g1_i6	#N/A	#N/A	0.75	YCF54_PORPU	4.70E-12
DN18020_c1_g1_i9	#N/A	#N/A	0.79	YCF54_PORPU	7.60E-12
DN18020_c1_g3_i6	#N/A	-7.6	#N/A	GP_BUNGE	3.3
DN18024_c1_g3_i1	#N/A	#N/A	0.81	RL282_ARATH	8.90E-74
DN18024_c1_g3_i2	#N/A	#N/A	1.03	RL282_ARATH	1.20E-74
DN18024_c1_g6_i3	#N/A	#N/A	1.05	RL282_ARATH	5.30E-74
DN18024_c1_g6_i5	#N/A	#N/A	1.16	RL282_ARATH	3.20E-73

Isoform ID	Log_2(#60tr/nt)	Log_2(#20tr/nt)	Log_2(#60tr/#20t	Annotation_protein	e_value_annotation
DN18025_c0_g1_i2	#N/A	#N/A	8.19	URGT5_ARATH	0
DN18030_c0_g2_i2	#N/A	#N/A	1.33	MPCP3_ARATH	1.10E-73
DN18030_c0_g3_i2	#N/A	-0.84	#N/A	.	#N/A
DN18032_c0_g1_i2	#N/A	#N/A	-3.41	GAT22_ARATH	7.30E-35
DN18032_c0_g1_i8	#N/A	#N/A	-2.13	GAT22_ARATH	6.40E-37
DN18033_c0_g2_i10	#N/A	#N/A	-9.58	BCD1_MOUSE	1.50E-27
DN18039_c0_g1_i5	#N/A	#N/A	0.69	C3H30_ARATH	1.6
DN18051_c0_g1_i23	#N/A	8	#N/A	FLK_ARATH	2.90E-31
DN18052_c1_g1_i10	#N/A	8.24	#N/A	.	#N/A
DN18065_c1_g1_i4	#N/A	#N/A	-1.13	PHSL_VICFA	0
DN18068_c0_g5_i1	#N/A	-1.25	#N/A	RNY_FRACC	0.93
DN18068_c0_g7_i2	#N/A	-1.12	#N/A	.	#N/A
DN18084_c0_g7_i5	#N/A	#N/A	-1.66	9DC3_SOLPI	1.30E-77
DN18102_c0_g1_i8	#N/A	-3.54	#N/A	IDM3_ARATH	1.10E-36
DN18105_c0_g6_i6	#N/A	#N/A	9.2	NOE2_RAT	0.48
DN18127_c1_g1_i4	#N/A	#N/A	-3.9	SYS_SYNY3	3.4
DN18127_c1_g1_i9	#N/A	#N/A	-1.2	NUD2_CAEEL	3.1
DN18130_c0_g2_i2	#N/A	#N/A	1.92	.	#N/A
DN18130_c0_g2_i6	#N/A	#N/A	2.92	.	#N/A
DN18130_c0_g2_i7	#N/A	#N/A	1.53	DNAJ8_ARATH	3.50E-34
DN18131_c0_g1_i1	#N/A	1.05	#N/A	CESA9_ARATH	0
DN18131_c0_g3_i1	#N/A	#N/A	-9.23	APC4_ARATH	0
DN18132_c0_g1_i3	#N/A	#N/A	-1.3	SAC2_ARATH	2.00E-133
DN18133_c0_g1_i7	#N/A	1.02	#N/A	UGDH4_ORYSJ	0
DN18137_c0_g2_i6	#N/A	#N/A	0.79	NOTC4_HUMAN	0.011
DN18140_c3_g1_i1	#N/A	#N/A	1.63	CSLG2_ARATH	4.10E-88
DN18140_c3_g1_i2	#N/A	#N/A	2.28	CSLE2_ORYSJ	2.90E-20
DN18140_c3_g1_i3	#N/A	#N/A	1.52	CSLG2_ARATH	8.10E-88
DN18151_c1_g1_i2	#N/A	#N/A	9.18	ZMY15_MOUSE	2.40E-27
DN18154_c0_g2_i3	#N/A	#N/A	1.11	RLA11_ARATH	1.80E-30
DN18155_c0_g1_i7	#N/A	-1	#N/A	C98A2_SOYBN	7.00E-152
DN18161_c0_g1_i11	#N/A	#N/A	0.89	ADK1_ARATH	2.40E-73
DN18163_c1_g1_i16	#N/A	8.35	#N/A	WDR44_XENLA	2.30E-54
DN18168_c0_g8_i1	#N/A	2.27	#N/A	EF2_METTH	1.8
DN18169_c1_g1_i5	#N/A	1.34	#N/A	DADA_SPHWW	0.42
DN18176_c0_g2_i2	#N/A	-8.9	#N/A	OML4_ORYSJ	8.00E-134
DN18176_c0_g2_i3	#N/A	9.21	#N/A	OML4_ORYSJ	5.00E-134
DN18180_c2_g2_i3	#N/A	-1.07	#N/A	MYBS3_ORYSJ	2.10E-42
DN18186_c1_g1_i12	#N/A	#N/A	0.67	BRTL1_ARATH	5.00E-174

Isoform ID	Log_2(#60tr/nt)	Log_2(#20tr/nt)	Log_2(#60tr/#20t	Annotation_protein	e_value_annotation
DN18186_c1_g2_i1	#N/A	1.23	#N/A	WRK70_SOLLC	7.30E-39
DN18196_c0_g1_i1	#N/A	#N/A	-6.26	TM135_XENLA	6.10E-06
DN18196_c0_g3_i1	#N/A	#N/A	-1.44	LTN1_ARATH	0
DN18199_c0_g4_i4	#N/A	#N/A	1.35	PBL21_ARATH	1.00E-175
DN18201_c0_g2_i6	#N/A	2.25	#N/A	SCL13_ARATH	0
DN18202_c0_g3_i1	#N/A	-0.62	#N/A	TIL_ARATH	7.20E-95
DN18202_c0_g4_i11	#N/A	#N/A	8.23	GAT2_ARATH	2.10E-65
DN18203_c0_g1_i13	#N/A	#N/A	-8.22	RTC1_KLULA	0.27
DN18213_c0_g1_i1	#N/A	#N/A	0.61	TRXM4_ARATH	5.40E-61
DN18213_c0_g1_i5	#N/A	#N/A	0.58	TRXM4_ARATH	7.70E-61
DN18214_c1_g1_i15	#N/A	10.15	-8.47	PSB7B_ARATH	2.00E-179
DN18214_c1_g1_i17	#N/A	1.53	#N/A	PSB7B_ARATH	2.00E-179
DN18214_c1_g1_i20	#N/A	-2	2	PSB7B_ARATH	2.00E-179
DN18221_c0_g1_i16	#N/A	-0.85	#N/A	BBD1_ARATH	2.00E-166
DN18221_c0_g1_i21	#N/A	-0.71	#N/A	BBD1_ARATH	4.00E-163
DN18230_c0_g2_i27	#N/A	#N/A	-7.86	SDS22_CAEEL	2.60E-19
DN18232_c0_g1_i12	#N/A	#N/A	-1.3	GGAP1_ARATH	2.00E-132
DN18232_c0_g1_i21	#N/A	#N/A	-1.72	GGAP1_ARATH	1.90E-81
DN18232_c0_g2_i2	#N/A	#N/A	0.9	UAF30_SCHPO	5.40E-14
DN18232_c0_g2_i9	#N/A	#N/A	0.76	UAF30_SCHPO	7.90E-14
DN18232_c0_g4_i2	#N/A	#N/A	0.71	PDF1_ARATH	2.60E-07
DN18232_c0_g5_i1	#N/A	#N/A	-0.62	.	#N/A
DN18234_c0_g3_i3	#N/A	#N/A	9.75	PER4_VITVI	1.70E-64
DN18238_c0_g1_i3	#N/A	#N/A	-8.14	UVRC_PETMO	3.1
DN18248_c0_g1_i10	#N/A	#N/A	1.14	LPAT2_BRANA	0
DN18254_c0_g1_i4	#N/A	-0.9	#N/A	AT74_ARATH	5.00E-99
DN18254_c0_g1_i5	#N/A	-0.81	#N/A	AT74_ARATH	7.00E-102
DN18254_c0_g4_i6	#N/A	2.73	-2.25	PMD2_ARATH	7.10E-45
DN18258_c0_g4_i2	#N/A	#N/A	1.76	UCC1_ARATH	6.60E-20
DN18258_c0_g4_i3	#N/A	#N/A	1.28	UCC1_ARATH	4.00E-19
DN18261_c0_g2_i18	#N/A	-1.51	#N/A	CIPK1_ARATH	0
DN18268_c0_g1_i5	#N/A	1.29	-1.31	WRK53_ARATH	6.10E-45
DN18268_c0_g1_i6	#N/A	2.51	-1.79	WRK46_ARATH	1.30E-45
DN18268_c0_g4_i1	#N/A	1.83	-1.89	WRK41_ARATH	2.10E-47
DN18271_c1_g1_i7	#N/A	-0.77	#N/A	CXE15_ARATH	7.00E-121
DN18272_c0_g1_i10	#N/A	#N/A	-8.54	FBK8_ARATH	0
DN18274_c1_g2_i2	#N/A	#N/A	1.42	KPRO_MAIZE	4.00E-152
DN18288_c0_g2_i1	#N/A	-7.41	#N/A	E1BS_ADE07	3.3
DN18296_c1_g2_i4	#N/A	#N/A	-7.38	XMT1_COFCA	2.00E-99

Isoform ID	Log_2(#60tr/nt)	Log_2(#20tr/nt)	Log_2(#60tr/#20t	Annotation_protein	e_value_annotation
DN18297_c0_g3_i1	#N/A	4.11	#N/A	PPO_VICFA	0
DN18300_c0_g2_i6	#N/A	-1.58	#N/A	LOFG2_ARATH	7.00E-128
DN18304_c0_g3_i3	#N/A	5.99	-8.02	ARR2_ARATH	1.20E-32
DN18309_c2_g4_i3	#N/A	-9.11	#N/A	RS10B_BOVIN	3
DN18309_c2_g5_i3	#N/A	-1.41	#N/A	MYB48_ARATH	2.50E-09
DN18309_c2_g5_i4	#N/A	-1.35	#N/A	MYB48_ARATH	7.30E-44
DN18309_c2_g5_i9	#N/A	-1.96	#N/A	.	#N/A
DN18319_c0_g1_i8	#N/A	#N/A	7.8	SCAI_HUMAN	1.30E-48
DN18334_c0_g1_i23	#N/A	#N/A	-5.19	YL054_MIMIV	0.84
DN18341_c1_g3_i2	#N/A	-0.58	#N/A	BAM3_ARATH	2.00E-143
DN18342_c0_g1_i13	#N/A	7.91	#N/A	.	#N/A
DN18342_c0_g1_i9	#N/A	#N/A	1.7	SCRK1_ORYSI	4.10E-15
DN18352_c0_g2_i13	#N/A	#N/A	-7.88	NNRD_VITVI	0
DN18355_c0_g1_i4	#N/A	-0.78	#N/A	KUA1_ARATH	4.10E-59
DN18359_c1_g1_i5	#N/A	#N/A	-8.04	.	#N/A
DN18376_c0_g1_i2	#N/A	-1.33	1.28	RUBA_PEA	0
DN18376_c0_g1_i6	#N/A	#N/A	0.74	RUBA_PEA	0
DN18378_c0_g1_i1	#N/A	#N/A	-8.84	NIN3_ORYSJ	0
DN18386_c0_g1_i4	#N/A	#N/A	-1.62	SMC3_ARATH	0
DN18388_c1_g2_i3	#N/A	#N/A	0.73	UVB31_ARATH	3.70E-26
DN18409_c0_g1_i2	#N/A	9.33	#N/A	GRWD1_DICDI	1.00E-89
DN18409_c0_g1_i4	#N/A	-8.9	#N/A	GRWD1_DICDI	9.80E-20
DN18409_c0_g1_i6	#N/A	-8.01	#N/A	GRWD1_DICDI	1.20E-89
DN18409_c0_g4_i4	#N/A	#N/A	-1.31	AB4C_ARATH	0
DN18412_c0_g1_i9	#N/A	#N/A	0.84	ODP23_ARATH	0
DN18414_c0_g2_i2	#N/A	-2.59	#N/A	.	#N/A
DN18422_c1_g1_i9	#N/A	#N/A	1.02	.	#N/A
DN18425_c2_g1_i1	#N/A	#N/A	0.84	RS13_SOYBN	5.00E-108
DN18425_c2_g1_i5	#N/A	#N/A	0.94	RS13_SOYBN	3.10E-46
DN18425_c2_g1_i8	#N/A	#N/A	0.81	RS13_SOYBN	5.00E-108
DN18426_c0_g6_i4	#N/A	-2.58	#N/A	WTR38_ARATH	1.50E-29
DN18430_c1_g2_i5	#N/A	#N/A	0.76	FTRC_SOYBN	5.00E-106
DN18432_c0_g2_i3	#N/A	#N/A	0.79	PTST2_ARATH	4.00E-117
DN18435_c0_g1_i3	#N/A	#N/A	-7.07	BRCC3_XENTR	7.60E-53
DN18440_c0_g2_i16	#N/A	#N/A	10.05	RN208_MOUSE	2.20E-05
DN18443_c0_g2_i2	#N/A	#N/A	0.67	MORF2_ARATH	6.00E-116
DN18443_c0_g2_i9	#N/A	#N/A	0.65	MORF2_ARATH	2.00E-114
DN18443_c0_g3_i1	#N/A	#N/A	0.65	MORF2_ARATH	4.00E-110
DN18453_c0_g2_i5	#N/A	#N/A	1.23	CSP4_ARATH	8.80E-34

Isoform ID	Log_2(#60tr/nt)	Log_2(#20tr/nt)	Log_2(#60tr/#20t	Annotation_protein	e_value_annotation
DN18454_c0_g1_i18	#N/A	#N/A	0.89	.	#N/A
DN18454_c0_g1_i26	#N/A	#N/A	0.79	.	#N/A
DN18459_c0_g1_i17	#N/A	#N/A	-0.84	CHKA_MOUSE	1.9
DN18459_c0_g1_i8	#N/A	-1.05	#N/A	CHKA_MOUSE	1.9
DN18460_c0_g1_i12	#N/A	#N/A	-2.33	SGT1A_ARATH	4.00E-47
DN18462_c0_g2_i4	#N/A	7.73	#N/A	BH093_ARATH	2.10E-87
DN18478_c0_g2_i15	#N/A	#N/A	-4.38	RFI2_ARATH	6.00E-68
DN18482_c0_g1_i15	#N/A	#N/A	6.68	VP13B_HUMAN	9.6
DN18484_c0_g1_i4	#N/A	#N/A	0.54	MORF8_ARATH	1.30E-97
DN18496_c0_g1_i23	#N/A	#N/A	-1.18	DEXHE_ARATH	0
DN18502_c0_g1_i3	#N/A	#N/A	-8.27	AT18C_ARATH	0
DN18507_c0_g1_i1	#N/A	#N/A	5.84	PSY_CUCME	0
DN18507_c0_g1_i15	#N/A	#N/A	-5.08	PSY_CUCME	0
DN18512_c1_g2_i2	#N/A	#N/A	0.79	PSBP_PEA	1.00E-132
DN18514_c0_g3_i5	#N/A	5.17	#N/A	NAC90_ARATH	1.50E-75
DN18525_c0_g3_i1	#N/A	2.13	-1.42	.	#N/A
DN18531_c0_g1_i1	#N/A	-1.1	#N/A	AEL1_ARATH	4.80E-88
DN18532_c0_g2_i2	#N/A	#N/A	-0.99	MPK10_ORYSJ	2.30E-87
DN18533_c0_g7_i3	#N/A	#N/A	0.81	NDRP1_ARATH	4.10E-20
DN18535_c0_g1_i6	#N/A	#N/A	0.91	.	#N/A
DN18536_c1_g2_i10	#N/A	#N/A	1.29	ATB16_ARATH	1.20E-79
DN18536_c1_g2_i19	#N/A	#N/A	0.78	ATHB6_ARATH	6.50E-62
DN18539_c0_g1_i7	#N/A	#N/A	8.2	Y5162_ARATH	2.50E-55
DN18544_c2_g2_i5	#N/A	-0.93	#N/A	Y2729_PELTS	0.49
DN18550_c0_g1_i10	#N/A	#N/A	0.75	1433A_SOYBN	0
DN18550_c0_g1_i9	#N/A	5.28	#N/A	1433A_SOYBN	8.00E-175
DN18556_c0_g2_i1	#N/A	#N/A	0.75	PB27A_ARATH	2.00E-62
DN18563_c0_g2_i5	#N/A	#N/A	3.87	YL055_MIMIV	1.2
DN18570_c2_g3_i2	#N/A	#N/A	1.04	.	#N/A
DN18570_c2_g3_i3	#N/A	#N/A	1.21	.	#N/A
DN18570_c2_g3_i6	#N/A	#N/A	1.79	.	#N/A
DN18570_c2_g4_i2	#N/A	#N/A	1.3	.	#N/A
DN18571_c0_g1_i3	#N/A	-0.83	#N/A	H1_SOLPN	9.30E-58
DN18571_c0_g1_i5	#N/A	-1.01	#N/A	H1_SOLPN	5.20E-65
DN18578_c0_g2_i1	#N/A	-1	#N/A	FTSA_RHIRD	1.8
DN18578_c0_g7_i1	#N/A	-1.16	1.24	.	#N/A
DN18588_c0_g1_i13	#N/A	#N/A	8.99	PP169_ARATH	5.00E-148
DN18599_c0_g5_i4	#N/A	#N/A	0.78	THI41_VITVI	0
DN18608_c0_g3_i4	#N/A	8.73	#N/A	PECT1_ARATH	0

Isoform ID	Log_2(#60tr/nt)	Log_2(#20tr/nt)	Log_2(#60tr/#20t	Annotation_protein	e_value_annotation
DN18616_c0_g1_i8	#N/A	#N/A	-8.61	YE7A_SCHPO	4.30E-14
DN18639_c0_g1_i3	#N/A	-1.29	#N/A	VITH4_ARATH	3.00E-100
DN18642_c0_g1_i4	#N/A	#N/A	-8.29	PTHC_ARATH	1.10E-39
DN18645_c2_g1_i16	#N/A	#N/A	1.24	RL192_ARATH	1.00E-126
DN18645_c2_g1_i2	#N/A	#N/A	0.61	RL192_ARATH	2.00E-122
DN18652_c1_g1_i4	#N/A	#N/A	-0.77	SDS22_CAEEL	4.20E-12
DN18652_c1_g2_i2	#N/A	#N/A	-0.79	GCS23_RHOJR	0.94
DN18652_c1_g2_i5	#N/A	#N/A	-1.09	LRCC1_MOUSE	2.20E-05
DN18664_c0_g2_i1	#N/A	2.65	#N/A	PCS1L_ARATH	2.50E-73
DN18665_c0_g1_i4	#N/A	7.68	-7.81	DTX46_ARATH	4.00E-145
DN18692_c0_g4_i1	#N/A	#N/A	-1.13	GPT2_ARATH	1.60E-40
DN18704_c0_g2_i3	#N/A	7.58	#N/A	.	#N/A
DN18707_c0_g2_i3	#N/A	#N/A	1.42	ADT3_ARATH	3.00E-130
DN18717_c0_g1_i8	#N/A	#N/A	-1.71	LRK72_ARATH	0
DN18720_c1_g2_i3	#N/A	#N/A	0.64	CYSKP_SOLTU	4.60E-93
DN18721_c1_g3_i4	#N/A	-7.4	#N/A	.	#N/A
DN18722_c1_g1_i10	#N/A	#N/A	-8.87	SD11_ARATH	2.10E-86
DN18722_c1_g1_i4	#N/A	#N/A	-8.52	Y4729_ARATH	6.00E-132
DN18732_c0_g5_i2	#N/A	#N/A	0.59	YF14_SCHPO	0.32
DN18732_c0_g5_i3	#N/A	#N/A	0.83	YF14_SCHPO	0.36
DN18737_c0_g2_i1	#N/A	1.37	#N/A	.	#N/A
DN18737_c0_g3_i2	#N/A	#N/A	10.33	.	#N/A
DN18737_c0_g3_i5	#N/A	1.49	#N/A	.	#N/A
DN18743_c0_g5_i1	#N/A	#N/A	-2.19	HS22M_SOYBN	7.00E-65
DN18750_c0_g1_i7	#N/A	#N/A	-1.37	NCED1_PHAVU	0
DN18752_c0_g2_i4	#N/A	-9.56	#N/A	LYSM5_ARTBC	1.3
DN18764_c0_g3_i5	#N/A	#N/A	-1.04	AN32_ARATH	1.00E-107
DN18765_c0_g2_i1	#N/A	-1.04	1.02	.	#N/A
DN18774_c0_g1_i3	#N/A	#N/A	11.36	PPH_ARATH	9.30E-58
DN18792_c1_g1_i19	#N/A	#N/A	0.81	Y5486_ARATH	9.30E-74
DN18794_c1_g1_i1	#N/A	-0.58	#N/A	PXL2C_ARATH	2.70E-25
DN18800_c1_g2_i2	#N/A	3	-2.35	HSP83_IPONI	0
DN18806_c0_g1_i18	#N/A	#N/A	0.83	RL7A2_ORYSJ	3.00E-101
DN18806_c0_g1_i2	#N/A	#N/A	0.77	.	#N/A
DN18806_c0_g1_i39	#N/A	#N/A	0.85	RL7A2_ORYSJ	7.00E-171
DN18807_c0_g1_i10	#N/A	-0.85	#N/A	Y3081_ARATH	4.20E-06
DN18824_c0_g1_i8	#N/A	#N/A	2.47	.	#N/A
DN18830_c1_g3_i2	#N/A	#N/A	0.78	RL372_ORYSJ	2.10E-60
DN18831_c0_g1_i8	#N/A	-0.99	#N/A	SYM1_KLULA	7.80E-17

Isoform ID	Log_2(#60tr/nt)	Log_2(#20tr/nt)	Log_2(#60tr/#20t	Annotation_protein	e_value_annotation
DN18834_c0_g4_i1	#N/A	#N/A	-1.78	RLT2_ARATH	1.00E-168
DN18838_c0_g1_i13	#N/A	#N/A	-7.56	.	#N/A
DN18838_c0_g1_i4	#N/A	-7.51	#N/A	HAT22_ARATH	4.20E-13
DN18852_c0_g1_i12	#N/A	#N/A	0.63	EGY2_ARATH	0
DN18873_c1_g1_i6	#N/A	#N/A	0.88	ACCD_PSEE4	2.4
DN18874_c0_g2_i4	#N/A	#N/A	0.69	EBP1_SOLTU	3.60E-63
DN18883_c0_g3_i3	#N/A	#N/A	-0.69	RPM1_ARATH	5.00E-116
DN18890_c0_g1_i2	#N/A	#N/A	0.93	RS261_ARATH	3.80E-67
DN18890_c0_g3_i1	#N/A	#N/A	0.73	.	#N/A
DN18891_c0_g3_i2	#N/A	#N/A	0.73	ACPM2_ARATH	1.50E-58
DN18891_c0_g3_i3	#N/A	#N/A	0.65	ACPM2_ARATH	2.20E-59
DN18896_c0_g1_i6	#N/A	2.2	#N/A	SKU5_ARATH	0
DN18897_c0_g1_i10	#N/A	#N/A	0.91	RS14_LUPLU	7.40E-93
DN18897_c0_g1_i13	#N/A	#N/A	1.01	.	#N/A
DN18897_c0_g1_i3	#N/A	#N/A	0.83	RS14_LUPLU	7.40E-93
DN18897_c0_g1_i4	#N/A	#N/A	1.04	RS14_LUPLU	2.60E-88
DN18897_c0_g1_i7	#N/A	#N/A	1.14	RS14_LUPLU	7.40E-93
DN18904_c0_g1_i14	#N/A	#N/A	12.72	MSBP1_ARATH	4.80E-95
DN18904_c0_g1_i9	#N/A	#N/A	-11.57	MSBP1_ORYSJ	2.30E-43
DN18907_c0_g1_i1	#N/A	#N/A	9.87	SSG1_IPOBA	0
DN18912_c0_g1_i7	#N/A	9.99	#N/A	GUN6_ARATH	0
DN18920_c0_g1_i1	#N/A	-0.96	#N/A	HIS7_PEA	1.00E-152
DN18922_c0_g1_i1	#N/A	#N/A	1.05	R10A_ORYSJ	4.00E-130
DN18922_c0_g1_i15	#N/A	#N/A	0.88	R10A_ORYSJ	5.90E-87
DN18922_c0_g1_i27	#N/A	#N/A	1.01	R10A_ORYSJ	3.00E-128
DN18922_c0_g1_i4	#N/A	#N/A	-8.54	.	#N/A
DN18922_c0_g1_i6	#N/A	#N/A	0.96	R10A_ORYSJ	6.00E-129
DN18929_c0_g1_i7	#N/A	#N/A	0.8	RL321_ARATH	3.80E-83
DN18930_c0_g1_i1	#N/A	3.05	#N/A	ABAH2_SOLLC	0
DN18930_c0_g1_i3	#N/A	3.42	#N/A	ABAH2_SOLLC	0
DN18930_c0_g1_i4	#N/A	8.03	#N/A	ABAH2_SOLLC	0
DN18936_c0_g1_i13	#N/A	#N/A	-1.86	SLX4_VANPO	4.2
DN18944_c0_g1_i17	#N/A	#N/A	-1.59	PYRG_METTP	0.66
DN18948_c0_g2_i2	#N/A	#N/A	7.84	.	#N/A
DN18954_c0_g1_i3	#N/A	-1.18	1.09	Y471_RICPR	2.80E-08
DN18961_c0_g1_i2	#N/A	#N/A	0.84	BH137_ARATH	3.60E-59
DN18961_c0_g2_i3	#N/A	#N/A	-7.96	CDKF4_ORYSJ	2.60E-79
DN18970_c0_g1_i3	#N/A	3.06	#N/A	ASOL_TOBAC	0
DN18970_c0_g2_i1	#N/A	8.39	#N/A	NIA2_SOYBN	0

Isoform ID	Log_2(#60tr/nt)	Log_2(#20tr/nt)	Log_2(#60tr/#20t	Annotation_protein	e_value_annotation
DN18979_c0_g1_i7	#N/A	#N/A	-1.2	RDRP_CMVIX	3
DN18983_c2_g1_i2	#N/A	#N/A	8.46	IQD1_ARATH	6.70E-93
DN18983_c2_g1_i3	#N/A	2.04	#N/A	IQD1_ARATH	3.50E-92
DN18983_c2_g1_i6	#N/A	-8.62	#N/A	IQD1_ARATH	6.50E-92
DN18983_c2_g1_i8	#N/A	-9.12	#N/A	IQD1_ARATH	6.50E-92
DN18992_c0_g1_i3	#N/A	9.57	-9.7	YJIA_SCHPO	0.0005
DN19016_c0_g1_i12	#N/A	#N/A	0.89	.	#N/A
DN19016_c0_g1_i15	#N/A	#N/A	0.87	.	#N/A
DN19025_c0_g1_i12	#N/A	-2.17	#N/A	SYP51_ARATH	7.00E-122
DN19027_c0_g3_i8	#N/A	#N/A	1.11	AMT11_SOLLC	1.00E-117
DN19032_c1_g2_i5	#N/A	#N/A	0.95	SRP68_MOUSE	4.40E-95
DN19037_c0_g3_i3	#N/A	0.79	-0.97	SCL5_ARATH	0
DN19047_c0_g1_i10	#N/A	#N/A	0.51	EF1A_SOYBN	0
DN19047_c0_g2_i2	#N/A	#N/A	0.63	EF1A_MANES	0
DN19052_c2_g2_i2	#N/A	#N/A	-5.04	RANG_HUMAN	2.2
DN19072_c0_g2_i9	#N/A	#N/A	-2.11	MYT1_MOUSE	3
DN19087_c0_g1_i13	#N/A	#N/A	-8.96	APO3_ARATH	2.90E-98
DN19087_c0_g1_i27	#N/A	#N/A	8.94	APO3_ARATH	2.90E-98
DN19092_c0_g3_i2	#N/A	-0.9	#N/A	U74F1_ARATH	6.00E-165
DN19104_c0_g1_i10	#N/A	#N/A	1.24	B3GTB_ARATH	1.00E-177
DN19104_c0_g3_i6	#N/A	#N/A	0.69	TSN_CHICK	1.00E-44
DN19107_c0_g1_i3	#N/A	#N/A	1.28	RH31_ARATH	0
DN19109_c0_g1_i10	#N/A	#N/A	0.66	.	#N/A
DN19109_c0_g1_i6	#N/A	#N/A	0.74	PSAK_MEDSA	5.40E-52
DN19109_c0_g1_i9	#N/A	#N/A	0.67	PSAK_MEDSA	1.20E-54
DN19109_c0_g5_i8	#N/A	-1.08	#N/A	MABP1_HUMAN	5
DN19114_c0_g1_i1	#N/A	#N/A	9.62	VL2_HPV53	3.8
DN19114_c0_g1_i4	#N/A	#N/A	7.89	XXT2_ARATH	0
DN19114_c0_g1_i8	#N/A	1.37	#N/A	VL2_HPV53	3.8
DN19119_c0_g1_i17	#N/A	0.91	#N/A	NRPA_SOYBN	0
DN19119_c0_g1_i21	#N/A	0.62	#N/A	NRPA_SOYBN	0
DN19119_c0_g1_i6	#N/A	#N/A	-0.77	NRPB_SOYBN	0
DN19126_c0_g3_i3	#N/A	#N/A	0.75	.	#N/A
DN19130_c0_g2_i1	#N/A	#N/A	1.04	IF5_PHAVU	0
DN19137_c0_g3_i7	#N/A	7.47	#N/A	.	#N/A
DN19138_c0_g6_i1	#N/A	-0.88	#N/A	GSTUI_ARATH	3.50E-83
DN19140_c0_g3_i2	#N/A	#N/A	-7.98	NFYA3_ARATH	6.60E-39
DN19152_c0_g2_i6	#N/A	#N/A	8.74	SRS5_ARATH	0.13
DN19153_c0_g1_i6	#N/A	-0.83	#N/A	HMGL_SOYBN	1.00E-103

Isoform ID	Log_2(#60tr/nt)	Log_2(#20tr/nt)	Log_2(#60tr/#20t	Annotation_protein	e_value_annotation
DN19159_c1_g3_i6	#N/A	1.18	#N/A	HSP82_ORYSJ	0
DN19167_c0_g1_i6	#N/A	#N/A	0.76	PSBW_SPIOL	4.90E-50
DN19167_c0_g1_i7	#N/A	-1.23	#N/A	PSBW_SPIOL	3.90E-52
DN19167_c0_g1_i8	#N/A	#N/A	0.81	PSBW_SPIOL	7.30E-40
DN19168_c0_g1_i10	#N/A	#N/A	0.8	TPIC_ARATH	5.00E-120
DN19171_c1_g4_i1	#N/A	#N/A	-0.86	AB12C_ARATH	0
DN19172_c3_g2_i3	#N/A	-1.53	#N/A	.	#N/A
DN19175_c0_g1_i7	#N/A	8.21	#N/A	ILL6_ARATH	3.00E-180
DN19182_c0_g1_i3	#N/A	#N/A	0.65	IF5A2_NICPL	2.00E-110
DN19182_c0_g1_i7	#N/A	#N/A	0.71	IF5A2_NICPL	2.00E-110
DN19193_c0_g4_i5	#N/A	#N/A	-9.15	ATM_ARATH	2.20E-07
DN19219_c0_g1_i4	#N/A	#N/A	-2.88	M3K1_ARATH	3.00E-145
DN19224_c0_g1_i3	#N/A	#N/A	1.2	AIR3_ARATH	0
DN19246_c0_g5_i1	#N/A	#N/A	1.03	RL35_EUPES	6.50E-76
DN19246_c0_g5_i3	#N/A	#N/A	1.14	RL35_EUPES	6.50E-76
DN19246_c0_g5_i4	#N/A	#N/A	1.08	RL35_EUPES	6.50E-76
DN19247_c0_g1_i1	#N/A	-7.69	#N/A	GPMA1_ARATH	4.00E-123
DN19249_c0_g1_i14	#N/A	-0.96	1.3	GIL1_ARATH	1.90E-67
DN19249_c0_g1_i3	#N/A	#N/A	-1.2	GIL1_ARATH	1.90E-67
DN19254_c0_g2_i3	#N/A	#N/A	1.01	LOX21_SOLTU	0
DN19260_c0_g1_i4	#N/A	#N/A	0.86	RL11_MEDSA	3.20E-75
DN19260_c0_g1_i6	#N/A	#N/A	0.81	RL11_MEDSA	7.00E-132
DN19260_c0_g1_i8	#N/A	#N/A	0.87	RL11_MEDSA	5.30E-92
DN19263_c0_g1_i12	#N/A	#N/A	1.01	GME1_ORYSJ	0
DN19269_c0_g4_i5	#N/A	#N/A	0.55	HEBP2_HUMAN	3.50E-25
DN19271_c0_g2_i1	#N/A	2.42	#N/A	HIP39_ARATH	1.50E-19
DN19271_c0_g6_i1	#N/A	1.46	#N/A	.	#N/A
DN19283_c0_g2_i2	#N/A	#N/A	-8.5	PHI1_TOBAC	2.00E-166
DN19287_c0_g1_i6	#N/A	8.01	#N/A	TBA4_GOSHI	3.00E-113
DN19297_c1_g1_i10	#N/A	#N/A	0.83	RL74_ARATH	1.00E-154
DN19297_c1_g1_i6	#N/A	#N/A	0.74	RL74_ARATH	2.00E-154
DN19300_c0_g1_i7	#N/A	#N/A	0.8	RL222_ARATH	3.30E-65
DN19309_c0_g2_i12	#N/A	1.77	#N/A	.	#N/A
DN19325_c1_g1_i6	#N/A	#N/A	-8.75	ZNTB_PECAS	1.80E-13
DN19325_c1_g1_i7	#N/A	#N/A	-7.34	ZNTB_PECAS	1.80E-13
DN19335_c0_g2_i9	#N/A	#N/A	-0.64	Y4230_ARATH	9.40E-92
DN19337_c0_g2_i5	#N/A	#N/A	-1.58	DTX35_ARATH	4.00E-138
DN19338_c0_g2_i3	#N/A	#N/A	-9.09	AK10B_ARATH	3.20E-48
DN19339_c0_g2_i15	#N/A	5.37	#N/A	ASHH3_ARATH	3.00E-149

Isoform ID	Log_2(#60tr/nt)	Log_2(#20tr/nt)	Log_2(#60tr/#20t	Annotation_protein	e_value_annotation
DN19357_c0_g1_i3	#N/A	#N/A	-7.46	PUB4_ARATH	2.90E-12
DN19361_c0_g1_i4	#N/A	1.36	-1.11	C3H29_ARATH	7.00E-51
DN19361_c0_g1_i7	#N/A	1.42	-1.07	C3H29_ARATH	4.00E-44
DN19362_c0_g1_i3	#N/A	-9.86	#N/A	BECN1_ARATH	0
DN19378_c0_g2_i14	#N/A	#N/A	-4.8	BC10_ORYSJ	5.30E-36
DN19378_c0_g2_i31	#N/A	5	#N/A	YBDL_ECOLI	2.70E-26
DN19379_c0_g1_i2	#N/A	#N/A	0.75	INSM2_HUMAN	0.048
DN19379_c0_g1_i5	#N/A	#N/A	0.79	RR31_ARATH	1.80E-28
DN19395_c1_g1_i6	#N/A	1.03	#N/A	WAK2_ARATH	3.00E-176
DN19397_c0_g2_i17	#N/A	#N/A	-0.83	MTBC_POPTR	0
DN19404_c0_g2_i16	#N/A	#N/A	-8.5	IPPK_ARATH	3.00E-84
DN19414_c0_g1_i6	#N/A	#N/A	-8.92	CFTSY_ARATH	4.00E-104
DN19415_c0_g1_i5	#N/A	#N/A	0.64	TAF1B_ORYSJ	1.5
DN19415_c0_g2_i2	#N/A	#N/A	1.05	.	#N/A
DN19418_c0_g1_i9	#N/A	#N/A	1.18	BZP17_ARATH	2.20E-38
DN19420_c0_g2_i7	#N/A	#N/A	-0.94	MARF1_CHICK	2.70E-11
DN19422_c0_g1_i1	#N/A	9.24	#N/A	EVN_ARATH	0
DN19425_c0_g1_i8	#N/A	8.18	#N/A	LFR_ARATH	0
DN19426_c1_g2_i10	#N/A	#N/A	-5.61	SKI3_ARATH	0
DN19432_c0_g2_i1	#N/A	#N/A	-4.33	ARP_ARATH	4.00E-139
DN19432_c0_g2_i11	#N/A	#N/A	7.77	ARP_ARATH	4.00E-139
DN19435_c0_g1_i1	#N/A	#N/A	0.8	RL132_BRANA	3.00E-129
DN19435_c0_g1_i3	#N/A	#N/A	0.79	RL132_BRANA	2.00E-128
DN19443_c0_g2_i14	#N/A	#N/A	-1.2	TIF6B_ARATH	1.90E-40
DN19443_c0_g2_i5	#N/A	#N/A	0.95	TIF6B_ARATH	5.80E-25
DN19448_c0_g1_i7	#N/A	4.12	#N/A	PP451_ARATH	3.00E-144
DN19463_c0_g1_i1	#N/A	#N/A	-1.63	NIK2_ARATH	1.40E-69
DN19471_c0_g1_i8	#N/A	#N/A	-0.71	AB5F_ARATH	0
DN19477_c1_g1_i5	#N/A	5.29	#N/A	SIR4_ARATH	2.00E-117
DN19484_c1_g2_i1	#N/A	#N/A	0.9	RL18A_CASSA	3.00E-120
DN19484_c1_g2_i5	#N/A	#N/A	0.75	RL18A_CASSA	1.00E-120
DN19486_c0_g1_i1	#N/A	#N/A	3.81	PXC3_ARATH	0
DN19489_c0_g2_i10	#N/A	#N/A	3.17	MANA2_ARATH	0
DN19490_c0_g1_i18	#N/A	#N/A	0.87	CATB2_ARATH	0
DN19495_c0_g2_i1	#N/A	0.94	#N/A	IQD31_ARATH	9.20E-48
DN19497_c1_g3_i1	#N/A	10	#N/A	RK176_ORYSJ	0
DN19497_c1_g3_i3	#N/A	-9.2	9.24	RK176_ORYSJ	3.00E-101
DN19498_c0_g1_i3	#N/A	#N/A	-0.77	SCP40_ARATH	0
DN19501_c0_g1_i12	#N/A	-0.68	#N/A	STT7_ARATH	0

Isoform ID	Log_2(#60tr/nt)	Log_2(#20tr/nt)	Log_2(#60tr/#20t	Annotation_protein	e_value_annotation
DN19501_c0_g1_i8	#N/A	-0.69	#N/A	STT7_ARATH	0
DN19506_c0_g5_i1	#N/A	#N/A	-1.03	SMG1_MOUSE	2.90E-14
DN19508_c1_g1_i2	#N/A	#N/A	-0.98	VIP6_ARATH	0
DN19519_c0_g1_i10	#N/A	#N/A	-5.22	RH32_ARATH	0
DN19520_c0_g1_i2	#N/A	#N/A	7.62	PMS1_ARATH	2.00E-113
DN19524_c0_g1_i6	#N/A	#N/A	-8.57	PRUN1_BOVIN	3.60E-21
DN19526_c0_g2_i4	#N/A	1.86	#N/A	LNG2_ARATH	2.30E-82
DN19531_c0_g2_i14	#N/A	#N/A	-8.05	Y2028_DICDI	3.60E-18
DN19540_c0_g5_i5	#N/A	0.98	#N/A	P2C71_ORYSJ	1.40E-48
DN19546_c1_g2_i5	#N/A	#N/A	0.65	Y4320_ARATH	3.10E-36
DN19553_c0_g3_i3	#N/A	#N/A	1.27	SEBP2_ARATH	0
DN19555_c0_g1_i1	#N/A	#N/A	0.75	.	#N/A
DN19555_c0_g2_i24	#N/A	-8	8.46	GCST_FLAAN	2.00E-124
DN19555_c0_g2_i3	#N/A	5.42	#N/A	GCST_FLAAN	4.00E-125
DN19557_c0_g2_i8	#N/A	-9.13	#N/A	GLYM1_FLAPR	4.00E-133
DN19558_c0_g2_i3	#N/A	-5.1	#N/A	UVB31_ARATH	4.00E-125
DN19558_c0_g2_i4	#N/A	7.8	-7.93	UVB31_ARATH	1.10E-22
DN19564_c0_g1_i3	#N/A	#N/A	-7.66	VRN1_ARATH	7.90E-84
DN19578_c0_g2_i5	#N/A	-12.14	11.46	SKI3_ARATH	0.005
DN19596_c0_g1_i7	#N/A	-3.63	3.99	NTF2_ARATH	2.00E-128
DN19601_c0_g1_i16	#N/A	#N/A	0.79	CB4A_SOLLC	7.00E-125
DN19601_c0_g1_i3	#N/A	#N/A	0.87	CB4A_SOLLC	6.00E-113
DN19602_c1_g2_i2	#N/A	#N/A	-0.87	SHT_ARATH	3.50E-42
DN19619_c0_g2_i11	#N/A	#N/A	-8.21	ACOT8_MOUSE	2.40E-68
DN19626_c0_g2_i11	#N/A	-9.37	#N/A	RFP4B_DANRE	1.4
DN19627_c0_g1_i9	#N/A	#N/A	9.29	RLT1_ARATH	5.10E-19
DN19632_c0_g3_i1	#N/A	#N/A	-0.82	GLGB1_PEA	1.00E-180
DN19632_c0_g3_i4	#N/A	#N/A	-0.92	GLGB1_PEA	4.00E-178
DN19647_c0_g1_i4	#N/A	-8.27	#N/A	TF3B_MOUSE	1.20E-53
DN19655_c0_g1_i2	#N/A	#N/A	-1.37	FRI2_SOYBN	0
DN19658_c0_g2_i1	#N/A	#N/A	0.75	RR6_ARATH	6.10E-92
DN19658_c0_g2_i6	#N/A	#N/A	0.8	RR6_ARATH	5.60E-90
DN19659_c1_g1_i5	#N/A	#N/A	-11.03	CYP95_ARATH	5.60E-15
DN19659_c1_g4_i5	#N/A	#N/A	0.83	TPC2L_DICDI	6.10E-45
DN19660_c0_g1_i20	#N/A	#N/A	2.15	.	#N/A
DN19661_c0_g1_i10	#N/A	#N/A	-8.11	PP4R3_DANRE	7.00E-105
DN19663_c0_g2_i4	#N/A	#N/A	2.42	.	#N/A
DN19663_c0_g2_i8	#N/A	-0.7	1.6	.	#N/A
DN19686_c0_g3_i1	#N/A	-1.02	#N/A	MES17_ARATH	2.00E-120

Isoform ID	Log_2(#60tr/nt)	Log_2(#20tr/nt)	Log_2(#60tr/#20t	Annotation_protein	e_value_annotation
DN19686_c0_g4_i5	#N/A	#N/A	0.83	RL4_ALISL	0.079
DN19687_c0_g3_i20	#N/A	#N/A	-7.45	.	#N/A
DN19690_c0_g3_i2	#N/A	#N/A	-9.17	TCPE_ARATH	0
DN19699_c0_g1_i6	#N/A	#N/A	0.88	IFI44_MOUSE	0.5
DN19707_c0_g2_i1	#N/A	#N/A	-5.97	.	#N/A
DN19710_c0_g1_i1	#N/A	#N/A	1.37	YEQ6_YEAST	0.68
DN19710_c0_g1_i7	#N/A	#N/A	1.76	.	#N/A
DN19711_c0_g1_i9	#N/A	#N/A	-7.66	PSI3_ARATH	0.00027
DN19714_c0_g1_i1	#N/A	#N/A	-1.58	Y5838_ARATH	6.00E-167
DN19716_c0_g1_i21	#N/A	8.76	#N/A	NRAM1_ARATH	6.00E-143
DN19736_c0_g1_i13	#N/A	#N/A	0.92	QPCT_ARATH	2.00E-105
DN19750_c0_g2_i4	#N/A	#N/A	-1.35	Y5262_ARATH	0
DN19751_c1_g1_i2	#N/A	6.18	#N/A	DOF36_ARATH	5.00E-53
DN19751_c1_g1_i7	#N/A	-8.11	#N/A	DOF36_ARATH	8.80E-56
DN19751_c1_g2_i2	#N/A	2.45	#N/A	.	#N/A
DN19753_c1_g1_i1	#N/A	9.01	#N/A	NSL1_ARATH	0
DN19770_c1_g1_i10	#N/A	#N/A	-1.02	GRDP1_ARATH	0
DN19778_c1_g2_i15	#N/A	#N/A	0.82	NEET_ARATH	1.40E-39
DN19780_c0_g1_i12	#N/A	-8.23	#N/A	DPOD_CANAX	8.8
DN19785_c1_g1_i7	#N/A	#N/A	-5.01	FAMA_ARATH	2.00E-146
DN19788_c0_g1_i1	#N/A	#N/A	-0.84	SPA15_IPOBA	2.00E-145
DN19788_c0_g1_i7	#N/A	#N/A	-9.63	SPA15_IPOBA	4.00E-141
DN19789_c0_g7_i5	#N/A	-1.14	#N/A	.	#N/A
DN19796_c1_g7_i1	#N/A	#N/A	-1.01	.	#N/A
DN19800_c0_g1_i28	#N/A	-10.17	#N/A	PX24B_DICDI	6.50E-26
DN19812_c0_g1_i2	#N/A	#N/A	0.97	BCP_PEA	2.00E-06
DN19814_c0_g2_i9	#N/A	#N/A	-1.58	IF4B_HUMAN	0.51
DN19824_c0_g2_i4	#N/A	#N/A	-1.31	DAD2_PETHY	3.40E-78
DN19828_c0_g4_i2	#N/A	#N/A	0.82	RK3_TOBAC	2.70E-91
DN19828_c0_g4_i3	#N/A	#N/A	0.65	RK3_SPIOL	4.00E-136
DN19828_c0_g4_i5	#N/A	#N/A	0.6	RK3_SPIOL	6.00E-134
DN19831_c0_g2_i1	#N/A	4.35	#N/A	PHI1_TOBAC	3.00E-149
DN19831_c0_g3_i1	#N/A	3.61	#N/A	PHI1_TOBAC	5.00E-175
DN19831_c0_g3_i2	#N/A	7.48	#N/A	PHI1_TOBAC	5.00E-175
DN19831_c0_g4_i1	#N/A	4.04	#N/A	PHI1_TOBAC	2.00E-153
DN19832_c2_g3_i1	#N/A	#N/A	0.62	BP73_ORYSJ	6.20E-55
DN19833_c0_g2_i10	#N/A	2.27	#N/A	ERD2_ENTHI	1.60E-31
DN19835_c0_g2_i11	#N/A	#N/A	-7.91	EML3_ARATH	6.00E-155
DN19840_c0_g1_i5	#N/A	#N/A	-7.8	POD1_ARATH	6.00E-165

Isoform ID	Log_2(#60tr/nt)	Log_2(#20tr/nt)	Log_2(#60tr/#20t	Annotation_protein	e_value_annotation
DN19841_c0_g1_i9	#N/A	-8.65	#N/A	G6PD4_ARATH	2.00E-123
DN19845_c0_g1_i15	#N/A	-8.01	#N/A	TOC90_ARATH	0
DN19852_c0_g1_i1	#N/A	#N/A	1.28	OPT3_ARATH	0
DN19867_c1_g1_i5	#N/A	1.81	#N/A	FATB_GOSHI	5.00E-177
DN19870_c1_g3_i1	#N/A	-0.95	#N/A	.	#N/A
DN19872_c0_g1_i24	#N/A	#N/A	-8.91	.	#N/A
DN19872_c0_g2_i1	#N/A	-0.67	#N/A	H12_ARATH	8.80E-28
DN19877_c0_g1_i11	#N/A	#N/A	0.58	HPR1_ARATH	0
DN19877_c0_g1_i20	#N/A	#N/A	0.86	HPR1_ARATH	0
DN19877_c0_g1_i4	#N/A	#N/A	9.35	DHGY_CUCSA	0
DN19877_c0_g1_i8	#N/A	-0.77	0.66	HPR1_ARATH	1.30E-74
DN19883_c0_g1_i1	#N/A	#N/A	0.81	.	#N/A
DN19883_c0_g1_i14	#N/A	7.9	-8.03	G3PC_PETHY	1.70E-80
DN19883_c0_g1_i3	#N/A	#N/A	0.8	.	#N/A
DN19887_c0_g3_i1	#N/A	4.96	#N/A	DRE1D_ARATH	7.50E-66
DN19887_c0_g3_i2	#N/A	3.72	#N/A	DRE1D_ARATH	4.70E-68
DN19892_c0_g1_i1	#N/A	8.27	#N/A	DEAHD_ARATH	2.00E-144
DN19894_c0_g2_i10	#N/A	#N/A	0.67	.	#N/A
DN19899_c0_g3_i9	#N/A	7.95	#N/A	FRS6_ARATH	0.014
DN19904_c0_g1_i10	#N/A	-1.25	#N/A	OBL1_ARATH	5
DN19904_c0_g1_i6	#N/A	#N/A	-1.5	RPM1_ARATH	2.00E-144
DN19914_c0_g2_i5	#N/A	-2.28	2.21	PSB4_ARATH	8.00E-149
DN19923_c0_g1_i4	#N/A	7.67	-5.98	PCFS4_ARATH	6.00E-102
DN19934_c0_g1_i1	#N/A	#N/A	-0.64	.	#N/A
DN19939_c0_g6_i3	#N/A	#N/A	0.62	TMC5_RAT	0.17
DN19946_c1_g2_i3	#N/A	#N/A	8.57	HT1_ARATH	5.60E-87
DN19952_c0_g1_i11	#N/A	7.91	#N/A	LPXD_CHRSD	0.12
DN19952_c0_g2_i1	#N/A	-1.25	#N/A	.	#N/A
DN19957_c0_g1_i27	#N/A	#N/A	-1.5	TRF41_DROME	2.00E-28
DN19965_c0_g1_i3	#N/A	#N/A	9.29	ASP1_ORYSJ	3.00E-107
DN19972_c1_g1_i35	#N/A	#N/A	1.7	APA1_ARATH	0
DN19977_c0_g1_i13	#N/A	11.22	#N/A	FTSH2_ORYSJ	0
DN19981_c1_g1_i16	#N/A	#N/A	-7.88	DPH6_DANRE	7.00E-99
DN19981_c1_g1_i3	#N/A	#N/A	5.41	DPH6_SCHPO	4.90E-43
DN19991_c0_g3_i3	#N/A	#N/A	0.75	GUC2F_HUMAN	0.21
DN19997_c0_g3_i1	#N/A	1.12	#N/A	HSP83_IPONI	6.00E-177
DN20008_c1_g2_i2	#N/A	#N/A	1.74	HUTI_HUMAN	1.3
DN20009_c1_g4_i1	#N/A	#N/A	1.23	DIR11_ARATH	3.50E-45
DN20023_c0_g2_i5	#N/A	-0.93	#N/A	.	#N/A

Isoform ID	Log_2(#60tr/nt)	Log_2(#20tr/nt)	Log_2(#60tr/#20t	Annotation_protein	e_value_annotation
DN20027_c1_g4_i2	#N/A	#N/A	0.64	.	#N/A
DN20037_c0_g3_i4	#N/A	1.14	#N/A	HIP39_ARATH	7.70E-16
DN20037_c0_g3_i5	#N/A	2.88	#N/A	HIP39_ARATH	6.00E-20
DN20038_c0_g1_i2	#N/A	#N/A	-1.12	AB2C_ARATH	0
DN20038_c0_g1_i7	#N/A	#N/A	-0.71	AB2C_ARATH	0
DN20040_c0_g1_i5	#N/A	#N/A	0.93	NASP_HUMAN	5.20E-12
DN20045_c0_g1_i19	#N/A	1.94	#N/A	P2C52_ARATH	0
DN20047_c0_g1_i6	#N/A	#N/A	-1.62	TOM1_TOBAC	1.00E-126
DN20049_c0_g1_i12	#N/A	#N/A	1.51	Y084_MYCPN	3.8
DN20061_c1_g2_i9	#N/A	-1.05	#N/A	COPT6_ARATH	6.10E-30
DN20063_c1_g2_i8	#N/A	#N/A	1.31	HMDH1_SOLTU	0
DN20065_c0_g1_i5	#N/A	#N/A	-7.76	PYRD_ARATH	0
DN20069_c1_g2_i6	#N/A	#N/A	0.74	EI3GA_XENLA	7.50E-63
DN20083_c1_g1_i16	#N/A	8.23	#N/A	.	#N/A
DN20097_c2_g1_i16	#N/A	-1.62	#N/A	LPXK_SHEPW	0.71
DN20099_c0_g1_i16	#N/A	#N/A	9.7	DIM_PEA	0
DN20106_c0_g1_i3	#N/A	#N/A	-9.09	MRL7_ARATH	2.00E-118
DN20106_c0_g1_i4	#N/A	#N/A	1.2	MRL7_ARATH	2.00E-118
DN20108_c1_g1_i3	#N/A	#N/A	-8.1	BRX11_ARATH	8.00E-158
DN20109_c1_g1_i14	#N/A	#N/A	-0.85	SIGA_ARATH	0
DN20109_c1_g1_i17	#N/A	#N/A	-0.75	SIGA_ARATH	0
DN20114_c0_g1_i14	#N/A	3.81	#N/A	NEK6_ARATH	2.00E-138
DN20116_c0_g2_i5	#N/A	#N/A	6.19	C3H43_ARATH	3.80E-62
DN20133_c0_g1_i20	#N/A	2.79	#N/A	MACP2_ARATH	7.00E-88
DN20135_c0_g2_i1	#N/A	-1.09	#N/A	.	#N/A
DN20135_c0_g3_i1	#N/A	-0.85	#N/A	.	#N/A
DN20137_c1_g1_i1	#N/A	#N/A	-2.08	.	#N/A
DN20140_c1_g1_i2	#N/A	1.75	#N/A	MMSA_ARATH	0
DN20142_c0_g3_i3	#N/A	7.77	#N/A	.	#N/A
DN20147_c1_g1_i7	#N/A	7.69	#N/A	GLMM_SOLUE	0.13
DN20150_c1_g1_i19	#N/A	10.21	#N/A	VA_PHOKE	0.06
DN20150_c1_g1_i9	#N/A	-9.86	#N/A	VA_PHOKE	0.063
DN20150_c1_g2_i6	#N/A	#N/A	0.72	AB2D_ARATH	2.20E-93
DN20157_c0_g1_i10	#N/A	3.87	#N/A	EX84C_ARATH	0
DN20158_c1_g3_i8	#N/A	1.24	#N/A	VAC8_CANGA	9.60E-10
DN20163_c0_g2_i2	#N/A	4.89	#N/A	FBK22_ARATH	2.00E-123
DN20168_c0_g1_i11	#N/A	4.87	#N/A	ARAE1_ARATH	0
DN20169_c0_g1_i1	#N/A	-1.19	0.8	CP122_ARATH	5.70E-31
DN20169_c0_g1_i2	#N/A	-1.37	1.05	.	#N/A

Isoform ID	Log_2(#60tr/nt)	Log_2(#20tr/nt)	Log_2(#60tr/#20t	Annotation_protein	e_value_annotation
DN20169_c0_g1_i3	#N/A	#N/A	-12.91	CP122_ARATH	0.6
DN20169_c0_g1_i4	#N/A	-0.89	#N/A	CP122_ARATH	8.40E-33
DN20179_c0_g2_i3	#N/A	#N/A	0.61	CCB12_ORYSJ	8.20E-27
DN20183_c0_g3_i10	#N/A	8.42	#N/A	GTE12_ARATH	1.40E-20
DN20186_c0_g2_i10	#N/A	-8.41	#N/A	DUF3_ARATH	3.10E-08
DN20186_c0_g2_i12	#N/A	#N/A	-0.97	DUF3_ARATH	1.90E-08
DN20186_c0_g2_i28	#N/A	#N/A	-8.06	CMT2_ARATH	0.002
DN20207_c0_g2_i2	#N/A	#N/A	0.58	YMG12_ARATH	7.50E-65
DN20211_c1_g1_i4	#N/A	-9.13	#N/A	SYT2_ARATH	1.10E-13
DN20217_c0_g1_i3	#N/A	#N/A	2.05	ICR1_ARATH	6.60E-87
DN20228_c0_g2_i10	#N/A	1.07	#N/A	MSR1_ARATH	0
DN20228_c0_g3_i10	#N/A	#N/A	6.44	MSR1_ARATH	0
DN20234_c0_g1_i10	#N/A	7.49	#N/A	SDIR1_ARATH	6.60E-06
DN20242_c0_g1_i3	#N/A	3.02	#N/A	ACCR3_ARATH	1.30E-92
DN20245_c0_g1_i3	#N/A	#N/A	7.74	.	#N/A
DN20249_c0_g1_i18	#N/A	#N/A	-9.54	HERC2_HUMAN	6.00E-05
DN20259_c0_g1_i13	#N/A	-5.28	#N/A	MYO3_CAEEL	2.2
DN20259_c0_g1_i9	#N/A	-6.61	#N/A	MYO3_CAEEL	2.1
DN20270_c0_g1_i6	#N/A	#N/A	-2.1	MDAR_PEA	8.00E-152
DN20283_c0_g1_i15	#N/A	-0.87	#N/A	GLO1_ARATH	4.00E-166
DN20291_c1_g1_i2	#N/A	#N/A	0.92	RLA32_ARATH	6.00E-31
DN20291_c1_g1_i22	#N/A	#N/A	-3.19	CLPB1_ARATH	0
DN20291_c1_g1_i7	#N/A	#N/A	1.11	RLA3_MAIZE	2.20E-30
DN20300_c0_g1_i6	#N/A	7.72	-7.85	ANKZ1_RAT	1.50E-35
DN20301_c0_g1_i2	#N/A	-7.84	#N/A	PTAJ_PESFW	7.6
DN20308_c0_g1_i8	#N/A	7.74	#N/A	.	#N/A
DN20308_c0_g1_i9	#N/A	5.38	#N/A	.	#N/A
DN20313_c0_g2_i13	#N/A	-8.5	8.38	DGS1_ARATH	4.10E-76
DN20317_c0_g1_i20	#N/A	-1.27	#N/A	Y189_CHESB	1.9
DN20321_c0_g2_i14	#N/A	9.76	-9.89	XYLL3_ARATH	0
DN20321_c0_g2_i7	#N/A	8.62	-8.75	XYLL3_ARATH	0
DN20330_c0_g1_i1	#N/A	#N/A	0.98	RSSA_SOYBN	1.70E-80
DN20333_c1_g1_i6	#N/A	8.57	#N/A	TFB1A_ARATH	0
DN20341_c0_g1_i21	#N/A	#N/A	-1.79	YKH3_SCHPO	1.60E-06
DN20354_c1_g1_i1	#N/A	8.16	-8.29	PP346_ARATH	7.70E-90
DN20361_c1_g2_i10	#N/A	#N/A	2.92	SYL_NITOC	7.7
DN20361_c1_g2_i6	#N/A	#N/A	1.2	PPP7L_ARATH	1.90E-36
DN20361_c1_g2_i8	#N/A	#N/A	2.62	PPP7L_ARATH	1.90E-36
DN20371_c0_g1_i10	#N/A	#N/A	-7.62	AGO4_ARATH	0

Isoform ID	Log_2(#60tr/nt)	Log_2(#20tr/nt)	Log_2(#60tr/#20t	Annotation_protein	e_value_annotation
DN20385_c0_g1_i1	#N/A	#N/A	1.73	LOXA_PHAVU	0
DN20385_c0_g1_i3	#N/A	#N/A	1.77	LOXX_SOYBN	7.00E-170
DN20396_c0_g1_i14	#N/A	#N/A	-1.67	.	#N/A
DN20404_c2_g1_i3	#N/A	#N/A	0.62	PSA5_SOYBN	3.00E-177
DN20411_c0_g1_i17	#N/A	#N/A	-8.84	PGKH_TOBAC	0
DN20411_c0_g1_i18	#N/A	#N/A	1.06	PGKH_TOBAC	0
DN20411_c0_g1_i3	#N/A	#N/A	5.83	PGKH_TOBAC	0
DN20444_c0_g3_i2	#N/A	#N/A	0.64	.	#N/A
DN20444_c0_g3_i5	#N/A	#N/A	0.62	.	#N/A
DN20445_c0_g4_i1	#N/A	#N/A	-0.86	GPDL4_ARATH	0
DN20448_c0_g1_i6	#N/A	-1.75	1.94	PRS4A_ARATH	0
DN20459_c0_g4_i8	#N/A	-9.89	#N/A	BGL12_ORYSI	3.00E-115
DN20464_c1_g1_i12	#N/A	-1.82	#N/A	PIF1_ARATH	1.40E-71
DN20470_c0_g2_i2	#N/A	7.96	#N/A	NPGR1_ARATH	0
DN20477_c0_g1_i14	#N/A	#N/A	9.91	ZNHI2_BOVIN	3.20E-29
DN20477_c0_g1_i16	#N/A	#N/A	-9.65	ZNHI2_BOVIN	3.20E-29
DN20481_c0_g2_i11	#N/A	#N/A	-1.32	BAHC1_MOUSE	0.001
DN20482_c1_g5_i3	#N/A	#N/A	-3.02	.	#N/A
DN20499_c1_g2_i2	#N/A	-9.86	#N/A	CTBP_ARATH	0
DN20499_c1_g2_i5	#N/A	-9.42	#N/A	CTBP_ARATH	0
DN20499_c1_g2_i6	#N/A	1.11	#N/A	CTBP_ARATH	0
DN20502_c0_g3_i4	#N/A	-0.82	#N/A	ATPF2_SPIOL	1.80E-80
DN20513_c0_g1_i1	#N/A	#N/A	0.81	.	#N/A
DN20513_c0_g1_i2	#N/A	#N/A	0.92	.	#N/A
DN20519_c0_g2_i14	#N/A	-0.71	#N/A	GPX4_CITSI	6.00E-105
DN20519_c0_g3_i2	#N/A	#N/A	0.71	GPX4_CITSI	2.00E-102
DN20519_c0_g3_i3	#N/A	#N/A	1.02	GPX4_CITSI	2.00E-102
DN20523_c0_g1_i18	#N/A	#N/A	0.99	RS15_ORYSJ	6.20E-97
DN20523_c0_g1_i22	#N/A	#N/A	0.98	RS15_ORYSJ	1.70E-98
DN20523_c0_g1_i23	#N/A	#N/A	0.91	RS15_ORYSJ	1.00E-99
DN20523_c0_g1_i9	#N/A	#N/A	0.8	RS15_ORYSJ	3.00E-100
DN20525_c0_g3_i6	#N/A	#N/A	-1.91	MYO3_ARATH	6.00E-170
DN20526_c0_g3_i3	#N/A	#N/A	10.09	METE_PLESU	0
DN20527_c0_g1_i1	#N/A	#N/A	-0.9	CAS1_GLYGL	0
DN20534_c0_g1_i23	#N/A	#N/A	0.89	FER3_MAIZE	4.50E-62
DN20537_c0_g3_i7	#N/A	#N/A	1.05	.	#N/A
DN20540_c0_g1_i5	#N/A	#N/A	8.04	LPHN_DROMO	2.2
DN20547_c1_g3_i4	#N/A	#N/A	1.64	BPG2_ARATH	1.00E-106
DN20552_c0_g5_i1	#N/A	#N/A	0.96	RL222_ARATH	4.00E-62

Isoform ID	Log_2(#60tr/nt)	Log_2(#20tr/nt)	Log_2(#60tr/#20t	Annotation_protein	e_value_annotation
DN20553_c1_g1_i26	#N/A	-1.38	#N/A	CF107_ARATH	8.50E-71
DN20554_c0_g1_i17	#N/A	4.01	#N/A	PIGS_MOUSE	1.60E-24
DN20561_c0_g1_i15	#N/A	#N/A	-6.73	CKL2_ARATH	0
DN20562_c1_g2_i2	#N/A	#N/A	-0.98	.	#N/A
DN20565_c0_g1_i10	#N/A	5.96	-5.5	DUSA_ECOLI	7.40E-80
DN20570_c0_g1_i5	#N/A	#N/A	-7.92	NTL9_ARATH	9.80E-40
DN20573_c0_g1_i7	#N/A	-1.04	#N/A	LOFG2_ARATH	2.90E-55
DN20573_c1_g1_i13	#N/A	#N/A	6.66	GTE3_ARATH	1.20E-68
DN20574_c0_g3_i3	#N/A	-0.85	#N/A	.	#N/A
DN20575_c0_g1_i23	#N/A	#N/A	-8.43	BON1_ARATH	0
DN20578_c0_g3_i11	#N/A	2.16	#N/A	CLU_ARATH	0
DN20587_c0_g2_i1	#N/A	9.74	#N/A	APRL5_ARATH	4.00E-31
DN20587_c0_g2_i3	#N/A	-8.37	#N/A	APRL5_ARATH	3.50E-88
DN20587_c0_g2_i6	#N/A	-9.1	#N/A	APRL5_ARATH	4.00E-31
DN20589_c0_g1_i12	#N/A	7.46	#N/A	.	#N/A
DN20590_c0_g1_i14	#N/A	#N/A	-3.04	NEK4_ARATH	0
DN20594_c0_g1_i3	#N/A	#N/A	0.9	ZN593_XENTR	2.20E-17
DN20594_c0_g1_i4	#N/A	#N/A	0.85	ZN593_XENTR	2.90E-17
DN20597_c0_g1_i16	#N/A	#N/A	-7.34	RP6L3_ARATH	0
DN20599_c0_g2_i16	#N/A	8.86	#N/A	DNJC8_HUMAN	2.10E-14
DN20601_c1_g1_i8	#N/A	#N/A	0.68	IF4A3_ORYSJ	1.00E-155
DN20603_c1_g1_i1	#N/A	#N/A	0.76	.	#N/A
DN20604_c0_g2_i1	#N/A	#N/A	1.04	.	#N/A
DN20604_c0_g2_i10	#N/A	#N/A	1.17	.	#N/A
DN20604_c0_g2_i3	#N/A	#N/A	1.04	RS201_ARATH	9.50E-75
DN20604_c0_g2_i5	#N/A	#N/A	1.19	RS201_ARATH	2.90E-75
DN20604_c0_g7_i1	#N/A	#N/A	1.13	RS202_ARATH	2.70E-79
DN20606_c1_g1_i10	#N/A	#N/A	2.17	RETIC_ARATH	2.00E-177
DN20608_c0_g1_i4	#N/A	#N/A	11.62	NDUS1_SOLTU	0
DN20615_c0_g3_i4	#N/A	#N/A	-7.1	RPV1_VITRO	6.00E-34
DN20626_c0_g1_i13	#N/A	#N/A	-1.73	NET1D_ARATH	5.00E-20
DN20630_c0_g1_i9	#N/A	#N/A	-6.03	MED32_ARATH	3.50E-65
DN20631_c0_g1_i3	#N/A	#N/A	0.55	LIPA_YEAST	1.5
DN20647_c1_g1_i12	#N/A	-0.68	#N/A	MYOB7_ARATH	2.00E-164
DN20647_c1_g1_i2	#N/A	-1.59	#N/A	MYOB7_ARATH	2.00E-164
DN20648_c0_g1_i2	#N/A	#N/A	0.91	FBRL2_ARATH	2.00E-165
DN20655_c0_g2_i2	#N/A	#N/A	-1.02	UTP25_SCHJY	8.00E-117
DN20657_c0_g1_i7	#N/A	1.63	#N/A	CNGC2_ARATH	0
DN20661_c0_g1_i9	#N/A	#N/A	-8.64	SAS10_RAT	2.80E-14

Isoform ID	Log_2(#60tr/nt)	Log_2(#20tr/nt)	Log_2(#60tr/#20t	Annotation_protein	e_value_annotation
DN20670_c0_g2_i2	#N/A	#N/A	0.85	RL321_ARATH	9.30E-84
DN20670_c0_g2_i3	#N/A	#N/A	0.79	RL321_ARATH	9.30E-84
DN20684_c0_g1_i3	#N/A	#N/A	7.96	P2C46_ARATH	3.00E-145
DN20697_c1_g1_i11	#N/A	#N/A	-7.79	TRI74_HUMAN	0.55
DN20698_c0_g2_i4	#N/A	#N/A	3.95	WDR5B_ARATH	5.00E-169
DN20710_c0_g1_i2	#N/A	#N/A	7.69	Y7911_DICDI	1.5
DN20710_c0_g3_i3	#N/A	#N/A	0.83	ATP4_IPOBA	5.00E-112
DN20712_c0_g1_i12	#N/A	#N/A	8.76	PUB63_ARATH	1.70E-17
DN20715_c0_g1_i3	#N/A	0.81	#N/A	APRR1_ARATH	1.30E-75
DN20717_c0_g1_i2	#N/A	8.33	#N/A	Y2242_ARATH	0
DN20734_c0_g1_i6	#N/A	7.59	#N/A	PSBQ1_ARATH	5.70E-32
DN20734_c0_g1_i7	#N/A	#N/A	0.68	PSBQ2_ARATH	4.00E-113
DN20747_c0_g1_i6	#N/A	#N/A	0.6	ILV5_PEA	0
DN20747_c0_g2_i2	#N/A	#N/A	0.81	ILV5_PEA	0
DN20749_c1_g1_i5	#N/A	#N/A	-9.27	CCL11_ARATH	1.00E-179
DN20749_c1_g1_i6	#N/A	#N/A	-8.59	CCL11_ARATH	2.30E-07
DN20751_c0_g1_i17	#N/A	-8.1	#N/A	MBR1_ARATH	3.30E-38
DN20757_c0_g1_i3	#N/A	1.15	#N/A	CSLA2_ARATH	0
DN20757_c0_g1_i4	#N/A	1.23	#N/A	CSLA2_ARATH	0
DN20757_c0_g3_i4	#N/A	#N/A	1	.	#N/A
DN20764_c0_g2_i2	#N/A	0.83	#N/A	AGLU_SPIOL	0
DN20764_c0_g2_i3	#N/A	#N/A	10.85	AGLU_SPIOL	0
DN20772_c0_g1_i8	#N/A	1.33	-0.93	ENPL_CATRO	0
DN20774_c0_g1_i2	#N/A	2.22	#N/A	RABEK_XENLA	3.60E-33
DN20779_c0_g2_i8	#N/A	1.95	#N/A	UGDH1_SOYBN	0
DN20786_c0_g4_i3	#N/A	#N/A	1.23	LDHB_HORVU	2.80E-57
DN20790_c0_g2_i2	#N/A	1.22	#N/A	RHSC_DICD3	2.8
DN20790_c0_g2_i4	#N/A	-8.52	#N/A	RHSC_DICD3	2.8
DN20796_c0_g1_i20	#N/A	#N/A	0.78	RL5_CUCSA	4.00E-137
DN20796_c0_g1_i27	#N/A	#N/A	1.48	RL5_CUCSA	1.00E-170
DN20796_c0_g1_i5	#N/A	#N/A	0.69	RL5_CUCSA	0
DN20797_c0_g1_i4	#N/A	#N/A	-0.9	DPF3_CHICK	0.82
DN20813_c0_g2_i17	#N/A	#N/A	-1.44	DNAJ_THEM4	2.40E-16
DN20813_c0_g2_i2	#N/A	1.14	#N/A	DNJB6_MACFA	1.60E-15
DN20813_c0_g3_i4	#N/A	#N/A	0.81	.	#N/A
DN20813_c0_g3_i5	#N/A	#N/A	0.86	.	#N/A
DN20815_c1_g1_i10	#N/A	#N/A	-1.01	WRK24_ORYSJ	3.00E-142
DN20815_c1_g1_i12	#N/A	#N/A	7.9	WRK24_ORYSI	7.80E-55
DN20815_c1_g3_i1	#N/A	#N/A	1.19	.	#N/A

Isoform ID	Log_2(#60tr/nt)	Log_2(#20tr/nt)	Log_2(#60tr/#20t	Annotation_protein	e_value_annotation
DN20820_c1_g1_i3	#N/A	#N/A	-3.31	FHY3_ARATH	3.00E-120
DN20825_c0_g2_i7	#N/A	#N/A	5.36	SPD1_ARATH	4.10E-86
DN20835_c0_g1_i9	#N/A	8.47	-8.6	MUC6_HUMAN	1.8
DN20837_c0_g1_i16	#N/A	#N/A	5.72	PTR1_ARATH	0
DN20841_c0_g2_i1	#N/A	#N/A	0.9	SUMO2_ARATH	5.40E-58
DN20846_c0_g1_i10	#N/A	#N/A	-1.2	KEA2_ARATH	3.00E-163
DN20846_c0_g1_i25	#N/A	#N/A	-0.9	KEA2_ARATH	3.00E-175
DN20846_c0_g1_i29	#N/A	#N/A	-1.68	KEA2_ARATH	1.40E-97
DN20847_c0_g1_i5	#N/A	#N/A	9.05	CAP10_ARATH	0
DN20849_c0_g1_i2	#N/A	0.95	#N/A	AMT1_DICDI	0.36
DN20852_c0_g1_i18	#N/A	#N/A	-0.84	TMK1_ARATH	0
DN20859_c0_g2_i1	#N/A	0.65	#N/A	CER3_ARATH	0
DN20860_c0_g1_i6	#N/A	#N/A	-1.13	PPR27_ARATH	1.1
DN20860_c0_g1_i8	#N/A	#N/A	-10.21	PPR27_ARATH	0.97
DN20862_c1_g2_i3	#N/A	#N/A	1.72	CAX3_ARATH	3.00E-101
DN20878_c0_g3_i1	#N/A	#N/A	0.55	ATP1_ARATH	6.30E-87
DN20881_c1_g1_i12	#N/A	7.17	#N/A	IDD5_ARATH	4.00E-69
DN20886_c0_g4_i1	#N/A	#N/A	2.71	MLP43_ARATH	2.80E-24
DN20887_c0_g1_i10	#N/A	#N/A	5.09	SCL33_ARATH	1.00E-164
DN20887_c0_g1_i9	#N/A	#N/A	-1.78	SCL33_ARATH	9.00E-165
DN20893_c0_g1_i27	#N/A	#N/A	10.43	FAAH_MEDTR	0
DN20897_c0_g1_i6	#N/A	-2.56	#N/A	FRO7_ARATH	0
DN20898_c0_g1_i3	#N/A	#N/A	-2.46	PT317_ARATH	0
DN20902_c0_g2_i2	#N/A	-10.8	10.75	FAH_ARATH	0
DN20902_c0_g2_i6	#N/A	-10.59	10.87	FAH_ARATH	0
DN20904_c0_g2_i3	#N/A	#N/A	0.79	CHLM_ARATH	4.00E-163
DN20904_c0_g2_i4	#N/A	#N/A	2.6	CHLM_ARATH	3.00E-162
DN20908_c1_g1_i2	#N/A	-8.32	9.87	HIS8_ARATH	0
DN20910_c1_g1_i12	#N/A	#N/A	1.1	CB5_ARATH	9.20E-58
DN20910_c1_g1_i29	#N/A	#N/A	0.97	CB5_ARATH	6.60E-72
DN20912_c0_g3_i2	#N/A	#N/A	-9.14	HSOP1_SOYBN	0
DN20914_c0_g2_i10	#N/A	#N/A	1.81	USPAL_ARATH	2.10E-16
DN20922_c0_g1_i11	#N/A	#N/A	-0.83	ACA9_ARATH	0
DN20926_c0_g3_i1	#N/A	-0.88	#N/A	HDHD3_BOVIN	1.70E-18
DN20926_c0_g3_i2	#N/A	-1.32	#N/A	HDHD3_BOVIN	4.50E-19
DN20933_c0_g1_i15	#N/A	-1.65	#N/A	IDHP_MEDSA	0
DN20933_c0_g2_i1	#N/A	#N/A	0.58	IDHC_SOYBN	0
DN20938_c0_g1_i7	#N/A	#N/A	1.24	SAP30_HUMAN	0.00021
DN20938_c0_g2_i7	#N/A	7.68	#N/A	CK2_ARATH	5.00E-177

Isoform ID	Log_2(#60tr/nt)	Log_2(#20tr/nt)	Log_2(#60tr/#20t	Annotation_protein	e_value_annotation
DN20944_c1_g3_i5	#N/A	#N/A	-9.05	.	#N/A
DN20945_c0_g1_i8	#N/A	#N/A	-1.03	ARFR_ARATH	0
DN20947_c0_g1_i2	#N/A	-0.82	#N/A	FLZ6_ARATH	3.20E-39
DN20953_c0_g2_i2	#N/A	#N/A	1	RS92_ARATH	3.00E-125
DN20953_c0_g2_i3	#N/A	#N/A	0.95	RS92_ARATH	3.00E-125
DN20957_c1_g1_i1	#N/A	1.75	-2.14	HSP72_SOLLC	0
DN20957_c1_g1_i11	#N/A	1.23	-1.16	HSP7C_PETHY	0
DN20957_c1_g1_i12	#N/A	1.21	#N/A	HSP7C_PETHY	0
DN20957_c1_g2_i2	#N/A	0.86	#N/A	HSP7C_PETHY	1.00E-151
DN20957_c1_g2_i5	#N/A	1.45	#N/A	HSP7C_PETHY	2.00E-120
DN20972_c0_g1_i8	#N/A	-0.88	#N/A	RSZ32_ARATH	2.90E-88
DN20977_c1_g1_i18	#N/A	#N/A	0.76	RS18_THET8	2.30E-10
DN20984_c2_g3_i1	#N/A	#N/A	2.63	CARP1_ARATH	3.00E-32
DN20991_c0_g1_i6	#N/A	#N/A	-1.82	WDR43_HUMAN	3.40E-22
DN20993_c0_g1_i4	#N/A	#N/A	-2.08	CDPKS_ARATH	0
DN20996_c0_g1_i1	#N/A	#N/A	0.59	RCA_ARATH	9.20E-39
DN20996_c1_g2_i13	#N/A	-1.82	0.78	RCA_VIGRR	1.10E-63
DN20996_c1_g2_i16	#N/A	-0.98	#N/A	RCA_VIGRR	4.00E-153
DN20996_c1_g2_i7	#N/A	-0.91	#N/A	RCA2_LARTR	0
DN20996_c1_g2_i9	#N/A	-1.14	#N/A	RCA_VIGRR	0
DN21000_c0_g1_i14	#N/A	#N/A	-0.9	GINT1_ARATH	0
DN21007_c3_g2_i1	#N/A	#N/A	-2.17	POLX_TOBAC	6.00E-125
DN21008_c0_g6_i2	#N/A	#N/A	0.82	.	#N/A
DN21008_c0_g7_i1	#N/A	-1.75	1.32	.	#N/A
DN21008_c0_g7_i2	#N/A	#N/A	0.8	.	#N/A
DN21011_c0_g1_i2	#N/A	7.73	#N/A	.	#N/A
DN21011_c0_g1_i22	#N/A	-8.2	#N/A	.	#N/A
DN21029_c0_g2_i2	#N/A	-1.37	#N/A	SSY1_SOLTU	4.00E-103
DN21038_c0_g1_i9	#N/A	0.95	#N/A	MOS14_ARATH	0
DN21039_c0_g4_i1	#N/A	#N/A	0.79	RS16B_ARATH	1.40E-73
DN21043_c0_g1_i3	#N/A	3.5	#N/A	AB2B_ARATH	0
DN21046_c1_g5_i16	#N/A	#N/A	0.58	DJC76_ARATH	5.40E-72
DN21048_c0_g1_i2	#N/A	-5.86	#N/A	PP209_ARATH	6.00E-168
DN21049_c0_g1_i24	#N/A	#N/A	-0.83	AXS1_ARATH	3.00E-110
DN21057_c0_g1_i4	#N/A	#N/A	-2.16	SCL9_ARATH	0
DN21066_c0_g7_i3	#N/A	1.73	-1.76	GIGAN_ARATH	7.80E-64
DN21068_c0_g1_i15	#N/A	#N/A	-8.23	WDL7_ARATH	3.20E-32
DN21074_c2_g1_i1	#N/A	#N/A	1.16	.	#N/A
DN21074_c2_g1_i8	#N/A	#N/A	0.6	YCF17_PORPU	0.00075

Isoform ID	Log$_2$(#60tr/nt)	Log$_2$(#20tr/nt)	Log$_2$(#60tr/#20t	Annotation_protein	e_value_annotation
DN21076_c0_g2_i8	#N/A	#N/A	5.79	Y1790_ARATH	0
DN21083_c1_g1_i14	#N/A	#N/A	-0.62	RCD1_ARATH	3.00E-114
DN21084_c0_g1_i6	#N/A	#N/A	-2.07	AMY3_ARATH	1.40E-48
DN21089_c0_g3_i2	#N/A	1.5	#N/A	P2B10_ARATH	1.40E-67
DN21089_c0_g3_i4	#N/A	1.75	#N/A	P2B10_ARATH	2.90E-48
DN21094_c1_g10_i2	#N/A	6.78	#N/A	FERON_ARATH	3.00E-129
DN21098_c0_g4_i1	#N/A	#N/A	1.11	RL31_PERFR	5.40E-70
DN21098_c0_g4_i5	#N/A	#N/A	0.81	RL31_PERFR	5.40E-70
DN21099_c1_g1_i13	#N/A	7.59	#N/A	SURE_ESCF3	0.032
DN21101_c0_g1_i16	#N/A	#N/A	0.97	PMT2_ARATH	0
DN21103_c0_g1_i23	#N/A	#N/A	-1.91	SSC14_ARATH	2.00E-115
DN21105_c1_g2_i14	#N/A	-3.07	#N/A	SPL9_ARATH	3.50E-33
DN21109_c0_g1_i21	#N/A	7.63	#N/A	NADK_METST	1.7
DN21112_c0_g1_i19	#N/A	#N/A	-1.17	QSOX2_ARATH	0
DN21125_c0_g1_i1	#N/A	1.47	#N/A	PMTQ_ARATH	0
DN21125_c0_g1_i10	#N/A	-5.48	#N/A	PMTQ_ARATH	0
DN21125_c0_g1_i2	#N/A	-7.14	6.87	PMTQ_ARATH	0
DN21126_c0_g1_i15	#N/A	#N/A	-2.38	CE101_ARATH	1.00E-131
DN21126_c0_g1_i18	#N/A	#N/A	-8.11	CE101_ARATH	1.00E-111
DN21141_c0_g1_i9	#N/A	1.51	#N/A	HSP7Q_ARATH	0
DN21145_c0_g2_i2	#N/A	7.86	#N/A	PRP39_HUMAN	7.90E-38
DN21150_c0_g1_i6	#N/A	-0.91	#N/A	MIEL1_ARATH	2.00E-171
DN21157_c1_g1_i3	#N/A	8.05	#N/A	ICR2_ARATH	6.00E-171
DN21167_c1_g1_i10	#N/A	#N/A	-0.75	TBL10_ARATH	5.00E-167
DN21174_c0_g1_i8	#N/A	9.08	#N/A	VPS51_ARATH	0
DN21178_c0_g1_i4	#N/A	8.52	#N/A	AAP7_ARATH	9.30E-52
DN21179_c0_g2_i5	#N/A	1.92	#N/A	Y5241_ARATH	0
DN21190_c0_g2_i11	#N/A	#N/A	-1.44	Y4291_ARATH	0
DN21193_c0_g2_i2	#N/A	#N/A	0.84	VAC14_MOUSE	1.7
DN21207_c0_g1_i6	#N/A	-0.92	#N/A	LOGL6_ORYSJ	6.00E-113
DN21210_c1_g5_i5	#N/A	#N/A	-1.04	PPO_MALDO	5.00E-177
DN21219_c0_g1_i19	#N/A	9.21	-9.35	LRK10_WHEAT	3.00E-108
DN21219_c0_g1_i3	#N/A	#N/A	-1.44	LRK10_WHEAT	2.10E-65
DN21223_c0_g8_i2	#N/A	#N/A	0.91	.	#N/A
DN21224_c0_g3_i3	#N/A	9.34	#N/A	RBL12_ARATH	3.00E-104
DN21230_c1_g1_i12	#N/A	#N/A	-8.84	IDD10_ARATH	3.80E-12
DN21237_c0_g1_i7	#N/A	-10.01	#N/A	FUBP1_MOUSE	2.60E-06
DN21252_c0_g1_i8	#N/A	#N/A	-5.53	.	#N/A
DN21257_c0_g1_i12	#N/A	#N/A	-7.91	CTPA2_ARATH	1.00E-126

Isoform ID	Log$_2$(#60tr/nt)	Log$_2$(#20tr/nt)	Log$_2$(#60tr/#20t	Annotation_protein	e_value_annotation
DN21263_c1_g1_i11	#N/A	#N/A	-0.64	SERK4_ORYSJ	0
DN21267_c1_g1_i5	#N/A	#N/A	-0.67	HSP7M_PHAVU	0
DN21268_c0_g2_i6	#N/A	#N/A	-7.68	FABI_ENTFA	2.7
DN21268_c0_g4_i1	#N/A	#N/A	0.8	.	#N/A
DN21268_c0_g5_i1	#N/A	#N/A	0.68	HSP16_SOYBN	1.70E-26
DN21271_c0_g2_i4	#N/A	-1.09	1.07	.	#N/A
DN21273_c0_g2_i13	#N/A	#N/A	-1.86	PUB12_ORYSJ	5.80E-08
DN21274_c1_g1_i2	#N/A	1.02	#N/A	C3H30_ARATH	0
DN21287_c0_g1_i3	#N/A	#N/A	0.85	RK18_SPIOL	1.20E-73
DN21291_c1_g1_i2	#N/A	-10.41	#N/A	SCP20_ARATH	0
DN21294_c0_g1_i10	#N/A	7.61	#N/A	CRLK1_ARATH	0
DN21307_c0_g2_i23	#N/A	#N/A	-3.74	CRK25_ARATH	6.00E-89
DN21309_c0_g1_i16	#N/A	#N/A	-7.7	PSS1_ARATH	0
DN21309_c0_g1_i26	#N/A	#N/A	-8.68	PSS1_ARATH	2.00E-161
DN21312_c1_g3_i1	#N/A	#N/A	-6.83	STY13_ARATH	4.40E-40
DN21313_c0_g4_i5	#N/A	8.35	#N/A	SIPL2_ARATH	1.80E-81
DN21319_c1_g1_i6	#N/A	#N/A	-2.92	ROQ1_NICBE	5.00E-110
DN21322_c0_g2_i11	#N/A	#N/A	-1.96	AUG8_ARATH	2.00E-178
DN21328_c0_g1_i6	#N/A	#N/A	-0.69	ECT2_ARATH	9.90E-26
DN21342_c0_g1_i22	#N/A	#N/A	-8.98	PMT1_ARATH	0
DN21344_c0_g1_i22	#N/A	#N/A	-8.68	C3H5_ARATH	1.50E-70
DN21362_c0_g1_i2	#N/A	1.77	#N/A	GDL86_ARATH	1.00E-142
DN21362_c0_g1_i3	#N/A	-8.25	#N/A	GDL86_ARATH	3.00E-128
DN21370_c0_g2_i3	#N/A	7.17	#N/A	DMS3_ARATH	8.80E-52
DN21386_c0_g3_i7	#N/A	-8.21	#N/A	MLN51_ARATH	3.10E-80
DN21387_c0_g1_i11	#N/A	#N/A	-9.98	P2C05_ARATH	1.40E-60
DN21387_c0_g1_i14	#N/A	#N/A	8.2	P2C05_ARATH	0
DN21387_c0_g1_i2	#N/A	#N/A	5.51	P2C05_ARATH	5.00E-115
DN21388_c1_g1_i9	#N/A	0.99	#N/A	IQD31_ARATH	4.00E-118
DN21394_c0_g3_i2	#N/A	#N/A	3.67	BH025_ARATH	3.90E-36
DN21395_c1_g1_i7	#N/A	5.74	#N/A	Y3078_ARATH	0
DN21399_c0_g1_i8	#N/A	#N/A	0.94	.	#N/A
DN21404_c0_g2_i12	#N/A	#N/A	-1.75	PLY22_ARATH	0
DN21406_c1_g2_i5	#N/A	#N/A	0.82	SC61B_ARATH	1.10E-31
DN21406_c1_g2_i7	#N/A	#N/A	0.73	SC61B_ARATH	1.10E-31
DN21416_c0_g1_i1	#N/A	#N/A	0.8	PPL1_ARATH	2.00E-59
DN21422_c1_g1_i6	#N/A	0.96	#N/A	TMN11_ARATH	0
DN21431_c0_g5_i2	#N/A	-0.9	#N/A	TRNHF_ARATH	1.50E-37
DN21432_c0_g2_i3	#N/A	#N/A	0.78	.	#N/A

Isoform ID	Log_2(#60tr/nt)	Log_2(#20tr/nt)	Log_2(#60tr/#20t	Annotation_protein	e_value_annotation
DN21440_c0_g2_i3	#N/A	#N/A	1.14	FLZ14_ARATH	9.40E-45
DN21444_c0_g1_i11	#N/A	#N/A	2	.	#N/A
DN21444_c0_g1_i20	#N/A	-1.85	#N/A	SSL4_ARATH	2.60E-49
DN21449_c1_g1_i11	#N/A	#N/A	-7.82	PHF3_HUMAN	0.008
DN21450_c0_g3_i10	#N/A	#N/A	0.87	MDIS2_ARATH	7.00E-168
DN21450_c0_g3_i3	#N/A	#N/A	-0.83	MDIS2_ARATH	7.00E-168
DN21450_c0_g3_i4	#N/A	1.08	#N/A	MDIS2_ARATH	4.00E-168
DN21450_c0_g3_i8	#N/A	#N/A	5.7	MDIS2_ARATH	1.00E-165
DN21450_c0_g3_i9	#N/A	-10.78	#N/A	MDIS2_ARATH	2.20E-46
DN21459_c0_g1_i7	#N/A	#N/A	1.14	FAO2_LOTJA	1.00E-123
DN21470_c1_g2_i6	#N/A	7.54	#N/A	IBS1_ARATH	0
DN21488_c0_g1_i3	#N/A	9.39	-9.52	FPP7_ARATH	3.50E-93
DN21496_c0_g1_i21	#N/A	#N/A	-7.59	CTF50_ARATH	0
DN21499_c1_g2_i2	#N/A	#N/A	-0.7	.	#N/A
DN21502_c0_g1_i13	#N/A	#N/A	-0.9	SHT_ARATH	2.10E-69
DN21506_c0_g1_i16	#N/A	#N/A	-0.74	NU4LM_ECHGY	0.61
DN21511_c0_g3_i2	#N/A	#N/A	1.45	RL121_ARATH	2.40E-59
DN21511_c0_g3_i3	#N/A	#N/A	0.92	RL121_ARATH	2.40E-59
DN21522_c1_g1_i2	#N/A	#N/A	-1.12	CIP7_ARATH	3.00E-124
DN21522_c1_g1_i9	#N/A	1.42	#N/A	CIP7_ARATH	2.00E-128
DN21523_c1_g1_i7	#N/A	9.18	-9.31	EBP1_SOLTU	4.00E-168
DN21531_c0_g1_i2	#N/A	#N/A	0.85	DRE2A_ORYSI	1.30E-49
DN21534_c0_g1_i10	#N/A	#N/A	-1.05	DRL19_ARATH	0.007
DN21538_c1_g1_i1	#N/A	#N/A	-0.95	ZFP34_ORYSJ	2.30E-63
DN21538_c1_g1_i6	#N/A	#N/A	-0.73	ZFP34_ORYSJ	4.50E-92
DN21549_c0_g1_i10	#N/A	-8.75	8.99	UBAC1_HUMAN	0.35
DN21555_c0_g3_i1	#N/A	#N/A	-7.9	KN4A_ARATH	5.00E-148
DN21556_c1_g1_i7	#N/A	-0.94	#N/A	RGLG4_ARATH	0
DN21557_c0_g1_i15	#N/A	2.77	#N/A	.	#N/A
DN21562_c0_g2_i7	#N/A	-9.4	#N/A	ZDH22_ARATH	0
DN21563_c0_g2_i5	#N/A	-5.46	#N/A	.	#N/A
DN21575_c0_g1_i2	#N/A	-0.83	#N/A	PYRC5_PYRCO	0
DN21578_c1_g3_i4	#N/A	#N/A	1.19	.	#N/A
DN21584_c0_g3_i4	#N/A	#N/A	-0.78	SMC1_ARATH	0
DN21585_c0_g1_i7	#N/A	7.83	#N/A	XPOT_ARATH	0
DN21588_c0_g1_i16	#N/A	2.58	-1.98	LRL14_ARATH	2.30E-88
DN21588_c0_g1_i9	#N/A	9.55	-6.61	LRL14_ARATH	1.90E-91
DN21590_c0_g1_i4	#N/A	9	#N/A	TRM32_ARATH	4.60E-27
DN21592_c0_g1_i4	#N/A	#N/A	1.6	LGT_MYCA9	0.044

Isoform ID	Log_2(#60tr/nt)	Log_2(#20tr/nt)	Log_2(#60tr/#20t	Annotation_protein	e_value_annotation
DN21597_c0_g1_i19	#N/A	#N/A	10.58	ERG14_ARATH	8.00E-150
DN21597_c0_g1_i5	#N/A	#N/A	0.86	SQE1_PANGI	0
DN21597_c0_g1_i8	#N/A	#N/A	1.27	SQE1_PANGI	0
DN21612_c1_g2_i9	#N/A	1.46	#N/A	GPMI_PROMP	4
DN21613_c0_g1_i8	#N/A	#N/A	-2.86	PABP2_ARATH	0
DN21618_c0_g1_i17	#N/A	1.17	#N/A	DJB12_MOUSE	2.60E-13
DN21626_c1_g1_i1	#N/A	#N/A	-1.28	CHR4_ARATH	0
DN21628_c0_g1_i1	#N/A	#N/A	0.78	PSRP5_PEA	7.80E-33
DN21632_c0_g6_i1	#N/A	#N/A	1.01	.	#N/A
DN21633_c0_g1_i23	#N/A	#N/A	-1.19	VW5B1_MOUSE	0.00076
DN21649_c1_g2_i6	#N/A	#N/A	1.62	Y1960_ARATH	0
DN21668_c0_g3_i16	#N/A	-1.65	#N/A	HD6_ORYSJ	7.80E-94
DN21677_c1_g1_i13	#N/A	#N/A	-8.48	GAUT6_ARATH	2.00E-168
DN21680_c1_g4_i1	#N/A	#N/A	-2.03	PKL_ARATH	0
DN21683_c1_g2_i9	#N/A	#N/A	-8.27	PCFS4_ARATH	2.70E-53
DN21694_c0_g1_i20	#N/A	7.82	#N/A	PRD15_MOUSE	0.085
DN21708_c0_g1_i25	#N/A	#N/A	-8.72	POLL2_ARATH	0
DN21710_c0_g1_i22	#N/A	#N/A	-0.84	PP395_ARATH	0
DN21713_c0_g1_i1	#N/A	#N/A	-1.02	SUD1_ARATH	0
DN21730_c0_g1_i19	#N/A	#N/A	0.62	.	#N/A
DN21733_c1_g1_i10	#N/A	1.6	#N/A	KN7E_ARATH	0
DN21746_c2_g1_i9	#N/A	2.1	#N/A	CDKF4_ORYSJ	0
DN21748_c1_g1_i4	#N/A	#N/A	-0.9	XB33_ARATH	0
DN21752_c1_g1_i16	#N/A	#N/A	-2.21	SPA3_ARATH	0
DN21757_c0_g1_i2	#N/A	0.91	#N/A	Y2165_ARATH	0
DN21757_c0_g1_i7	#N/A	#N/A	-1.09	Y2165_ARATH	0
DN21776_c0_g2_i2	#N/A	#N/A	-1.28	IF2C_PHAVU	0
DN21779_c0_g1_i2	#N/A	#N/A	-0.7	Y005_SYNY3	3.00E-142
DN21784_c0_g2_i13	#N/A	#N/A	-1	KEA3_ARATH	0
DN21788_c1_g1_i14	#N/A	#N/A	-0.69	ACA1_ARATH	0
DN21789_c1_g1_i21	#N/A	#N/A	-8.63	PSA_ARATH	0
DN21790_c0_g1_i1	#N/A	#N/A	-0.99	ACA2_ARATH	0
DN21790_c0_g1_i10	#N/A	1.24	-1.11	ACA2_ARATH	0
DN21802_c0_g1_i10	#N/A	#N/A	-1.77	TSS_ARATH	0
DN21802_c0_g3_i3	#N/A	#N/A	1.38	CSLE1_ORYSJ	1.10E-77
DN21804_c1_g3_i3	#N/A	#N/A	1.08	HYPK_MOUSE	2.00E-10
DN21812_c0_g2_i4	#N/A	#N/A	-0.6	DPE2_ARATH	0
DN21812_c0_g2_i8	#N/A	#N/A	-10.59	DPE2_ARATH	0
DN21814_c0_g3_i2	#N/A	10.54	#N/A	SKI11_ARATH	6.00E-156

Isoform ID	Log_2(#60tr/nt)	Log_2(#20tr/nt)	Log_2(#60tr/#20t	Annotation_protein	e_value_annotation
DN21814_c0_g3_i3	#N/A	9.01	#N/A	SKI11_ARATH	6.00E-156
DN21819_c0_g2_i17	#N/A	1.68	#N/A	PMT8_ARATH	0
DN21830_c0_g2_i20	#N/A	#N/A	-1.4	Y6689_DICDI	1.40E-70
DN21834_c1_g1_i11	#N/A	-0.97	#N/A	WTR42_ARATH	3.50E-78
DN21846_c0_g1_i20	#N/A	#N/A	2	GRP1_SINAL	3.30E-49
DN21846_c0_g1_i24	#N/A	#N/A	0.81	GRP1_SINAL	9.20E-45
DN21846_c0_g1_i43	#N/A	#N/A	1.03	GRP1_SINAL	8.20E-43
DN21846_c0_g1_i8	#N/A	#N/A	0.64	GRP1_SINAL	2.70E-47
DN21854_c1_g1_i9	#N/A	#N/A	0.5	ARC_JONDD	1.2
DN21856_c0_g2_i2	#N/A	#N/A	-0.69	INO1_TOBAC	0
DN21862_c0_g2_i3	#N/A	#N/A	-7.16	AN13B_HUMAN	8.10E-31
DN21871_c0_g1_i1	#N/A	#N/A	-1.12	RIC1_DANRE	2.00E-36
DN21880_c0_g2_i9	#N/A	#N/A	-2.5	GLR34_ARATH	0
DN21892_c1_g6_i1	#N/A	-1.65	#N/A	NORR_ENT38	0.85
DN21895_c1_g2_i1	#N/A	#N/A	9.91	ACR4L_ARATH	0
DN21895_c1_g2_i4	#N/A	#N/A	-9.9	ACR4L_ARATH	0
DN21896_c0_g1_i6	#N/A	#N/A	0.83	NDA8B_ARATH	9.80E-62
DN21909_c1_g1_i7	#N/A	#N/A	-1.03	AB10C_ARATH	0
DN21911_c0_g1_i11	#N/A	0.88	#N/A	Y3037_ARATH	0
DN21916_c0_g1_i16	#N/A	-4.89	#N/A	Y1559_ARATH	0
DN21916_c0_g1_i6	#N/A	#N/A	-3.08	Y1559_ARATH	1.00E-155
DN21919_c1_g4_i5	#N/A	#N/A	-8.1	NIPLB_DANRE	3.90E-05
DN21924_c0_g2_i4	#N/A	#N/A	-1.21	LNK2_ARATH	5.50E-25
DN21929_c1_g2_i5	#N/A	-0.71	#N/A	VPE_VICSA	2.20E-30
DN21932_c0_g1_i1	#N/A	#N/A	-0.7	SPA1_ARATH	0
DN21932_c0_g1_i4	#N/A	#N/A	-0.57	SPA1_ARATH	1.00E-96
DN21932_c0_g1_i5	#N/A	#N/A	-1.34	SPA1_ARATH	1.00E-161
DN21932_c0_g1_i9	#N/A	#N/A	-1.61	SPA1_ARATH	3.00E-162
DN21936_c0_g1_i6	#N/A	#N/A	-1.95	PI5K8_ARATH	0
DN21939_c0_g2_i5	#N/A	#N/A	2.07	DRL28_ARATH	0.00047
DN21940_c0_g2_i4	#N/A	#N/A	-1.51	M3K20_ARATH	5.00E-111
DN21949_c0_g1_i10	#N/A	0.92	#N/A	HSP83_IPONI	2.00E-157
DN21949_c0_g1_i2	#N/A	0.73	#N/A	HSP80_SOLLC	0
DN21949_c0_g1_i3	#N/A	0.53	#N/A	HSP80_SOLLC	0
DN21950_c0_g1_i7	#N/A	0.71	#N/A	IPO5_MOUSE	0
DN21951_c0_g1_i4	#N/A	-7.73	#N/A	WDR44_BOVIN	5.80E-51
DN21951_c0_g1_i8	#N/A	8.74	-8.88	WDR44_BOVIN	5.80E-51
DN21957_c0_g3_i1	#N/A	#N/A	1.32	SCGT_TOBAC	1.00E-123
DN21957_c1_g1_i1	#N/A	#N/A	-1.83	TIC_ARATH	2.00E-173

Isoform ID	Log_2(#60tr/nt)	Log_2(#20tr/nt)	Log_2(#60tr/#20t	Annotation_protein	e_value_annotation
DN21958_c0_g1_i3	#N/A	#N/A	-8.26	NPY2_ARATH	1.00E-147
DN21961_c0_g1_i3	#N/A	1.54	-1.91	HSF30_SOLPE	2.00E-125
DN21961_c0_g1_i6	#N/A	1.82	#N/A	HSF30_SOLPE	2.00E-125
DN21965_c0_g1_i3	#N/A	2.27	#N/A	Y5673_ARATH	0
DN21965_c0_g1_i7	#N/A	#N/A	5.05	Y5673_ARATH	0
DN21967_c1_g1_i3	#N/A	1.36	-1.78	CMOA_ARCB4	2.5
DN21967_c1_g1_i8	#N/A	#N/A	-2.07	POLX_TOBAC	8.10E-17
DN21968_c0_g1_i13	#N/A	#N/A	-1.23	PHOT2_ARATH	0
DN21968_c0_g1_i3	#N/A	#N/A	-0.85	PHOT2_ARATH	0
DN21968_c0_g1_i5	#N/A	#N/A	-0.81	PHOT2_ARATH	0
DN21975_c0_g2_i9	#N/A	#N/A	8.09	RPC4_MOUSE	1.10E-07
DN21978_c0_g1_i3	#N/A	#N/A	2.5	.	#N/A
DN21980_c0_g1_i1	#N/A	-1.33	#N/A	CXXS1_ARATH	3.40E-51
DN21985_c0_g1_i5	#N/A	-8.24	#N/A	KMT2B_HUMAN	3.80E-27
DN22002_c2_g1_i6	#N/A	#N/A	-2.08	SYRM_ARATH	0
DN22004_c0_g1_i13	#N/A	#N/A	-0.79	PHSH_VICFA	6.00E-179
DN22004_c0_g1_i15	#N/A	#N/A	-0.85	PHSH_VICFA	0
DN22004_c0_g1_i9	#N/A	#N/A	-0.68	PHSH_VICFA	0
DN22016_c1_g1_i16	#N/A	#N/A	5.06	GLNA4_PHAVU	0
DN22020_c0_g1_i1	#N/A	#N/A	0.84	RK4_TOBAC	1.00E-112
DN22020_c0_g1_i2	#N/A	#N/A	0.82	RK4_TOBAC	2.00E-112
DN22030_c0_g1_i1	#N/A	#N/A	11.1	PX11B_ARATH	7.00E-116
DN22032_c0_g2_i12	#N/A	#N/A	8.13	B561A_ARATH	0
DN22033_c1_g2_i5	#N/A	#N/A	-1.54	.	#N/A
DN22044_c0_g1_i1	#N/A	#N/A	1.37	SYCE1_MOUSE	3.2
DN22045_c0_g1_i18	#N/A	7.72	#N/A	MRF1_ARATH	0
DN22045_c0_g1_i19	#N/A	-7.8	#N/A	MRF1_ARATH	0
DN22045_c0_g1_i4	#N/A	#N/A	5.73	MRF1_ARATH	0
DN22045_c0_g1_i6	#N/A	4.81	#N/A	DPO1_CALBD	1.5
DN22045_c0_g2_i6	#N/A	1.02	#N/A	CDPKK_ORYSJ	3.00E-125
DN22049_c0_g1_i2	#N/A	#N/A	4.22	HEL2_SCHPO	1.80E-42
DN22052_c0_g3_i2	#N/A	#N/A	-1.95	C3H55_ORYSJ	3.70E-14
DN22054_c0_g1_i10	#N/A	8.78	#N/A	KINUA_ARATH	0
DN22055_c0_g1_i11	#N/A	#N/A	-7.48	LNG1_ARATH	7.60E-85
DN22055_c0_g1_i3	#N/A	#N/A	-7.98	LNG1_ARATH	7.60E-85
DN22056_c0_g3_i1	#N/A	2.64	#N/A	GLR36_ARATH	0
DN22057_c0_g1_i3	#N/A	9.16	#N/A	PSKR2_ARATH	0
DN22057_c0_g1_i4	#N/A	7.32	#N/A	PSKR2_ARATH	0
DN22077_c0_g1_i19	#N/A	-8.92	#N/A	BAMC_SHESH	0.068

Isoform ID	Log_2(#60tr/nt)	Log_2(#20tr/nt)	Log_2(#60tr/#20t	Annotation_protein	e_value_annotation
DN22080_c1_g1_i14	#N/A	-8.18	#N/A	SWI3C_ARATH	2.50E-41
DN22086_c0_g2_i4	#N/A	#N/A	0.84	KAD2_ARATH	1.00E-123
DN22090_c1_g2_i5	#N/A	#N/A	-8.34	BLH1_ARATH	4.00E-121
DN22102_c0_g1_i13	#N/A	#N/A	-10.28	FRAY2_DICDI	3.00E-115
DN22102_c0_g1_i22	#N/A	#N/A	-10.02	FRAY2_DICDI	5.00E-115
DN22112_c0_g1_i12	#N/A	#N/A	-10.32	TEX10_CHICK	1.80E-13
DN22114_c1_g1_i12	#N/A	#N/A	7.96	KN12B_ARATH	0.004
DN22115_c0_g1_i7	#N/A	-0.7	#N/A	DDPS2_ARATH	6.00E-128
DN22117_c0_g1_i2	#N/A	8.51	#N/A	WAPL1_ARATH	0
DN22123_c0_g2_i18	#N/A	#N/A	-1.51	BLH1_ARATH	4.00E-166
DN22124_c1_g2_i14	#N/A	#N/A	-8.97	RH37_ARATH	0
DN22126_c1_g1_i1	#N/A	#N/A	-0.98	MPK20_ARATH	0
DN22126_c1_g1_i18	#N/A	1.27	#N/A	MPK10_ORYSJ	0
DN22127_c0_g2_i11	#N/A	#N/A	-3.91	FRS6_ARATH	1.00E-108
DN22131_c0_g3_i1	#N/A	#N/A	-1.66	UPL3_ARATH	0
DN22133_c0_g1_i8	#N/A	#N/A	-1.23	MTR4_ARATH	0
DN22134_c1_g2_i7	#N/A	#N/A	-0.69	KTN82_ARATH	0
DN22136_c0_g2_i3	#N/A	#N/A	-10.57	MFPA_CUCSA	0
DN22151_c0_g1_i7	#N/A	#N/A	1.4	PMTK_ARATH	0
DN22164_c0_g1_i6	#N/A	#N/A	-1.13	.	#N/A
DN22172_c0_g1_i4	#N/A	2.36	#N/A	ACCA_ARATH	0
DN22175_c0_g3_i3	#N/A	#N/A	-1.01	TPS5_ARATH	0
DN22183_c1_g1_i16	#N/A	-8.27	#N/A	FUCO2_ARATH	1.00E-163
DN22184_c0_g3_i13	#N/A	#N/A	-1.47	LHY_ARATH	6.00E-124
DN22194_c1_g6_i1	#N/A	#N/A	1.12	SGT2_SOYBN	0
DN22194_c1_g7_i1	#N/A	#N/A	1.16	SGT2_SOYBN	0
DN22194_c1_g7_i2	#N/A	#N/A	1.61	SGT2_SOYBN	2.00E-161
DN22196_c1_g1_i1	#N/A	-1	#N/A	HIP26_ARATH	1.30E-82
DN22196_c1_g2_i2	#N/A	-5.56	#N/A	APC1_ARATH	1.00E-175
DN22198_c0_g1_i18	#N/A	1.59	#N/A	KN7G_ARATH	0
DN22201_c0_g1_i1	#N/A	#N/A	2.31	L_HENDH	2.6
DN22201_c0_g1_i10	#N/A	#N/A	2.08	LOX4_SOYBN	0
DN22201_c0_g1_i4	#N/A	#N/A	1.33	LOX4_SOYBN	0
DN22201_c0_g1_i8	#N/A	#N/A	1.21	LOX4_SOYBN	0
DN22204_c1_g1_i3	#N/A	#N/A	-0.84	SIS8_ARATH	9.90E-50
DN22204_c1_g1_i5	#N/A	#N/A	-1.02	SIS8_ARATH	9.10E-50
DN22210_c0_g1_i2	#N/A	1.78	#N/A	AB9C_ARATH	0
DN22210_c0_g1_i21	#N/A	3.33	#N/A	AB9C_ARATH	0
DN22217_c0_g1_i13	#N/A	#N/A	-1.27	RLT1_ARATH	0

Isoform ID	Log_2(#60tr/nt)	Log_2(#20tr/nt)	Log_2(#60tr/#20t	Annotation_protein	e_value_annotation
DN22219_c1_g1_i3	#N/A	-1.04	#N/A	.	#N/A
DN22219_c1_g1_i4	#N/A	-0.64	#N/A	.	#N/A
DN22219_c2_g1_i4	#N/A	#N/A	-1.27	ALA3_ARATH	0
DN22220_c2_g4_i1	#N/A	0.94	#N/A	SMXL7_ARATH	0
DN22220_c2_g4_i7	#N/A	-1.28	#N/A	SMXL7_ARATH	0
DN22228_c1_g3_i1	#N/A	#N/A	0.86	EPS1_ARATH	2.40E-97
DN22249_c1_g1_i6	#N/A	#N/A	-0.78	GIGAN_ARATH	0
DN22251_c0_g1_i10	#N/A	#N/A	4.82	.	#N/A
DN22252_c1_g3_i14	#N/A	2.49	-2.03	RPV1_VITRO	1.10E-16
DN22262_c0_g1_i9	#N/A	0.98	#N/A	.	#N/A
DN22266_c0_g1_i6	#N/A	#N/A	-1.21	SCGT_TOBAC	0
DN22271_c0_g1_i4	#N/A	#N/A	-5.11	CNGC1_ARATH	3.00E-117
DN22288_c1_g3_i4	#N/A	#N/A	1.21	SET1_ASHGO	0.53
DN22291_c0_g2_i13	#N/A	8.79	#N/A	IP5P3_ARATH	6.40E-97
DN22291_c1_g1_i2	#N/A	#N/A	2.23	IP5P3_ARATH	1.00E-109
DN22291_c1_g1_i4	#N/A	#N/A	-8.76	IP5P3_ARATH	3.00E-161
DN22293_c0_g1_i9	#N/A	9.24	-9.38	ACA4_ARATH	0
DN22294_c0_g1_i14	#N/A	#N/A	-9.24	SLK2_ARATH	0
DN22295_c0_g3_i1	#N/A	#N/A	-0.89	BOR1_ARATH	0
DN22295_c0_g3_i6	#N/A	#N/A	-3.62	.	#N/A
DN22297_c1_g2_i7	#N/A	#N/A	-4.11	UVR3_ARATH	9.10E-81
DN22298_c1_g2_i1	#N/A	#N/A	-0.72	VILI4_ARATH	0
DN22300_c0_g4_i1	#N/A	1.94	#N/A	.	#N/A
DN22310_c0_g2_i8	#N/A	0.91	#N/A	TMN9_ARATH	0
DN22311_c0_g1_i1	#N/A	1.39	-0.98	ACA12_ARATH	0
DN22311_c0_g1_i8	#N/A	1.32	#N/A	ACA12_ARATH	0
DN22327_c0_g1_i1	#N/A	#N/A	-0.74	.	#N/A
DN22331_c0_g1_i2	#N/A	8.68	#N/A	XRN4_ARATH	2.70E-88
DN22337_c0_g1_i10	#N/A	#N/A	7.52	.	#N/A
DN22337_c0_g1_i3	#N/A	#N/A	1.62	.	#N/A
DN22337_c0_g1_i5	#N/A	#N/A	2.83	.	#N/A
DN22340_c0_g1_i8	#N/A	#N/A	-0.91	SBT25_ARATH	0
DN22341_c0_g4_i1	#N/A	#N/A	-0.84	UPL7_ARATH	0
DN22342_c0_g1_i1	#N/A	#N/A	-2.94	UPL1_ARATH	0
DN22342_c0_g1_i2	#N/A	#N/A	-1.7	UPL1_ARATH	0
DN22351_c1_g1_i8	#N/A	0.97	#N/A	ERECT_ARATH	0
DN22356_c0_g2_i4	#N/A	#N/A	-7.3	.	#N/A
DN22359_c0_g1_i14	#N/A	#N/A	-0.79	CHR5_ARATH	0
DN22367_c0_g1_i15	#N/A	#N/A	-1.07	PHOT1_ARATH	0

Isoform ID	Log_2(#60tr/nt)	Log_2(#20tr/nt)	Log_2(#60tr/#20t	Annotation_protein	e_value_annotation
DN22367_c0_g2_i7	#N/A	#N/A	-1.86	PHOT1_ARATH	2.00E-125
DN22369_c1_g1_i10	#N/A	#N/A	-8.38	ERO2_ARATH	5.00E-128
DN22371_c0_g1_i11	#N/A	1.01	#N/A	AML4_ARATH	3.00E-165
DN22372_c0_g2_i1	#N/A	#N/A	-1.87	SSY3_SOLTU	0
DN22372_c0_g2_i4	#N/A	#N/A	-1.47	SSY3_SOLTU	0
DN22372_c0_g2_i8	#N/A	#N/A	-1.18	SSY3_SOLTU	0
DN22377_c0_g1_i11	#N/A	#N/A	-10.79	CDKG2_ORYSJ	0
DN22390_c1_g2_i7	#N/A	#N/A	-1.12	PP6R2_MOUSE	6.10E-55
DN22390_c1_g5_i5	#N/A	10.93	-11.07	PACN2_HUMAN	0.7
DN22397_c0_g3_i2	#N/A	#N/A	-1.15	SMG7_ARATH	0
DN22411_c0_g1_i19	#N/A	#N/A	-1.26	SPP_PEA	0
DN22420_c1_g1_i12	#N/A	#N/A	-0.94	GCSP_PEA	0
DN22420_c1_g1_i14	#N/A	#N/A	-0.89	GCSP_PEA	0
DN22420_c1_g1_i15	#N/A	#N/A	-0.58	GCSP_PEA	0
DN22420_c1_g1_i4	#N/A	#N/A	-0.74	GCSP_PEA	0
DN22420_c1_g1_i8	#N/A	#N/A	-0.93	GCSP_PEA	0
DN22427_c0_g1_i16	#N/A	#N/A	4.77	TRPA_BACC0	0.76
DN22434_c2_g1_i16	#N/A	#N/A	-1.59	.	#N/A
DN22436_c1_g2_i7	#N/A	#N/A	-0.93	TPPII_ARATH	0
DN22443_c1_g1_i5	#N/A	#N/A	-0.91	CAPP4_ARATH	0
DN22456_c2_g1_i13	#N/A	#N/A	-0.64	CLPC_PEA	0
DN22456_c2_g1_i3	#N/A	#N/A	-0.83	CLPC_PEA	0
DN22456_c2_g1_i4	#N/A	#N/A	-0.67	CLPC_PEA	5.00E-105
DN22456_c2_g2_i1	#N/A	#N/A	-0.81	CLPC_PEA	7.80E-67
DN22456_c2_g2_i2	#N/A	#N/A	-0.82	CLPC_PEA	2.70E-66
DN22465_c0_g1_i2	#N/A	#N/A	-1.25	MIP2_ARATH	0
DN22467_c0_g1_i1	#N/A	#N/A	-1.44	BRWD1_HUMAN	5.00E-78
DN22472_c0_g2_i12	#N/A	#N/A	-0.94	EIN2_ARATH	0
DN22479_c0_g1_i8	#N/A	#N/A	-1.05	POLX_TOBAC	2.50E-88
DN22480_c0_g1_i12	#N/A	#N/A	-1.23	MYO6_ARATH	0
DN22482_c1_g1_i17	#N/A	#N/A	1.33	APO2_ARATH	8.70E-70
DN22488_c0_g2_i8	#N/A	#N/A	-1.15	SNL2_ARATH	0
DN22495_c1_g1_i9	#N/A	1.48	#N/A	AGUA_SELRU	0.26
DN22496_c0_g1_i22	#N/A	#N/A	-1.54	CNG20_ARATH	0
DN22504_c1_g1_i1	#N/A	#N/A	-8.59	UBP26_ARATH	0
DN22504_c1_g1_i8	#N/A	#N/A	-9.45	UBP26_ARATH	0
DN22507_c1_g3_i4	#N/A	#N/A	-0.78	SRL2_ORYSJ	2.40E-72
DN22511_c0_g1_i5	#N/A	#N/A	-2.5	PDR1_PETHY	0
DN22511_c1_g1_i3	#N/A	1.5	-1.76	AB32G_ARATH	0

Isoform ID	Log_2(#60tr/nt)	Log_2(#20tr/nt)	Log_2(#60tr/#20t	Annotation_protein	e_value_annotation
DN22511_c1_g1_i5	#N/A	0.95	#N/A	AB32G_ARATH	0
DN22512_c0_g2_i14	#N/A	#N/A	-0.99	UPL6_ARATH	0
DN22516_c0_g1_i21	#N/A	#N/A	-1.46	CALS9_ARATH	0
DN22518_c1_g2_i18	#N/A	#N/A	-6.19	DEAH2_ARATH	0
DN22518_c1_g2_i5	#N/A	#N/A	-5.4	DEAH2_ARATH	0
DN22519_c0_g1_i2	#N/A	0.74	#N/A	CESA9_ARATH	0
DN22519_c0_g1_i3	#N/A	1.15	#N/A	CESA9_ARATH	0
DN22519_c0_g2_i13	#N/A	1.22	#N/A	CESA6_ARATH	0
DN22527_c1_g1_i6	#N/A	#N/A	1.18	PPA27_ARATH	0
DN22528_c1_g1_i4	#N/A	2.15	#N/A	DEXH3_ARATH	0
DN22533_c1_g1_i15	#N/A	#N/A	-1.72	ACOC_CUCMA	0
DN22534_c1_g1_i3	#N/A	#N/A	-0.9	TPR3_ARATH	0
DN22540_c1_g2_i3	#N/A	#N/A	-8.81	CNOT4_HUMAN	2.40E-36
DN22543_c1_g1_i8	#N/A	#N/A	-1.04	GWD1_CITRE	0
DN22548_c0_g1_i1	#N/A	#N/A	-1.01	GLTB1_ARATH	0
DN22548_c0_g1_i10	#N/A	#N/A	-1.42	GLTB1_ARATH	0
DN22548_c0_g1_i7	#N/A	#N/A	-0.97	GLTB1_ARATH	0
DN22552_c0_g1_i8	#N/A	6.33	#N/A	NRPA1_ARATH	5.00E-167
DN22554_c1_g2_i9	#N/A	#N/A	-10.53	ALE2_ARATH	0
DN22556_c0_g4_i1	#N/A	#N/A	0.87	.	#N/A
DN22557_c0_g1_i12	#N/A	#N/A	-1.64	Y1439_ARATH	0.39
DN22557_c0_g1_i20	#N/A	#N/A	9.37	Y1439_ARATH	0.39
DN22557_c0_g1_i3	#N/A	#N/A	-6.44	Y1439_ARATH	0.39
DN22558_c0_g1_i6	#N/A	#N/A	-7.84	ROS1_ARATH	0
DN22560_c0_g1_i3	#N/A	#N/A	-1.43	CHLB_SYNP2	2.3
DN22571_c0_g1_i1	#N/A	#N/A	-10.59	MLRR1_PLAF7	9.8
DN22571_c0_g1_i7	#N/A	#N/A	-1.71	MLRR1_PLAF7	5
DN22572_c1_g1_i12	#N/A	#N/A	-8.99	SREK1_MOUSE	0.00036
DN22572_c1_g1_i13	#N/A	#N/A	-10.93	SREK1_MOUSE	0.00036
DN22580_c1_g1_i5	#N/A	#N/A	10.81	PRR37_ORYSI	7.90E-65
DN22585_c1_g1_i6	#N/A	#N/A	-1.7	ATXR3_ARATH	2.00E-147
DN22585_c1_g3_i6	#N/A	#N/A	-2.92	ATXR3_ARATH	1.10E-63
DN22588_c2_g1_i5	#N/A	#N/A	-1.51	MYO17_ARATH	0
DN22597_c0_g1_i9	#N/A	#N/A	-1.7	PSME4_ARATH	0
DN22599_c1_g2_i3	#N/A	#N/A	0.6	SR43C_ARATH	1.00E-142
DN22602_c0_g2_i3	#N/A	#N/A	-2.42	EMB8_PICGL	1.30E-39
DN22602_c0_g2_i6	#N/A	#N/A	-10.8	EMB8_PICGL	6.20E-40
DN22617_c1_g1_i4	#N/A	2.04	#N/A	EIX2_SOLLC	4.80E-86
DN22618_c1_g1_i8	#N/A	1.28	#N/A	KN7D_ARATH	0

Isoform ID	Log$_2$(#60tr/nt)	Log$_2$(#20tr/nt)	Log$_2$(#60tr/#20t	Annotation_protein	e_value_annotation
DN22620_c0_g1_i3	#N/A	8.04	#N/A	PHYLO_ARATH	0
DN22622_c0_g1_i15	#N/A	#N/A	-8.07	SPT61_ARATH	0
DN22623_c3_g1_i4	#N/A	#N/A	-0.92	TOR_ARATH	0
DN22626_c1_g1_i27	#N/A	#N/A	-4	MLH3_ARATH	9.00E-128
DN22629_c1_g1_i2	#N/A	#N/A	-2.59	TSS_ARATH	0
DN22640_c1_g1_i4	#N/A	#N/A	-0.92	TI236_ARATH	0
DN22640_c1_g1_i6	#N/A	#N/A	-1.18	TI236_ARATH	0
DN22643_c0_g1_i1	#N/A	2.59	#N/A	LIN1_LOTJA	1.80E-79
DN22652_c1_g1_i5	#N/A	7.95	#N/A	MIK2_ARATH	2.20E-50
DN22654_c2_g1_i10	#N/A	1.32	#N/A	Y5343_ARATH	0
DN22654_c2_g3_i4	#N/A	1.09	#N/A	Y5343_ARATH	2.00E-77
DN22664_c0_g2_i1	#N/A	#N/A	1.57	DEAHB_ARATH	0
DN22667_c1_g1_i1	#N/A	#N/A	-1.22	NRPB1_ARATH	0
DN22672_c1_g1_i11	#N/A	#N/A	1.56	AB4C_ARATH	0
DN22672_c1_g1_i2	#N/A	#N/A	2.03	AB4C_ARATH	0
DN22673_c1_g1_i7	#N/A	#N/A	-1.68	JMJ16_ARATH	0
DN22682_c2_g4_i4	#N/A	#N/A	-1.83	SNL3_ARATH	1.20E-27
DN22685_c2_g1_i15	#N/A	7.96	#N/A	HUA2_ARATH	2.40E-61
DN22689_c2_g1_i4	#N/A	#N/A	-1.12	PIEZO_ARATH	0
DN22691_c5_g1_i7	#N/A	-0.75	#N/A	.	#N/A
DN22699_c1_g1_i2	#N/A	#N/A	-1.32	TSS_ARATH	0
DN22699_c1_g1_i7	#N/A	#N/A	-1.44	TSS_ARATH	0
DN22700_c2_g1_i6	#N/A	#N/A	-2.76	BTAF1_ARATH	0
DN22704_c1_g6_i1	#N/A	#N/A	-1	CLASP_ARATH	0
DN22706_c0_g1_i12	#N/A	9	#N/A	DPOE1_ARATH	0
DN22707_c2_g1_i4	#N/A	#N/A	-1.32	UBP13_ARATH	5.90E-17
DN22708_c2_g2_i3	#N/A	#N/A	-1.04	VILI2_ARATH	0
DN22714_c0_g1_i2	#N/A	1.18	#N/A	MSH6_ARATH	3.40E-07
DN22716_c1_g1_i1	#N/A	#N/A	-1.84	BCHB_ARATH	0
DN22723_c1_g1_i4	#N/A	#N/A	-1.13	SPK1_ARATH	0
DN22727_c1_g1_i3	#N/A	#N/A	-1.75	UBP12_ARATH	0
DN22728_c0_g2_i5	#N/A	#N/A	-0.94	CESA3_ARATH	0
DN22731_c1_g1_i2	#N/A	#N/A	-1.93	DRL27_ARATH	1.00E-50
DN22731_c1_g1_i7	#N/A	#N/A	-2.22	DRL27_ARATH	1.50E-50
DN22731_c1_g1_i8	#N/A	#N/A	-1.82	DRL27_ARATH	6.20E-51
DN22734_c1_g1_i11	#N/A	#N/A	-2.84	PRT6_ARATH	0
DN22734_c1_g1_i12	#N/A	#N/A	-2.14	PRT6_ARATH	0
DN22736_c1_g1_i2	#N/A	#N/A	-1.57	ACC1_ARATH	0
DN22739_c1_g1_i14	#N/A	#N/A	-7.54	.	#N/A

Isoform ID	Log_2(#60tr/nt)	Log_2(#20tr/nt)	Log_2(#60tr/#20t	Annotation_protein	e_value_annotation
DN22741_c1_g4_i3	#N/A	#N/A	-0.85	AB1D_ARATH	0
DN22743_c2_g1_i3	#N/A	#N/A	-1.11	MOR1_ARATH	0
DN22743_c2_g1_i7	#N/A	#N/A	3.56	MOR1_ARATH	4.60E-22
DN22744_c2_g1_i16	#N/A	7.42	#N/A	C3H7_ARATH	6.40E-28
DN22744_c2_g1_i2	#N/A	#N/A	-1.1	C3H7_ARATH	5.00E-140
DN22745_c1_g1_i2	#N/A	#N/A	-1.01	KDM5_CAEBR	9.00E-120
DN22747_c4_g1_i2	#N/A	1.94	#N/A	ILA_ARATH	0
DN22749_c2_g1_i4	#N/A	#N/A	-0.91	TRA1_DROME	0
DN22750_c2_g1_i2	#N/A	#N/A	-0.87	DEK1_ARATH	0
DN22755_c1_g1_i1	#N/A	#N/A	-1.65	CLAH2_ARATH	0
DN22755_c1_g1_i3	#N/A	#N/A	-1.18	CLH1_ORYSJ	6.00E-128
DN22759_c2_g2_i3	#N/A	#N/A	-1.34	CHLH_ORYSI	7.00E-132
DN22759_c2_g6_i1	#N/A	#N/A	-1.53	CHLH_ARATH	1.40E-96
DN22759_c2_g6_i2	#N/A	#N/A	-1.21	CHLH_ARATH	2.00E-99
DN22760_c2_g2_i2	#N/A	#N/A	-1.67	SYD_ARATH	1.50E-18
DN22761_c9_g2_i9	#N/A	#N/A	-0.99	BRM_ARATH	0
DN22763_c6_g1_i2	#N/A	#N/A	-0.92	GRV2_ARATH	0
DN22769_c1_g1_i8	#N/A	#N/A	-0.75	FAB1B_ARATH	0
DN22770_c2_g2_i2	#N/A	2.19	-2.46	RPP2B_ARATH	5.00E-39
DN22770_c2_g2_i3	#N/A	3.25	#N/A	DSC1_ARATH	1.40E-41
DN22770_c2_g2_i8	#N/A	#N/A	-2.92	RPP2B_ARATH	5.00E-39
DN22770_c3_g1_i21	#N/A	2.88	-3.04	RUN1_VITRO	1.20E-76
DN22778_c6_g1_i2	#N/A	#N/A	8.62	DRL21_ARATH	7.90E-39
DN22778_c6_g1_i6	#N/A	#N/A	-1.31	R13L1_ARATH	1.60E-61
DN3390_c0_g1_i1	#N/A	#N/A	1.87	MIRA_SYNDU	1.20E-16
DN3407_c0_g1_i1	#N/A	-0.89	#N/A	WDR20_CAEEL	7.7
DN3427_c0_g1_i1	#N/A	#N/A	0.58	.	#N/A
DN7123_c0_g1_i1	#N/A	#N/A	0.58	NDUA5_ARATH	1.80E-91
DN8056_c0_g1_i1	#N/A	#N/A	0.86	Y5278_ARATH	7.10E-38
DN17414_c0_g2_i2	13.26	#N/A	#N/A	.	#N/A
DN20055_c0_g1_i20	11.84	#N/A	11.91	DRM2_ORYSJ	0
DN19935_c0_g1_i1	11.51	#N/A	11.58	FERON_ARATH	0
DN21779_c0_g1_i16	11.28	10.91	#N/A	Y005_SYNY3	4.20E-39
DN16691_c0_g6_i5	11.27	8.08	3.14	RLMH_EUBE2	8.8
DN15939_c0_g1_i9	10.92	#N/A	#N/A	FK201_ARATH	8.10E-91
DN20211_c1_g1_i9	10.91	11.01	#N/A	ESYT1_HUMAN	7.20E-13
DN14384_c0_g1_i6	10.84	#N/A	#N/A	AB11G_ARATH	9.00E-103
DN14582_c0_g2_i6	10.84	#N/A	10.92	CKB1_CAEEL	3.5
DN7082_c0_g1_i1	10.77	7.81	2.97	MAD50_ORYSJ,	3.00E-119

Isoform ID	Log$_2$(#60tr/nt)	Log$_2$(#20tr/nt)	Log$_2$(#60tr/#20t	Annotation_protein	e_value_annotation
DN16256_c0_g1_i6	10.68	#N/A	#N/A	C7150_PRUMU	3.60E-96
DN15208_c1_g1_i10	10.65	#N/A	10.72	OTU11_ARATH	6.00E-119
DN14254_c0_g2_i4	10.62	#N/A	#N/A	FULL_VITVI	2.60E-89
DN21723_c0_g1_i12	10.41	#N/A	#N/A	EHD2_ARATH	6.40E-15
DN20958_c2_g1_i17	10.31	#N/A	#N/A	BAMS_GLYGL	0
DN17739_c0_g1_i3	10.3	#N/A	#N/A	TCPQ_ARATH	0
DN21622_c0_g1_i26	10.17	10.89	#N/A	ACR3_ARATH	2.00E-166
DN17739_c0_g1_i16	10.16	#N/A	#N/A	TCPQ_ARATH	0
DN14171_c0_g5_i1	10.1	9.29	#N/A	PER31_ARATH	4.00E-153
DN17336_c0_g1_i6	10.09	#N/A	#N/A	PHSB_ARATH	0
DN14378_c1_g1_i1	10.08	#N/A	#N/A	Y9096_DICDI	3.90E-06
DN18399_c1_g1_i8	10.08	#N/A	4.9	.	#N/A
DN16997_c0_g1_i29	10.07	#N/A	#N/A	.	#N/A
DN18077_c0_g1_i8	10.07	9.24	#N/A	PAB_ARATH	2.00E-160
DN21349_c0_g1_i10	10.05	9.81	#N/A	TI100_ARATH	2.00E-134
DN18765_c0_g1_i3	9.93	#N/A	#N/A	RM55_DROME	1.5
DN19594_c1_g4_i5	9.86	7.8	#N/A	SYP22_ARATH	3.00E-145
DN15198_c0_g1_i6	9.79	#N/A	9.86	COBRA_ARATH	0
DN18313_c0_g1_i2	9.74	#N/A	#N/A	UBA5_ARATH	0
DN12970_c0_g1_i5	9.72	9.34	#N/A	.	#N/A
DN17993_c0_g1_i8	9.7	#N/A	#N/A	THO4D_ARATH	1.20E-75
DN15456_c1_g3_i3	9.67	#N/A	#N/A	PUB43_ARATH	3.70E-54
DN20816_c1_g3_i4	9.63	#N/A	#N/A	CYT24_CATRO	3.00E-166
DN21556_c1_g1_i10	9.49	#N/A	#N/A	RGLG4_ARATH	6.00E-158
DN17416_c1_g4_i9	9.47	#N/A	#N/A	E70A1_ARATH	1.00E-102
DN22591_c1_g1_i12	9.47	#N/A	9.54	NUP1_ARATH	3.10E-49
DN19830_c0_g1_i2	9.44	#N/A	#N/A	M2K3_ARATH	0
DN22429_c0_g1_i2	9.36	#N/A	#N/A	SNX16_MOUSE	3.20E-09
DN21631_c1_g3_i1	9.34	#N/A	#N/A	TCP2_ARATH	2.40E-57
DN14374_c1_g1_i7	9.32	#N/A	#N/A	4CLL9_ARATH	4.00E-167
DN17084_c1_g2_i2	9.27	#N/A	9.34	.	#N/A
DN17529_c0_g4_i5	9.17	#N/A	#N/A	.	#N/A
DN15112_c1_g2_i5	9.15	9.93	#N/A	APRR3_ARATH	4.70E-07
DN16781_c0_g2_i3	9.14	#N/A	9.21	SNX1_ARATH	0
DN15092_c0_g1_i1	9.13	#N/A	#N/A	.	#N/A
DN16944_c0_g1_i4	9.11	#N/A	9.19	CB069_DANRE	7.70E-08
DN18222_c0_g2_i4	9.1	#N/A	#N/A	M3K1_ARATH	9.20E-30
DN17026_c0_g7_i1	9.04	#N/A	9.11	NOTC2_HUMAN	0.039
DN21974_c0_g1_i11	9.03	8.77	#N/A	DNLI4_ARATH	0

Isoform ID	Log_2(#60tr/nt)	Log_2(#20tr/nt)	Log_2(#60tr/#20t	Annotation_protein	e_value_annotation
DN20538_c1_g1_i8	8.99	#N/A	9.06	.	#N/A
DN20833_c0_g1_i16	8.95	#N/A	#N/A	SSY4_ARATH	4.40E-87
DN14516_c3_g1_i5	8.94	#N/A	#N/A	YIP4B_ARATH	9.00E-112
DN15334_c0_g1_i14	8.94	#N/A	#N/A	WAV3_ARATH	0
DN18230_c0_g2_i18	8.94	#N/A	#N/A	DRC3_MACFA	3.10E-09
DN19581_c0_g1_i7	8.92	#N/A	#N/A	OCT4_ARATH	0
DN17242_c0_g1_i10	8.88	8.38	#N/A	RS31_ARATH	1.90E-90
DN20716_c0_g1_i4	8.87	#N/A	#N/A	ABA2_PRUAR	2.10E-92
DN20889_c0_g1_i7	8.87	#N/A	#N/A	CNOT4_HUMAN	2.10E-46
DN14786_c0_g1_i6	8.85	#N/A	#N/A	MORF3_ARATH	3.50E-57
DN19117_c1_g1_i7	8.77	#N/A	#N/A	Y5994_ARATH	6.00E-156
DN21749_c0_g1_i5	8.72	#N/A	8.79	JAR4_NICAT	3.40E-38
DN14396_c0_g1_i3	8.68	#N/A	#N/A	IBMP_CAMVP	0
DN19377_c0_g1_i6	8.66	8.87	#N/A	TSTC_DICDI	4.80E-06
DN17040_c0_g5_i1	8.61	#N/A	8.68	S2540_XENLA	9.20E-06
DN18751_c0_g1_i8	8.61	#N/A	#N/A	MBP2C_ARATH	3.00E-79
DN21903_c1_g2_i5	8.61	#N/A	#N/A	RPOZ_RICFE	0.71
DN22183_c0_g1_i13	8.6	#N/A	#N/A	FUCO2_ARATH	1.60E-96
DN21084_c0_g1_i12	8.58	#N/A	8.65	AMY3_ARATH	3.90E-31
DN16549_c0_g3_i2	8.55	#N/A	#N/A	.	#N/A
DN22150_c0_g1_i1	8.55	#N/A	8.62	VAC14_ARATH	0
DN16591_c0_g3_i14	8.53	#N/A	#N/A	PEX10_ARATH	2.00E-152
DN15290_c1_g1_i12	8.45	#N/A	#N/A	TPPII_ARATH	6.00E-134
DN19124_c0_g1_i3	8.45	8	#N/A	BABA2_DANRE	3.80E-18
DN19724_c0_g2_i6	8.45	#N/A	#N/A	ADO3_ARATH	0
DN15622_c0_g1_i8	8.42	#N/A	8.49	NAC82_ARATH	1.00E-56
DN15063_c0_g1_i19	8.41	#N/A	8.48	SIRB_ARATH	7.00E-79
DN17976_c0_g2_i3	8.38	#N/A	8.45	UBX2_YEAST	0.00014
DN17026_c0_g7_i2	8.37	#N/A	6.54	NOTC2_HUMAN	0.17
DN20857_c0_g1_i7	8.35	8.8	#N/A	RF298_ARATH	0.086
DN16649_c0_g1_i2	8.32	#N/A	#N/A	SP1L1_ARATH	1.60E-34
DN15580_c0_g2_i10	8.31	#N/A	#N/A	.	#N/A
DN15136_c0_g1_i2	8.3	#N/A	#N/A	FRS5_ARATH	7.60E-24
DN18442_c0_g4_i8	8.29	#N/A	#N/A	Y2433_ARATH	3.00E-141
DN14929_c1_g1_i10	8.27	#N/A	#N/A	.	#N/A
DN22079_c0_g1_i9	8.27	#N/A	#N/A	XYN1_ARATH	0
DN15690_c0_g1_i3	8.22	#N/A	#N/A	ARFB_ORYSJ	4.00E-18
DN19159_c1_g2_i2	8.22	#N/A	#N/A	RPC5_HUMAN	5.30E-27
DN21677_c1_g1_i1	8.14	#N/A	8.21	GAUT6_ARATH	8.00E-105

Isoform ID	Log_2(#60tr/nt)	Log_2(#20tr/nt)	Log_2(#60tr/#20t	Annotation_protein	e_value_annotation
DN18739_c1_g1_i19	8.11	#N/A	#N/A	GRF3_ORYSJ	4.30E-47
DN19870_c0_g1_i5	8.09	7.9	#N/A	HSF24_SOLPE	6.20E-73
DN16782_c0_g1_i3	8.08	#N/A	#N/A	.	#N/A
DN15466_c0_g1_i3	8.06	#N/A	#N/A	.	#N/A
DN18522_c0_g1_i2	8.02	#N/A	#N/A	MAT2B_PONAB	4.30E-13
DN18036_c0_g2_i5	7.97	#N/A	#N/A	PCO4_ARATH	2.00E-130
DN19185_c0_g2_i7	7.97	7.9	#N/A	.	#N/A
DN21148_c0_g3_i6	7.97	7.6	#N/A	VOZ1_ARATH	1.00E-56
DN18867_c0_g1_i7	7.94	#N/A	#N/A	RVE1_ARATH	4.70E-08
DN14183_c0_g1_i2	7.89	#N/A	#N/A	GATA_STRAW	0.51
DN22137_c0_g1_i5	7.88	#N/A	#N/A	BRE1A_ARATH	1.40E-79
DN16441_c1_g2_i10	7.86	#N/A	5.22	RFA2B_ARATH	9.60E-81
DN22137_c0_g1_i13	7.86	#N/A	#N/A	BRE1A_ARATH	0
DN21837_c0_g1_i8	7.85	#N/A	#N/A	FPP_SOLLC	8.00E-122
DN18952_c0_g1_i4	7.84	#N/A	#N/A	.	#N/A
DN17531_c0_g2_i11	7.77	#N/A	#N/A	OFT13_ARATH	0
DN21979_c1_g3_i1	7.75	#N/A	#N/A	ALN_ARATH	5.80E-69
DN14288_c0_g5_i9	7.64	#N/A	7.71	SNMP1_DROGR	1.3
DN19064_c0_g1_i7	7.63	#N/A	#N/A	LAC17_ARATH	3.00E-73
DN17948_c1_g3_i1	7.54	#N/A	#N/A	FBL53_ARATH	3.00E-147
DN16965_c0_g1_i13	7.49	#N/A	#N/A	PAP7_ARATH	2.00E-37
DN18136_c0_g1_i4	7.36	#N/A	#N/A	SIA2_ARATH	0
DN14475_c1_g1_i3	6.72	6.16	#N/A	SEOA_ARATH	2.00E-102
DN21556_c1_g1_i8	6.66	#N/A	6.78	RGLG4_ARATH	3.00E-158
DN18346_c0_g1_i7	6.54	#N/A	#N/A	COL14_ARATH	1.20E-30
DN22090_c1_g2_i1	6.43	#N/A	8.53	BLH1_ARATH	1.00E-126
DN20112_c0_g1_i5	6.35	#N/A	#N/A	GIL1_ARATH	1.30E-40
DN17484_c0_g1_i28	6.23	#N/A	#N/A	.	#N/A
DN19726_c0_g1_i7	6.22	#N/A	8.25	TRM52_VITVI	0
DN17755_c0_g1_i2	6.16	#N/A	#N/A	LP14_BACIU	1.10E-22
DN18446_c0_g1_i11	6.15	#N/A	#N/A	FREE1_ARATH	1.00E-131
DN17254_c0_g3_i8	6.11	#N/A	#N/A	LIMYB_ARATH	3.80E-24
DN21028_c0_g1_i7	6.08	#N/A	#N/A	UFSP_ARATH	0
DN15403_c1_g1_i5	5.92	#N/A	#N/A	RSMG_LACH4	7.90E-59
DN21013_c0_g1_i1	5.9	5.59	#N/A	CHLD_PEA	0
DN20806_c0_g2_i1	5.88	#N/A	#N/A	SGPL_ARATH	2.00E-143
DN19017_c0_g1_i1	5.86	#N/A	#N/A	3MG1_ECOLI	4.60E-50
DN14069_c2_g1_i3	5.31	#N/A	#N/A	SDG40_ARATH	5.90E-05
DN17075_c0_g2_i2	5.26	4.86	#N/A	RL10_EUPES	6.00E-123

Isoform ID	Log_2(#60tr/nt)	Log_2(#20tr/nt)	Log_2(#60tr/#20t	Annotation_protein	e_value_annotation
DN15371_c1_g2_i9	5.12	4.47	#N/A	BH068_ARATH	1.50E-67
DN15819_c1_g1_i18	5.12	4.92	#N/A	RPP2B_ARATH	0.00021
DN18221_c0_g1_i15	5.11	#N/A	4.41	BBD1_ARATH	1.00E-141
DN18716_c0_g1_i2	5.04	#N/A	#N/A	.	#N/A
DN21013_c0_g1_i3	4.94	5.18	#N/A	CHLD_PEA	0
DN20172_c0_g1_i11	4.87	4.76	#N/A	APA2_ARATH	0
DN13711_c0_g1_i1	4.79	#N/A	#N/A	NAC83_ARATH	6.70E-21
DN20252_c0_g1_i1	4.77	#N/A	#N/A	SCP46_ARATH	1.20E-93
DN21512_c0_g1_i13	4.74	#N/A	#N/A	F135A_PONAB	1.20E-56
DN15402_c0_g1_i2	4.71	#N/A	#N/A	CB23_SOYBN	0
DN12601_c0_g1_i2	4.35	#N/A	#N/A	SBP1_ANTMA	6.70E-40
DN15022_c0_g1_i4	4.3	#N/A	#N/A	GDL82_ARATH	0
DN17259_c0_g1_i3	4.23	#N/A	3.35	5MMP_ARATH	2.30E-38
DN21716_c0_g1_i16	4.01	#N/A	#N/A	RPT3_ARATH	1.60E-46
DN16000_c0_g1_i2	3.82	#N/A	#N/A	XTH9_ARATH	4.00E-157
DN19878_c1_g5_i2	3.41	#N/A	2.46	COL16_ARATH	2.00E-101
DN15510_c1_g4_i2	3.1	#N/A	#N/A	.	#N/A
DN19005_c0_g3_i3	3.06	#N/A	#N/A	.	#N/A
DN15369_c0_g4_i3	2.77	#N/A	3.24	LIRP1_ARATH	6.40E-16
DN17431_c0_g1_i25	2.77	#N/A	3.38	CP110_HUMAN	0.83
DN18267_c0_g1_i6	2.75	#N/A	#N/A	PPP7_ARATH	0
DN17240_c0_g2_i10	2.69	#N/A	2.67	IFR_SOYBN	0
DN16796_c0_g1_i19	2.65	#N/A	#N/A	Y1666_ARATH	8.00E-170
DN21296_c0_g3_i10	2.63	#N/A	#N/A	LACS1_ARATH	0
DN21649_c1_g2_i17	2.62	#N/A	#N/A	Y1960_ARATH	0
DN17573_c0_g1_i8	2.39	#N/A	#N/A	AAPC_CENCI	3.90E-12
DN14561_c0_g1_i6	2.36	#N/A	1.66	.	#N/A
DN22201_c0_g1_i6	2.31	#N/A	#N/A	LOX4_SOYBN	0
DN19025_c0_g4_i1	2.26	#N/A	2.57	PIP22_PEA	2.50E-94
DN18077_c0_g1_i2	2.11	#N/A	#N/A	PAB_ARATH	8.80E-80
DN17078_c0_g1_i4	2.02	#N/A	#N/A	FUT1_PEA	2.40E-83
DN17722_c0_g1_i4	1.96	#N/A	#N/A	NIPA6_ARATH	2.00E-170
DN14587_c1_g3_i2	1.81	#N/A	1.99	TAUE3_ARATH	0
DN18257_c1_g1_i6	1.79	#N/A	#N/A	BPM3_ARATH	0
DN18907_c0_g1_i10	1.73	2.05	#N/A	SSG1_IPOBA	0
DN15102_c0_g1_i7	1.67	#N/A	#N/A	TM208_DANRE	2.60E-25
DN17120_c4_g1_i9	1.67	#N/A	#N/A	SYC_CHLPN	1.2
DN18921_c0_g3_i3	1.66	#N/A	#N/A	GIL1_ARATH	0
DN19407_c0_g5_i1	1.65	#N/A	1.55	14312_ARATH	5.00E-162

Isoform ID	Log_2(#60tr/nt)	Log_2(#20tr/nt)	Log_2(#60tr/#20t	Annotation_protein	e_value_annotation
DN20604_c0_g2_i8	1.64	#N/A	1.44	.	#N/A
DN15369_c0_g4_i2	1.63	#N/A	2.22	LIRP1_ARATH	6.10E-30
DN19920_c0_g3_i5	1.61	#N/A	1.27	BGH3B_BACO1	1.80E-90
DN22086_c0_g2_i11	1.59	#N/A	1.17	KAD2_ARATH	1.00E-123
DN20325_c0_g1_i14	1.56	#N/A	#N/A	SBT1_SOYBN	0
DN21870_c0_g1_i2	1.55	#N/A	#N/A	AVP_VIGRR	0
DN21293_c1_g3_i1	1.53	#N/A	#N/A	TLC1_ARATH	0
DN18841_c0_g1_i9	1.52	#N/A	#N/A	ACINU_MOUSE	1.20E-19
DN16997_c0_g1_i6	1.5	#N/A	#N/A	.	#N/A
DN16720_c0_g5_i10	1.48	#N/A	0.7	DHAS_SYNY3	2.00E-123
DN17898_c0_g2_i3	1.48	#N/A	1.48	UNCL_ARATH	2.00E-157
DN11939_c0_g1_i2	1.44	#N/A	1.47	NTPA_PEA	0
DN15511_c0_g3_i4	1.44	#N/A	#N/A	SYIM_ARATH	0
DN16339_c0_g2_i6	1.43	#N/A	0.95	ADHL3_ARATH	0
DN21979_c1_g1_i69	1.43	#N/A	1.32	ALN_ARATH	0
DN15369_c0_g4_i1	1.38	#N/A	1.91	.	#N/A
DN11303_c0_g1_i1	1.37	#N/A	1.59	PMAT1_ARATH	1.40E-92
DN15369_c0_g4_i7	1.37	#N/A	2.13	.	#N/A
DN22201_c0_g1_i7	1.37	#N/A	1.49	LOX4_SOYBN	0
DN19254_c0_g2_i4	1.36	#N/A	1.94	LOX21_SOLTU	0
DN17538_c0_g2_i5	1.34	#N/A	1.17	YCF23_PORPU	8.50E-57
DN19991_c0_g3_i2	1.31	#N/A	1.27	FTSH_CHLT2	2
DN16521_c0_g2_i3	1.29	2.1	#N/A	PUP18_ARATH	0.99
DN13835_c2_g2_i8	1.27	#N/A	1.11	PORA_CUCSA	0
DN15664_c0_g2_i2	1.26	#N/A	1.32	MEE14_ARATH	7.80E-64
DN13773_c0_g2_i6	1.19	#N/A	1.25	ADT3_ARATH	1.80E-63
DN16883_c0_g1_i3	1.19	#N/A	0.92	SPEE_BIFLO	5.10E-10
DN17555_c0_g3_i8	1.19	#N/A	#N/A	ENO2_HEVBR	9.00E-154
DN17120_c4_g1_i13	1.18	#N/A	0.78	SYC_CHLPN	1.2
DN20009_c1_g3_i1	1.16	#N/A	1.66	DIR21_ARATH	9.80E-45
DN20297_c0_g1_i13	1.15	#N/A	#N/A	DNLJ_RHOCS	1.1
DN13778_c0_g1_i2	1.09	#N/A	1.22	.	#N/A
DN18929_c0_g1_i2	1.08	#N/A	0.92	RL321_ARATH	4.10E-83
DN16210_c0_g5_i1	1.06	#N/A	1.28	Y2179_DICDI	3.30E-13
DN22201_c0_g1_i11	1.06	#N/A	1.46	LOX4_SOYBN	0
DN19958_c0_g1_i17	1.04	#N/A	#N/A	CYP38_ARATH	0
DN21293_c1_g3_i8	1.04	#N/A	#N/A	TLC1_ARATH	0
DN21795_c1_g3_i14	1.04	#N/A	1.37	DOG1_YEAST	5.1
DN15049_c0_g2_i1	1.03	#N/A	1.35	SYE_CYTH3	0.009

Isoform ID	Log_2(#60tr/nt)	Log_2(#20tr/nt)	Log_2(#60tr/#20t	Annotation_protein	e_value_annotation
DN15831_c0_g2_i3	1.01	#N/A	1.06	CP31A_ARATH	2.50E-32
DN20502_c0_g3_i9	1	#N/A	0.9	ATPF2_SPIOL	1.10E-79
DN21795_c1_g3_i1	1	#N/A	1.02	HMCS_ARATH	0
DN19333_c0_g5_i1	0.98	#N/A	1.16	SGT3_SOYBN	0
DN13827_c0_g2_i5	0.97	#N/A	0.99	RBG2_ARATH	4.60E-51
DN17311_c0_g2_i5	0.94	#N/A	#N/A	TMK1_ARATH	1.10E-83
DN19663_c0_g2_i2	0.93	#N/A	1.41	ERD15_SOYBN	2.40E-88
DN21543_c0_g2_i1	0.93	#N/A	0.74	SAT3_ARATH	2.00E-144
DN16471_c0_g1_i7	0.92	#N/A	#N/A	NDF5_ARATH	3.20E-82
DN17120_c4_g1_i3	0.92	#N/A	#N/A	ACSC_KOMXY	0.23
DN19239_c0_g3_i5	0.92	#N/A	0.99	CAMT_POPTM	8.00E-134
DN15618_c0_g2_i1	0.89	#N/A	1	ACP1_CASGL	3.00E-43
DN15104_c0_g7_i3	0.83	#N/A	1.15	C7A15_ARATH	0
DN13270_c0_g1_i2	0.81	#N/A	#N/A	RR10_SPIOL	7.50E-80
DN21089_c0_g2_i5	-0.76	#N/A	#N/A	MAOP4_ARATH	5.00E-99
DN13612_c0_g5_i2	-0.79	#N/A	#N/A	DTX40_ARATH	0
DN20216_c0_g1_i11	-0.82	#N/A	#N/A	CAO_ARATH	0
DN20341_c0_g1_i11	-0.85	#N/A	#N/A	SYT4_ARATH	1.70E-19
DN16424_c0_g1_i1	-0.86	#N/A	#N/A	CO9_TAKRU	1.4
DN15490_c0_g1_i8	-0.89	#N/A	-1	AOX4_ARATH	2.00E-172
DN16601_c0_g1_i1	-0.9	#N/A	#N/A	SYK_UREU1	1.4
DN16841_c0_g3_i5	-0.93	#N/A	-1.02	CSLE1_ARATH	0
DN21118_c0_g2_i2	-0.93	#N/A	-1.25	SMBP2_MESAU	9.00E-109
DN18624_c0_g2_i3	-0.94	#N/A	#N/A	PMI2_ARATH	7.50E-51
DN20825_c0_g2_i10	-0.94	#N/A	-0.85	SPD1_ARATH	0
DN21781_c0_g1_i8	-0.94	#N/A	-0.81	PPDK_MESCR	0
DN16210_c0_g4_i5	-0.95	-0.75	#N/A	DJC76_ARATH	1.30E-29
DN19655_c0_g1_i1	-0.95	#N/A	-1.21	FRI2_SOYBN	6.00E-166
DN20390_c0_g1_i7	-0.96	#N/A	-0.8	DAPF_THEEB	1
DN18779_c1_g3_i3	-0.97	-0.97	#N/A	RCA_VIGRR	1.00E-102
DN16244_c1_g1_i11	-0.98	#N/A	#N/A	Y4597_ARATH	4.00E-99
DN20602_c2_g2_i7	-0.99	#N/A	#N/A	Y1669_ARATH	2.30E-75
DN16424_c0_g1_i4	-1	#N/A	#N/A	NO66_BRUMA	0.62
DN16210_c0_g2_i8	-1.01	#N/A	#N/A	DJC76_ARATH	8.70E-29
DN18257_c1_g1_i9	-1.02	#N/A	#N/A	BPM3_ARATH	0
DN21781_c0_g1_i1	-1.02	#N/A	-0.65	PPDK_MESCR	0
DN14225_c0_g4_i3	-1.03	#N/A	-0.85	TPS7_RICCO	0
DN14968_c0_g2_i4	-1.04	#N/A	#N/A	SVP_ARATH	1.10E-66
DN13911_c0_g4_i1	-1.05	#N/A	#N/A	DXR_MENPI	0

Isoform ID	Log_2(#60tr/nt)	Log_2(#20tr/nt)	Log_2(#60tr/#20t	Annotation_protein	e_value_annotation
DN20144_c0_g3_i3	-1.05	#N/A	#N/A	Y5986_ARATH	4.10E-37
DN21645_c0_g1_i27	-1.06	-0.81	#N/A	TPPA_ARATH	0
DN22184_c0_g3_i5	-1.06	#N/A	#N/A	LHY_ARATH	7.00E-139
DN16085_c1_g1_i5	-1.07	#N/A	-0.95	LNK2_ARATH	1.40E-98
DN15160_c1_g1_i2	-1.08	#N/A	-1.49	WSD1_ARATH	6.20E-39
DN18355_c0_g1_i2	-1.08	#N/A	#N/A	KUA1_ARATH	3.40E-35
DN15209_c1_g1_i3	-1.1	#N/A	#N/A	HLS1L_ARATH	0.18
DN19207_c0_g4_i20	-1.1	-1.14	#N/A	ATPA_NEOSM	4.5
DN21565_c0_g3_i2	-1.1	#N/A	#N/A	HC173_ARATH	3.80E-18
DN14968_c0_g2_i18	-1.11	#N/A	-1.17	SVP_ARATH	1.10E-65
DN19531_c0_g2_i15	-1.12	#N/A	#N/A	Y2028_DICDI	1.50E-20
DN21308_c0_g1_i19	-1.12	#N/A	-1.1	Y1457_ARATH	4.00E-116
DN20831_c0_g2_i9	-1.13	-1.38	#N/A	WTR32_ARATH	7.00E-171
DN15732_c0_g1_i1	-1.16	#N/A	#N/A	BOR4_ARATH	0
DN20436_c0_g1_i8	-1.18	#N/A	#N/A	PLCD6_ARATH	0
DN19977_c0_g1_i5	-1.2	#N/A	#N/A	FTSH2_ORYSJ	1.60E-54
DN16374_c0_g1_i6	-1.21	#N/A	#N/A	UN933_ARATH	6.00E-180
DN16638_c0_g2_i7	-1.21	-1.19	#N/A	MBCD_SOLTU	0
DN12927_c0_g1_i1	-1.23	#N/A	#N/A	XYNC_ASPTN	4.6
DN21781_c0_g1_i5	-1.23	#N/A	-0.82	PPDK_FLABR	2.00E-163
DN15732_c0_g1_i4	-1.24	-1.22	#N/A	BOR4_ARATH	0
DN17902_c0_g1_i17	-1.24	#N/A	-1.42	HMGB1_ARATH	6.70E-50
DN21308_c0_g1_i13	-1.26	#N/A	-0.9	Y1457_ARATH	2.00E-100
DN21940_c0_g2_i2	-1.26	#N/A	-1.03	M3K20_ARATH	2.00E-105
DN18355_c0_g1_i3	-1.31	#N/A	#N/A	.	#N/A
DN18384_c0_g4_i1	-1.33	#N/A	-1.38	.	#N/A
DN21781_c0_g1_i14	-1.33	#N/A	-1.14	PPDK_MESCR	2.00E-123
DN21781_c0_g1_i12	-1.36	#N/A	-1.2	PPDK_MESCR	2.90E-93
DN14258_c0_g5_i1	-1.38	#N/A	#N/A	XERIC_ARATH	1.60E-16
DN20976_c0_g1_i9	-1.38	-1.75	#N/A	RN135_HUMAN	0.53
DN17057_c0_g1_i2	-1.41	#N/A	#N/A	.	#N/A
DN19591_c0_g3_i2	-1.45	-1.06	#N/A	PUB33_ARATH	1.60E-12
DN12752_c0_g1_i6	-1.46	-1.3	#N/A	.	#N/A
DN14666_c0_g5_i2	-1.46	-0.99	#N/A	MIP1B_ARATH	4.20E-10
DN13861_c0_g1_i2	-1.48	#N/A	-1.2	E70H1_ARATH	1.00E-174
DN17057_c0_g1_i15	-1.5	-0.88	#N/A	BEPA_VIBCH	1.1
DN19021_c0_g1_i4	-1.53	#N/A	-1.23	MLO6_ARATH	0
DN18752_c0_g2_i13	-1.54	#N/A	#N/A	NADD_IDILO	0.057
DN18973_c0_g2_i1	-1.54	#N/A	#N/A	HHT1_ARATH	0

Isoform ID	Log_2(#60tr/nt)	Log_2(#20tr/nt)	Log_2(#60tr/#20t	Annotation_protein	e_value_annotation
DN14206_c0_g1_i18	-1.61	#N/A	#N/A	ACCO_PRUMU	1.40E-13
DN18609_c0_g1_i25	-1.67	#N/A	#N/A	WTR18_ARATH	1.00E-102
DN20733_c1_g1_i7	-1.69	#N/A	#N/A	MCTP2_MOUSE	0.13
DN16671_c0_g1_i7	-1.73	-11.11	9.52	NRX3_ARATH	2.00E-171
DN20816_c1_g3_i1	-1.75	#N/A	#N/A	CYT24_CATRO	6.00E-166
DN18836_c0_g1_i11	-1.81	#N/A	-1.67	HSFA3_ARATH	2.80E-21
DN16956_c0_g4_i1	-1.93	#N/A	-1.66	MIP1A_ARATH	8.20E-24
DN18269_c0_g1_i6	-1.94	#N/A	#N/A	5NTC_DICDI	4.30E-68
DN22021_c0_g1_i7	-1.96	-1.13	#N/A	SUT31_ARATH	0
DN15903_c0_g1_i1	-2.01	#N/A	#N/A	PGL1A_ARATH	3.90E-73
DN15732_c0_g1_i7	-2.11	#N/A	#N/A	BOR4_ARATH	0
DN14959_c0_g4_i2	-2.26	#N/A	#N/A	EFTU_BUCCC	0.44
DN22021_c0_g1_i10	-2.27	#N/A	#N/A	SUT31_ARATH	0
DN21189_c0_g1_i8	-2.35	#N/A	#N/A	CYPC_STRCO	0.00031
DN20171_c1_g2_i7	-2.36	#N/A	-2.27	XYLL2_ARATH	6.70E-87
DN20484_c0_g2_i14	-2.36	#N/A	#N/A	SYAP_POPTR	0
DN13742_c0_g1_i1	-2.41	#N/A	#N/A	C93C1_SOYBN	0
DN12570_c0_g1_i2	-2.58	#N/A	#N/A	MAN4_SOLLC	2.00E-166
DN16502_c0_g1_i26	-2.66	#N/A	#N/A	HEXP_LEIMA	9.50E-24
DN15431_c0_g6_i7	-2.88	#N/A	-2.71	WAVH1_ARATH	8.00E-133
DN17409_c0_g1_i1	-3.24	#N/A	#N/A	COL16_ARATH	3.90E-84
DN20972_c0_g1_i24	-3.65	#N/A	#N/A	RSZ32_ARATH	1.20E-59
DN18859_c0_g1_i3	-4.08	#N/A	#N/A	Y5713_ARATH	0
DN19626_c0_g2_i1	-4.24	#N/A	#N/A	RFP4B_DANRE	9.4
DN17829_c0_g2_i8	-4.43	#N/A	#N/A	FIE2_ORYSJ	0
DN22427_c0_g1_i2	-5.19	#N/A	-5.56	CXB2_BOVIN	0.33
DN22427_c0_g1_i13	-5.33	#N/A	-5.06	SIS8_ARATH	2.70E-49
DN22427_c0_g1_i18	-5.37	#N/A	-5.83	TRPA_BACC0	0.76
DN22427_c0_g1_i15	-5.41	#N/A	-4.94	SIS8_ARATH	2.70E-49
DN16729_c0_g3_i5	-5.42	#N/A	-5.13	SDP1_ARATH	0
DN20310_c0_g1_i13	-5.8	#N/A	-6.12	RHF2A_ARATH	6.00E-159
DN18446_c0_g1_i35	-5.97	#N/A	#N/A	FREE1_ARATH	4.00E-139
DN18932_c1_g2_i9	-6.08	#N/A	#N/A	STY46_ARATH	0
DN21828_c0_g1_i11	-6.18	#N/A	-6.65	SDIS_COMTE	0.75
DN18976_c0_g3_i4	-6.21	#N/A	#N/A	CDF2_ARATH	1.70E-91
DN20451_c1_g1_i5	-6.45	#N/A	-6.42	CRK10_ARATH	0
DN22043_c0_g1_i21	-6.74	#N/A	-6.84	.	#N/A
DN21109_c0_g1_i24	-6.89	#N/A	#N/A	CPNA2_ARATH	6.00E-107
DN20576_c1_g2_i5	-7	#N/A	-6.84	MBR2_ARATH	1.40E-36

Isoform ID	Log_2(#60tr/nt)	Log_2(#20tr/nt)	Log_2(#60tr/#20t	Annotation_protein	e_value_annotation
DN19823_c0_g1_i2	-7.07	#N/A	#N/A	NAC8_ARATH	3.00E-170
DN15013_c2_g1_i8	-7.5	#N/A	#N/A	MURG_CLOB6	0.19
DN16057_c0_g1_i3	-7.54	-5.72	#N/A	ADS3_ARATH	1.00E-172
DN19594_c1_g3_i6	-7.64	#N/A	#N/A	KPYA_RICCO	2.50E-85
DN20720_c0_g1_i1	-7.7	#N/A	-8.84	MBD9_ARATH	0
DN17253_c0_g1_i10	-7.76	#N/A	-8.31	AAPC_CENCI	6.00E-73
DN15677_c0_g8_i6	-7.8	#N/A	-8.1	DTX16_ARATH	1.10E-72
DN22374_c0_g2_i9	-7.8	#N/A	#N/A	LUMI_ARATH	6.90E-48
DN17165_c0_g4_i6	-7.84	#N/A	#N/A	RAD50_ARATH	5.00E-147
DN21136_c0_g1_i2	-7.84	#N/A	#N/A	CHX19_ARATH	0
DN18569_c0_g1_i2	-7.89	#N/A	-7.59	SECA_SYNP6	0.15
DN16182_c0_g1_i3	-7.91	#N/A	#N/A	PPA28_ARATH	2.00E-123
DN14727_c0_g5_i1	-7.95	#N/A	#N/A	SDR1_ARATH	3.80E-50
DN20463_c1_g1_i1	-7.95	#N/A	#N/A	ERDL5_ARATH	2.00E-103
DN21189_c0_g1_i23	-7.97	#N/A	#N/A	.	#N/A
DN21786_c0_g1_i19	-7.97	#N/A	-9.34	RPP2B_ARATH	0.013
DN20140_c1_g1_i12	-8.01	#N/A	#N/A	MMSA_ARATH	0
DN14374_c1_g1_i5	-8.06	#N/A	-8.75	4CLL9_ARATH	4.00E-164
DN15653_c0_g1_i4	-8.07	#N/A	#N/A	MUC70_ARATH	1.40E-62
DN17767_c0_g1_i12	-8.07	#N/A	#N/A	P2C49_ARATH	4.20E-69
DN20291_c1_g1_i11	-8.07	#N/A	-9.13	CLPB1_ARATH	0
DN22616_c0_g2_i3	-8.1	#N/A	-8.94	BEM46_SCHPO	0.081
DN22121_c0_g1_i7	-8.11	#N/A	#N/A	GAP22_ARATH	3.80E-28
DN22736_c0_g1_i8	-8.13	#N/A	#N/A	ACC1_ARATH	2.90E-30
DN15140_c0_g1_i17	-8.14	#N/A	-9.37	CRK25_ARATH	6.00E-116
DN17384_c1_g4_i8	-8.14	-8.07	#N/A	GID1B_ARATH	0
DN20117_c0_g1_i10	-8.14	#N/A	#N/A	CAAT4_ARATH	5.40E-42
DN17247_c0_g3_i7	-8.15	#N/A	#N/A	TCP2_ARATH	3.80E-45
DN14476_c0_g2_i20	-8.17	#N/A	#N/A	GRS15_ARATH	1.10E-42
DN21411_c0_g1_i1	-8.18	#N/A	#N/A	PP171_ARATH	0
DN19606_c0_g1_i21	-8.19	#N/A	#N/A	PDK_ARATH	3.00E-117
DN22270_c0_g1_i15	-8.2	#N/A	#N/A	PUB42_ARATH	4.90E-10
DN17517_c0_g2_i3	-8.24	#N/A	#N/A	PSI1_ARATH	0
DN13871_c0_g2_i20	-8.25	#N/A	#N/A	.	#N/A
DN14256_c0_g2_i18	-8.29	#N/A	-7.51	.	#N/A
DN17075_c0_g2_i9	-8.29	-8.22	#N/A	RL10_EUPES	6.00E-123
DN14682_c0_g2_i1	-8.33	#N/A	#N/A	SYT_SULSY	5.3
DN18476_c0_g3_i7	-8.33	-8.26	#N/A	WRK70_SOLLC	6.50E-37
DN17907_c1_g1_i3	-8.34	#N/A	#N/A	BET11_ARATH	3.20E-66

Isoform ID	Log_2(#60tr/nt)	Log_2(#20tr/nt)	Log_2(#60tr/#20t	Annotation_protein	e_value_annotation
DN21378_c2_g2_i2	-8.41	#N/A	-7.65	RB3GP_DROME	7.80E-29
DN14124_c0_g1_i8	-8.46	#N/A	#N/A	GPDP1_ARATH	2.00E-116
DN18446_c0_g1_i22	-8.46	#N/A	#N/A	FREE1_ARATH	4.00E-139
DN16495_c0_g2_i1	-8.48	#N/A	#N/A	PREP2_ARATH	4.00E-107
DN22056_c0_g2_i13	-8.48	#N/A	#N/A	GLR32_ARATH	2.80E-42
DN17527_c0_g1_i11	-8.51	#N/A	-9.47	P2C38_ARATH	5.00E-176
DN17225_c1_g3_i14	-8.53	#N/A	#N/A	.	#N/A
DN20289_c0_g2_i12	-8.57	#N/A	#N/A	MSH1_ARATH	0
DN19215_c0_g1_i13	-8.6	#N/A	-9.34	PP276_ARATH	0
DN22412_c0_g1_i4	-8.61	#N/A	-9.66	PICBP_ARATH	2.70E-27
DN19108_c0_g1_i8	-8.62	#N/A	-8.6	HFA1D_ARATH	4.00E-169
DN17680_c0_g2_i7	-8.68	#N/A	#N/A	PRT1_ARATH	2.00E-103
DN15694_c0_g4_i10	-8.69	#N/A	-7.88	HAX1_BOVIN	6.1
DN16449_c0_g1_i22	-8.69	#N/A	#N/A	FBL3_ARATH	2.00E-105
DN22037_c1_g1_i36	-8.7	#N/A	-7.94	RUMI_CULQU	3.10E-25
DN22071_c0_g1_i12	-8.7	-8.63	#N/A	TMK1_ARATH	0
DN16767_c0_g2_i3	-8.74	#N/A	#N/A	.	#N/A
DN14893_c0_g2_i2	-8.75	#N/A	-9.16	AGD14_ARATH	5.70E-21
DN14171_c0_g5_i2	-8.81	#N/A	#N/A	PER31_ARATH	1.00E-152
DN17778_c0_g1_i15	-8.85	#N/A	-9.29	YUGF_BACSU	0.00011
DN21472_c1_g1_i2	-8.86	#N/A	-9.89	TRP5_ARATH	2.00E-128
DN14296_c0_g1_i10	-8.92	#N/A	#N/A	CPNE6_HUMAN	0.29
DN18653_c0_g1_i12	-8.96	#N/A	#N/A	LHP1_ARATH	1.80E-65
DN20362_c0_g1_i22	-8.98	#N/A	#N/A	DHE4_GIAIN	2.00E-105
DN17889_c0_g4_i2	-9.03	#N/A	-8.88	CYB5_TOBAC	1.30E-79
DN22627_c2_g1_i20	-9.03	#N/A	#N/A	XPO1A_ARATH	4.00E-157
DN21252_c0_g1_i3	-9.05	#N/A	-10.07	.	#N/A
DN22081_c1_g1_i6	-9.06	#N/A	#N/A	SPY_ARATH	0
DN20192_c1_g4_i7	-9.18	#N/A	-10.89	ECT2_ARATH	1.00E-87
DN11415_c0_g1_i2	-9.22	#N/A	#N/A	DODA_BETVU	5.80E-36
DN16499_c0_g6_i3	-9.23	#N/A	#N/A	PDV2_ARATH	2.40E-77
DN22466_c1_g2_i7	-9.23	#N/A	-10	DCP5_ARATH	6.00E-155
DN17179_c0_g2_i4	-9.3	#N/A	#N/A	RIBA3_ARATH	0
DN20545_c0_g1_i16	-9.3	#N/A	#N/A	CCS1_ARATH	1.00E-165
DN19255_c0_g2_i8	-9.47	#N/A	-9.49	RBM27_HUMAN	0.29
DN21374_c1_g2_i5	-9.56	#N/A	-8.6	HSTC_ARATH	1.00E-154
DN14373_c0_g1_i8	-9.62	#N/A	-8.75	DAPA_SOYBN	0
DN17123_c0_g1_i8	-9.72	#N/A	#N/A	MSRA4_ARATH	1.40E-67
DN18077_c0_g1_i12	-9.8	#N/A	#N/A	PAB_ARATH	5.00E-118

Isoform ID	Log_2(#60tr/nt)	Log_2(#20tr/nt)	Log_2(#60tr/#20t	Annotation_protein	e_value_annotation
DN20271_c0_g2_i10	-9.81	#N/A	-9.83	C2D61_ARATH	1.60E-50
DN21193_c0_g1_i9	-9.89	#N/A	#N/A	EFNMT_XENLA	9.00E-99
DN15769_c0_g1_i6	-10.04	#N/A	#N/A	RAC1_LOTJA	8.00E-121
DN18077_c0_g1_i13	-10.07	#N/A	#N/A	PAB_ARATH	2.10E-77
DN20216_c0_g1_i17	-10.16	#N/A	-10.45	CAO_ARATH	0
DN22402_c1_g1_i25	-10.57	#N/A	#N/A	AAH2_SOYBN	0
DN20926_c0_g1_i17	-10.77	#N/A	-11.9	AB39G_ORYSJ	3.60E-62
DN15208_c1_g1_i9	-11.02	#N/A	-10.9	OTU11_ARATH	6.00E-119
DN17886_c0_g1_i15	-11.23	-11.16	#N/A	ALFC5_ARATH	3.70E-63
DN18940_c0_g1_i5	-11.85	-11.78	#N/A	MDHC_MEDSA	0
